# Diversity of parasitoid wasps (Insecta, Hymenoptera) in oilseed rape fields in Serbia

**DOI:** 10.3897/BDJ.11.e110118

**Published:** 2023-12-05

**Authors:** Milan Plećaš, Vladimir Žikić, Korana Kocić, Jelisaveta Čkrkić, Anđeljko Petrović, Željko Tomanović

**Affiliations:** 1 University of Belgrade, Faculty of Biology, Studentski Trg 16, 11000 Belgrade, Serbia University of Belgrade, Faculty of Biology, Studentski Trg 16 11000 Belgrade Serbia; 2 University of Niš, Faculty of Sciences and Mathematics, Department of Biology with Ecology, Višegradska 33, P.O. Box 224, 18000, Niš, Serbia University of Niš, Faculty of Sciences and Mathematics, Department of Biology with Ecology, Višegradska 33, P.O. Box 224, 18000 Niš Serbia; 3 Centre for Biodiversity Genomics, University of Guelph, 50 Stone Road, N1G 2W1, Guelph, Ontario, Canada Centre for Biodiversity Genomics, University of Guelph, 50 Stone Road N1G 2W1, Guelph, Ontario Canada

**Keywords:** oilseed rape pests, host species, Balkans, ecological interactions, biological control agents

## Abstract

**Background:**

Oilseed rape is an important crop grown worldwide and used for various purposes, including oil extraction and animal feed. In Europe, there are six major pest species and several other minor pests that can significantly affect oilseed rape production, requiring growers to effectively control them in order to ensure crop yield. The host-parasitoid complexes of these pests have been studied in detail and recorded mainly in western, central and northern Europe. As an abundant source of pollen and nectar, oilseed rape may also be attractive to other parasitoids that do not have direct trophic interactions with oilseed rape pest species. The aim of this study is to fill the knowledge gap regarding the wider parasitoid community in oilseed rape fields, particularly in southern Europe.

**New information:**

During the two-year study, a total of 3135 specimens of primary and secondary parasitoids were sampled, of which 2855 were found in oilseed rape fields and 280 in semi-natural habitats. We found 153 taxa, of which 119 were found in oilseed rape fields and 87 in semi-natural habitats. We identified 31 genera (33 species) as parasitoids of oilseed rape pests, 54 genera (97 species) parasitising non-pest species and 10 genera (23 species) as possible parasitoids of oilseed rape pests. This study shows that the parasitoid community in oilseed rape fields is very diverse and that includes parasitoids of both oilseed rape pest and non-pest species.

## Introduction

Oilseed rape (*Brassicanapus* L.) is a very important crop, grown on more than 36 million hectares worldwide and more than 8 million hectares in Europe in 2021 (FAO 2022) and it is the third largest source of vegetable oil worldwide (Statista 2023). It is primarily used to extract oil from its seeds, which can be used for a variety of purposes, from food to making lubricants and soap, while the seed residue after oil extraction can also be used as animal feed (Alford 2003). In some countries, it is also an important break crop in cereal-dominated crop rotations (Angus et al. 2015). Similar to expanding global production, oilseed rape cultivation in Serbia increased from 5000–6000 ha at the end of the last century to more than 29,000 ha in 2022 (Sekulić and Kereši 2007, RZS 2023).

There are six major insect pest species of oilseed rape in Europe which growers often need to control to protect seed yield. In addition, there are a number of minor pests that may be of greater importance in some countries and at certain times of the year (Alford 2003, Williams 2010) (Table 1). Control of these pests is usually achieved through conventional pesticide use, but also through integrated pest management (IPM), as naturally occurring biological control agents, such as predators, parasitoids and pathogens can provide biocontrol and reduce pesticide use (Williams 2010). Parasitoids play particularly important role in pest control because their development is closely linked to their host species and show positive density-dependent patterns, while being specialised to one or few host species (Gunton and Pöyry 2016, Dainese et al. 2017). Higher parasitism rates (30–40%) can successfully suppress pest populations (Hokkanen 2008), although even lower rates (around 15%), especially when complemented with ground-dwelling predators, can also negatively affect pest abundance (Dainese et al. 2017). Semi-natural habitats that remain in agricultural landscapes are important for the conservation of these natural enemies, as they provide nectar resources, alternative host or prey species and overwintering sites (Landis et al. 2000, Bianchi et al. 2006) and their positive effect on oilseed rape pest suppression has been demonstrated (Thies and Tscharntke 1999, Dainese et al. 2017). However, oilseed rape itself can be a rich and highly attractive source of pollen and nectar for a wide range of insect species (Stanley et al. 2013), including parasitoids that use nectar as a food source (Heimpel and Jervis 2005, Varennes et al. 2016). Therefore, some parasitoid species may be present in oilseed rape fields without direct trophic interactions with known oilseed rape pests.

Host-parasitoid complexes for the aforementioned major and some minor pests have been extensively studied and recorded (Alford 2003, Williams 2010), but mainly based on data from two projects: BORIS (1997-2000, CT-96-1314) and MASTER (2001-2005, QLK5-CT-2001-01447), focusing mainly on western, central and northern Europe. Data from southern Europe and Serbia, in particular, have been largely absent. To the best of our knowledge, there has not been a comprehensive list of parasitoids and hyperparasitoids that occur in oilseed rape fields for this region. Todorov et al. (2022) provide a list of parasitoid species found in oilseed rape fields in Bulgaria with comments on perspectives for biological control, but only for the family Pteromalidae. Oilseed rape pests, on the other hand, are relatively well studied in Serbian fields from both taxonomical and traditional chemical management aspects (Kereši et al. 2007, Sekulić and Kereši 2007, Štrbac et al. 2007, Mitrović et al. 2008, Milovac et al. 2010, Graora et al. 2013, Milovanović et al. 2013, Graora et al. 2015, Sivčev et al. 2015, Sivčev et al. 2016, Milovac 2016, Marjanović and Prodanović 2021). Similarly, the parasitoid wasp fauna of other crops in Serbia, especially cereals and alfalfa, has also been well studied (Tomanović et al. 2003, Kavallieratos et al. 2004, Kavallieratos et al. 2005a, Kavallieratos et al. 2005b, Tomanović et al. 2008, Žikić et al. 2012, Tomanović et al. 2021), but currently, there is a gap in knowledge of the parasitoid wasp fauna in oilseed rape. Therefore, we aim here to explore the entire parasitoid community in oilseed rape to better understand the trophic interactions and the wider potential for biological control in agricultural landscapes.

## Materials and methods

**Figure 1.**
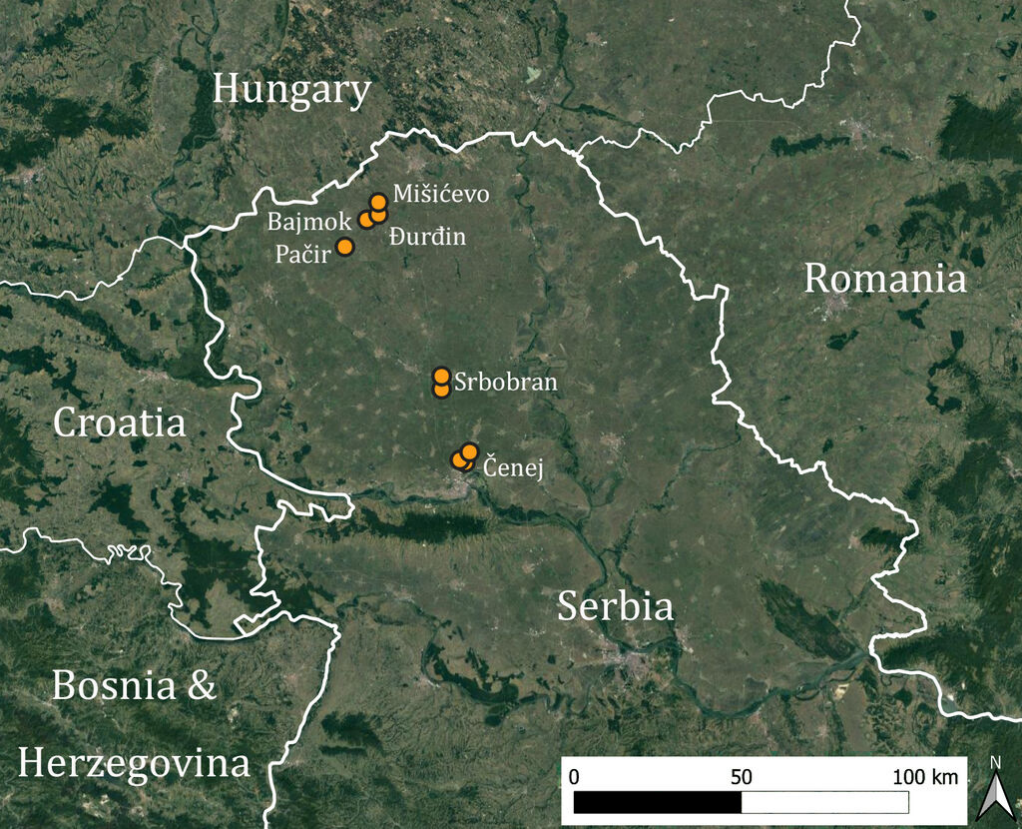
Map of the study area.

Sampling was conducted in two consecutive years with a similar sampling design. In 2018, we selected five oilseed rape fields in the southern part of the Bačka Region (Serbia) (Table 2, Fig. 1). A single sampling transect of 20 m length was established in each field. Two fields had adjacent herbaceous semi-natural habitats where an additional sampling transect was established. In 2019, four oilseed rape fields were selected in the northern part of the Bačka Region, each of which had an adjacent herbaceous or shrubland semi-natural habitat. Two sampling transects were established in each field, one near the field edge (2 m from the field edge) and the second deeper in the field (20 m from the field edge) and one transect was established in the adjacent semi-natural habitat. Two fields had a second nearby semi-natural habitat and one additional sampling transect was established in each of them.

Parasitoids and hyperparasitoids were collected multiple times using different methods during the period from mid-April to mid-June, i.e. from the beginning of the oilseed rape flowering to the pre-harvest stage of pod maturation. Along each transect, 20 sweeps were made at the top of the vegetation with a sweep net and the collected samples were immediately stored in 96% ethanol. Sweep-net samples were collected seven times in 2018 and five times in 2019. In addition, during oilseed rape flowering phase, three clusters of pan traps were placed along each transect, at the beginning, middle and at the end of the transect. Each cluster consisted of three coloured water-filled bowls (yellow, white and blue) that were placed on the ground when surrounding vegetation was low or mounted on a pole and raised to the top of the vegetation when vegetation was tall. Traps were left active for 72 hours in 2018 and 48 hours in 2019, after which samples were placed in 96% ethanol. In both years, pan-trap sampling was done three times, once in late April and twice in early May in 2018 and three times in mid- to late April in 2019. During each field visit, oilseed rape plants and other plants were systematically examined for the presence of aphids along each established transect, both in crop fields and in semi-natural habitats. If aphid colonies were found, they were placed in a mash-top plastic box for aphid parasitoids rearing. After four weeks, emerged parasitoids were transferred to 96% ethanol. In addition, in 2019 during pod maturation phase, we collected up to 100 oilseed rape pods per field transect and stored them in a mash-top plastic box to allow the emergence of pod midge parasitoids, which were then transferred to 96% ethanol. All collected parasitoids were identified using morphological identification keys (Suppl. material 1) to the lowest possible taxonomic level (species, genus or family). In case of identification to higher taxonomic levels, taxa were characterised and organised into different morphospecies (e.g. Eulophidae sp. 1, Eulophidae sp. 2 etc.). Voucher specimens are deposited in the collection of the Institute of Zoology, Faculty of Biology, University of Belgrade, Serbia. To establish consistency of occurrence of parasitoids in oilseed rape fields, we selected taxa/genera, based on two combined criteria across two years of sampling: 1) present in at least three fields (out of nine total) and 2) present with at least 10 individuals. We constructed species accumulation curves to assess completeness of our fauna survey in oilseed rape fields. We defined each combination of locality and sampling period as a “sampling event” which consisted of combined data from all sampling methods. To estimate extrapolated species richness for our study, we used Chao, First order Jackknife (Jack1), Second order Jackknife (Jack2) and Bootstrap estimates from the R package *vegan* (v.2.6-4; Oksanen et al. (2022)) within R Statistical Software (v.4.3.1; R Core Team (2023)).

## Checklists

### Checklist of parasitoid wasps found in oilseed rape fields and adjacent semi-natural habitats in Serbia

#### Bethylidae

B202BEDB-B8FB-57F3-9694-813D6A4AE296

#### Goniozus claripennis

(Förster, 1851)

2368AA26-8B28-5D50-9614-0229BC2DBE87

##### Materials

- **Type status:** Other material. **Occurrence:** individualCount: 1 male; behavior: primary parasitoids, larval; occurrenceID: 2100E2C9-88FF-50B0-96C1-065E02A65373; **Location:** country: Serbia; locality: Đurđin; **Event:** samplingProtocol: Pan traps; eventDate: 22-24.04.2019; habitat: oilseed rape

##### Parasite of

Tortricidae

##### Notes

oilseed rape pest host: unknown

#### Plastanoxus westwoodi

(Kieffer, 1914)

D7114F85-8961-55EF-B80B-319C0BC69D5D

##### Materials

- **Type status:** Other material. **Occurrence:** individualCount: 1 male; behavior: primary parasitoids, larval/pupal; occurrenceID: 69C5F48C-FA04-5E14-BD6A-6DCAFF0DB2BE; **Location:** country: Serbia; locality: Čenej; **Event:** samplingProtocol: Pan traps; eventDate: 24-27.04.2018; habitat: oilseed rape

##### Parasite of

Cucujidae

##### Notes

oilseed rape pest host: unknown

#### Braconidae

B7D00757-ED4E-5280-A117-89116BC25EC6

#### Apanteles sp. 1

629D5A8A-78E4-5A1B-BFD6-50F6AD7684F1

##### Materials

- **Type status:** Other material. **Occurrence:** individualCount: 8 males, 5 females; behavior: primary parasitoids, larval; occurrenceID: 670AD6EE-22E6-5A5C-8D72-C0625E726E99; **Location:** country: Serbia; locality: Čenej, Mišićevo, Pačir; **Event:** samplingProtocol: Sweep net; eventDate: 07.05.2018, 10.05.2018, 25.05.2018, 18.04.2019, 12.06.2019; habitat: oilseed rape, semi-natural habitat

##### Parasite of

Lepidoptera

##### Notes

oilseed rape pest host: unknown

#### Aphidius ervi

Haliday, 1834

DFC4B768-5A47-539A-BB70-6E50D3D5FF3C

##### Materials

- **Type status:** Other material. **Occurrence:** individualCount: 215 males, 60 females; behavior: primary parasitoids, larval; occurrenceID: C5B6BE40-91B1-5621-AC91-D69A68728B55; **Location:** country: Serbia; locality: Bajmok, Čenej, Đurđin, Srbobran; **Event:** samplingProtocol: Sweep net, Pan traps, Aphid colony; eventDate: 24-27.04.2018, 04-07.05.2018, 07-10.05.2018, 25.05.2018, 22-24.04.2019, 23.05.2019; habitat: oilseed rape, semi-natural habitat

##### Parasite of

*Myzuspersicae*

##### Notes

oilseed rape pest host: yes

#### Aphidius matricariae

Haliday, 1834

B5954636-FCB4-5D1C-8EDB-3BA0CACF1542

##### Materials

- **Type status:** Other material. **Occurrence:** individualCount: 72 males, 40 females; behavior: primary parasitoids, larval; occurrenceID: 3950E1EC-D843-5AB9-B17F-937164B61976; **Location:** country: Serbia; locality: Čenej, Srbobran; **Event:** samplingProtocol: Aphid colony; eventDate: 04.05.2018, 07.05.2018, 10.05.2018; habitat: oilseed rape

##### Parasite of

*Myzuspersicae*

##### Notes

oilseed rape pest host: yes

#### Aphidius sp. 1

2C151974-29EC-5052-973D-FD5478F0F7FD

##### Materials

- **Type status:** Other material. **Occurrence:** individualCount: 1 male, 1 female; behavior: primary parasitoids, larval; occurrenceID: 3E6F63DA-DB7D-5F1D-A55F-07289769670F; **Location:** country: Serbia; locality: Đurđin; **Event:** samplingProtocol: Aphid colony; eventDate: 05-23-19; habitat: oilseed rape

##### Parasite of

*Myzuspersicae*

##### Notes

oilseed rape pest host: unknown, possible

#### Binodoxys angelicae

(Haliday, 1833)

9C691365-92AB-50F4-80AB-D5D0EF106D2B

##### Materials

- **Type status:** Other material. **Occurrence:** individualCount: 1 male; behavior: primary parasitoids, larval; occurrenceID: EBAA0EF7-3F19-5404-9F10-5282D552F36F; **Location:** country: Serbia; locality: Mišićevo; **Event:** samplingProtocol: Aphid colony; eventDate: 05-23-19; habitat: oilseed rape

##### Parasite of

*Myzuspersicae*

##### Notes

oilseed rape pest host: yes

#### Blacus nigricornis

Haeselbarth, 1973

B9555EB1-7F31-5FEA-B676-20CFD6599174

##### Materials

- **Type status:** Other material. **Occurrence:** individualCount: 2 males, 1 female; behavior: primary parasitoids, larval; occurrenceID: 92167F97-BDA3-57A5-9DB1-203EF3FAB78D; **Location:** country: Serbia; locality: Čenej, Srbobran; **Event:** samplingProtocol: Sweep net, Pan traps; eventDate: 27.04.2018, 07-10.05.2018; habitat: oilseed rape, semi-natural habitat

##### Parasite of

*Meligethesaeneus*

##### Notes

oilseed rape pest host: yes

#### Bracon picticornis

(Wesmael, 1838)

BCA66B5B-73F6-5042-959A-0CF783091644

##### Materials

- **Type status:** Other material. **Occurrence:** individualCount: 1 male; behavior: primary parasitoids, larval; occurrenceID: BD9DB36F-E474-5431-B537-EF01E6550C2E; **Location:** country: Serbia; locality: Bajmok; **Event:** samplingProtocol: Pan traps; eventDate: 17-19.04.2019; habitat: oilseed rape

##### Parasite of

*Meligethesaeneus*

##### Notes

oilseed rape pest host: yes

#### Bracon variator

Nees, 1811

9592011C-C623-5E47-A5DE-768F1931058A

##### Materials

- **Type status:** Other material. **Occurrence:** individualCount: 2 males; behavior: primary parasitoids, larval; occurrenceID: 76500E7C-7739-54B4-9FB6-1460E32CCBB5; **Location:** country: Serbia; locality: Čenej; **Event:** samplingProtocol: Sweep net, Pan traps; eventDate: 04-07.05.2018, 10.05.2018; habitat: oilseed rape

##### Parasite of

*Ceutorhynchusassimilis*

##### Notes

oilseed rape pest host: yes

#### Chelonus oculator

(Fabricius, 1775)

33124C24-A41A-59A8-8690-9DBA0685EB8D

##### Materials

- **Type status:** Other material. **Occurrence:** individualCount: 1 male; behavior: primary parasitoids, egg/larval; occurrenceID: 981653E9-B91B-5795-8BE8-910E33C9FD2C; **Location:** country: Serbia; locality: Srbobran; **Event:** samplingProtocol: Pan traps; eventDate: 04-07.05.2018; habitat: semi-natural habitat

##### Parasite of

Lepidoptera, *Spodopteraexigua*

##### Notes

oilseed rape pest host: unknown

#### Choeras parasitellae

(Bouché, 1834)

28423E65-7A77-558C-9C37-77A0D5E642DA

##### Materials

- **Type status:** Other material. **Occurrence:** individualCount: 1 female; behavior: primary parasitoids, larval; occurrenceID: A0968BCD-EB9E-5A04-A16E-734D2F88B3E8; **Location:** country: Serbia; locality: Čenej; **Event:** samplingProtocol: Sweep net; eventDate: 05-10-18; habitat: oilseed rape

##### Parasite of

Lepidoptera

##### Notes

oilseed rape pest host: unknown

#### Cotesia glomerata

(Linnaeus, 1758)

E1762B44-82BC-571D-A9B3-A2A610352A4D

##### Materials

- **Type status:** Other material. **Occurrence:** individualCount: 2 males, 1 female; behavior: primary parasitoids, larval; occurrenceID: 5DA837C7-FAF4-5E5D-BBAF-2679FD8A7656; **Location:** country: Serbia; locality: Srbobran; **Event:** samplingProtocol: Sweep net, Pan traps; eventDate: 27.04.2018, 04-07.05.2018, 25.05.2018; habitat: oilseed rape

##### Parasite of

*Pieris* spp.

##### Notes

oilseed rape pest host: yes

#### Cotesia vestalis

(Haliday, 1834)

FD1B02AF-FD35-5BBA-B610-21258213F850

##### Materials

- **Type status:** Other material. **Occurrence:** individualCount: 1 female; behavior: primary parasitoids, larval; occurrenceID: D9CFE5F0-9B48-5FAB-887A-A19948176C6C; **Location:** country: Serbia; locality: Pačir; **Event:** samplingProtocol: Sweep net; eventDate: 05-10-19; habitat: semi-natural habitat

##### Parasite of

*Plutellaxylostella*

##### Notes

oilseed rape pest host: yes

#### Dacnusa sp. 1

B5B9784D-A546-502C-83A9-ED45CE757771

##### Materials

- **Type status:** Other material. **Occurrence:** individualCount: 1 female; behavior: primary parasitoids, larval; occurrenceID: 9D21083D-9666-5D29-9B82-B599A478426B; **Location:** country: Serbia; locality: Čenej; **Event:** samplingProtocol: Pan traps; eventDate: 04-07.05.2018; habitat: oilseed rape

##### Parasite of

*Phytomyzarufipes*?

##### Notes

oilseed rape pest host: unknown

#### Diaeretiella rapae

(McIntosh,1855)

D635B757-1A8F-5CE1-AB16-C40856DBF82D

##### Materials

- **Type status:** Other material. **Occurrence:** individualCount: 816 males, 427 females; behavior: primary parasitoids, larval; occurrenceID: 9B75C3AA-8CF9-5C70-A0AD-910DE1A7E9EA; **Location:** country: Serbia; locality: Bajmok, Čenej, Đurđin, Mišićevo, Pačir, Srbobran; **Event:** samplingProtocol: Sweep net, Pan traps, Aphid colony; eventDate: 24-27.04.2018, 04-07.05.2018, 07-10.05.2018, 25.05.2018, 17-19.04.2019, 22-24.04.2019, 08.05.2019, 10.05.2019, 23.05.2019, 12.06.2019, 13.06.2019; habitat: oilseed rape, semi-natural habitat

##### Parasite of

*Myzuspersicae*

##### Notes

oilseed rape pest host: yes

#### Diospilus capito

(Nees, 1834)

2DA2CF80-6612-597E-B954-3D64687ABB63

##### Materials

- **Type status:** Other material. **Occurrence:** individualCount: 1 female; behavior: primary parasitoids, larval; occurrenceID: 38221A3B-0E37-569C-8E40-2F3A41611E99; **Location:** country: Serbia; locality: Srbobran; **Event:** samplingProtocol: Sweep net; eventDate: 04-27-18; habitat: semi-natural habitat

##### Parasite of

*Meligethesaeneus*

##### Notes

oilseed rape pest host: yes

#### Ephedrus persicae

Froggatt, 1904

807058F2-DAB6-5822-88D2-7C252D4BA7AF

##### Materials

- **Type status:** Other material. **Occurrence:** individualCount: 1 male; behavior: primary parasitoids, larval; occurrenceID: 894A5022-E488-56DD-8F1B-563D4D8E8E23; **Location:** country: Serbia; locality: Čenej; **Event:** samplingProtocol: Aphid colony; eventDate: 05-10-18; habitat: oilseed rape

##### Parasite of

*Myzuspersicae*

##### Notes

oilseed rape pest host: yes

#### Eubazus sigalphoides

(Marshall, 1889)

9C68C00D-4DD7-5A38-858F-21AEE7246C3D

##### Materials

- **Type status:** Other material. **Occurrence:** individualCount: 44 males, 8 females; behavior: primary parasitoids, larval; occurrenceID: AD0C09B2-CCD1-5DFD-882F-0BE15DE7EDE2; **Location:** country: Serbia; locality: Čenej, Srbobran; **Event:** samplingProtocol: Sweep net, Pan traps, Aphid colony; eventDate: 24-27.04.2018, 04.05.2018, 07.05.2018, 10.05.2018, 25.05.2018, 27.04.2018; habitat: oilseed rape

##### Parasite of

*Meligethesaeneus*

##### Notes

oilseed rape pest host: yes

#### Eubazus sp. 1

D92AE959-5A49-58F6-AF52-828F52A8AE18

##### Materials

- **Type status:** Other material. **Occurrence:** individualCount: 1 male, 1 female; behavior: primary parasitoids, larval; occurrenceID: C0808E15-FC75-5133-9529-253FC39B21F3; **Location:** country: Serbia; locality: Srbobran; **Event:** samplingProtocol: Sweep net; eventDate: 27.04.2018, 04.05.2018; habitat: oilseed rape, semi-natural habitat

##### Parasite of

Curculionidae, *Pissodes* spp.

##### Notes

oilseed rape pest host: unknown, possible

#### Habrobracon hebetor

(Say, 1836)

6B1CE6CE-70E1-5AA6-9C82-59E89E6007D0

##### Materials

- **Type status:** Other material. **Occurrence:** individualCount: 3 males; behavior: primary parasitoids, larval; occurrenceID: D172A12C-83BF-5A6C-882D-B89A6B2A56B7; **Location:** country: Serbia; locality: Čenej; **Event:** samplingProtocol: Sweep net, Pan traps; eventDate: 27.04.2018, 04-07.05.2018, 07-10.05.2018; habitat: oilseed rape

##### Parasite of

*Plutellaxylostella*

##### Notes

oilseed rape pest host: yes

#### Lysiphlebus fabarum

(Marshall, 1896)

283C0B74-6DB7-5E09-9C76-B0D000AE12DC

##### Materials

- **Type status:** Other material. **Occurrence:** individualCount: 7 males, 8 females; behavior: primary parasitoids, larval; occurrenceID: 5E911AD9-61F7-5767-82D5-2C5F9C00A3CC; **Location:** country: Serbia; locality: Pačir, Srbobran; **Event:** samplingProtocol: Pan traps, Aphid colony; eventDate: 07-10.05.2018, 23.05.2019; habitat: semi-natural habitat

##### Parasite of

*Myzuspersicae*

##### Notes

oilseed rape pest host: yes

#### Microctonus sp. 1

CB2BF726-127D-52F7-8685-BFCF1E00D29E

##### Materials

- **Type status:** Other material. **Occurrence:** individualCount: 1 female; behavior: primary parasitoids, adult; occurrenceID: 2C6284BF-9E5E-5350-9DC9-0302DA217FF7; **Location:** country: Serbia; locality: Čenej; **Event:** samplingProtocol: Sweep net; eventDate: 04-27-18; habitat: oilseed rape

##### Parasite of

*Psylliodeschrysocephala*

##### Notes

oilseed rape pest host: unknown, possible

#### Microctonus sp. 2

55E34A5C-F186-5BFC-B989-E874C2B6DDCC

##### Materials

- **Type status:** Other material. **Occurrence:** individualCount: 1 male; behavior: primary parasitoids, adult; occurrenceID: 5867C306-6324-5480-92E0-45222448E6D9; **Location:** country: Serbia; locality: Čenej; **Event:** samplingProtocol: Sweep net; eventDate: 05-10-18; habitat: semi-natural habitat

##### Parasite of

*Psylliodeschrysocephala*

##### Notes

oilseed rape pest host: unknown, possible

#### Microplitis sp. 1

998BEB5F-4E94-5D9D-A722-23FE874096AC

##### Materials

- **Type status:** Other material. **Occurrence:** individualCount: 4 males; behavior: primary parasitoids, larval; occurrenceID: DAB4FA8A-9140-5590-AC18-0A40980767C9; **Location:** country: Serbia; locality: Čenej; **Event:** samplingProtocol: Sweep net; eventDate: 07.05.2018, 10.05.2018, 25.05.2018; habitat: oilseed rape, semi-natural habitat

##### Parasite of

Lepidoptera

##### Notes

oilseed rape pest host: unknown

#### Peristenus sp. 1

55CB899E-DC9C-5A43-8448-AD9B4421485F

##### Materials

- **Type status:** Other material. **Occurrence:** individualCount: 2 males; behavior: primary parasitoids, adult; occurrenceID: FF098CFC-54DE-59E1-8DEF-F0B13AA0C33C; **Location:** country: Serbia; locality: Mišićevo, Pačir; **Event:** samplingProtocol: Sweep net; eventDate: 25.04.2019, 10.05.2019; habitat: semi-natural habitat

##### Parasite of

Hemiptera, Miridae

##### Notes

oilseed rape pest host: unknown

#### Peristenus sp. 2

39D774CA-9E90-5310-A757-1963DB5AEF09

##### Materials

- **Type status:** Other material. **Occurrence:** individualCount: 1 male; behavior: primary parasitoids, adult; occurrenceID: 134DA297-FDA0-5920-8599-D9951B634070; **Location:** country: Serbia; locality: Pačir; **Event:** samplingProtocol: Sweep net; eventDate: 05-10-19; habitat: semi-natural habitat

##### Parasite of

Hemiptera, Miridae

##### Notes

oilseed rape pest host: unknown

#### Praon volucre

(Haliday, 1933)

F505DF9D-EBAA-5D75-88E5-F6D915A20393

##### Materials

- **Type status:** Other material. **Occurrence:** individualCount: 17 males, 12 females; behavior: primary parasitoids, larval; occurrenceID: 87BA73CA-4D26-5BD6-957F-D51999F207CC; **Location:** country: Serbia; locality: Čenej, Srbobran; **Event:** samplingProtocol: Sweep net, Pan traps, Aphid colony; eventDate: 27.04.2018, 04-07.05.2018, 10.05.2018; habitat: oilseed rape, semi-natural habitat

##### Parasite of

*Myzuspersicae*

##### Notes

oilseed rape pest host: yes

#### Schizoprymnus obscurus

(Nees, 1816)

076664E7-5B8B-5D65-B062-6ED6149CE776

##### Materials

- **Type status:** Other material. **Occurrence:** individualCount: 6 males; behavior: primary parasitoids, larval; occurrenceID: E16619A8-405D-5DB5-865C-9A40D688C840; **Location:** country: Serbia; locality: Čenej, Mišićevo; **Event:** samplingProtocol: Sweep net, Aphid colony; eventDate: 20.04.2018, 10.05.2018, 12.06.2019; habitat: oilseed rape, semi-natural habitat

##### Parasite of

*Ceutorhynchus* spp.

##### Notes

oilseed rape pest host: yes

#### Townesilitus bicolor

(Wesmael, 1835)

8FDFA7E8-DF6E-58EC-8E9E-F436D8D5DBB7

##### Materials

- **Type status:** Other material. **Occurrence:** individualCount: 29 males, 4 females; behavior: primary parasitoids, adult; occurrenceID: 6C1B44DA-6B18-5F2A-AA95-117FA9C8DC30; **Location:** country: Serbia; locality: Čenej, Pačir, Srbobran; **Event:** samplingProtocol: Sweep net, Pan traps; eventDate: 24-27.04.2018, 07.05.2018, 10.05.2018, 25.05.2018, 10.05.2019; habitat: oilseed rape, semi-natural habitat

##### Parasite of

*Phyllotreta* spp.

##### Notes

oilseed rape pest host: yes

#### Triaspis thoracica

(Curtis, 1860)

90F752AD-311B-5D1D-8C61-C305CCC3CA3F

##### Materials

- **Type status:** Other material. **Occurrence:** individualCount: 17 males, 53 females; behavior: primary parasitoids, larval; occurrenceID: 31AF200E-A505-5FF0-BE16-9F8C0D660AAC; **Location:** country: Serbia; locality: Čenej, Srbobran; **Event:** samplingProtocol: Sweep net; eventDate: 27.04.2018, 07.05.2018, 10.05.2018, 25.05.2018; habitat: oilseed rape

##### Parasite of

Chrysomelidae, *Bruchus* spp.

##### Notes

oilseed rape pest host: unknown

#### Ceidae

DD8300BF-7BD7-5124-BFDE-DF57B6E3AB2B

#### Cea pulicaris

Walker, 1837

C126A1D9-F1B8-5EB1-A4F8-BD3FD080FFB4

##### Materials

- **Type status:** Other material. **Occurrence:** individualCount: 1 male; behavior: primary parasitoids, larval; occurrenceID: 8C00D2C6-6FA5-5B26-9D99-ADDCC9E1D372; **Location:** country: Serbia; locality: Srbobran; **Event:** samplingProtocol: Pan traps; eventDate: 07-10.05.2018

##### Parasite of

Agromyzidae, *Phytomyza* spp.

##### Notes

oilseed rape pest host: unknown

#### Spalangiopelta sp. 1

2B85E8F1-6C52-5AF4-9F6C-9B6E12669F09

##### Materials

- **Type status:** Other material. **Occurrence:** individualCount: 8 males, 4 females; behavior: primary parasitoids, larval; occurrenceID: E6ABC350-A923-5C28-BBBA-2465C9111481; **Location:** country: Serbia; locality: Čenej, Đurđin, Mišićevo, Srbobran; **Event:** samplingProtocol: Sweep net, Pan traps; eventDate: 27.04.2018, 04.05.2018, 07.05.2018, 25.05.2018, 22-24.04.2019, 25.04.2019, 12.06.2019; habitat: oilseed rape, semi-natural habitat

##### Parasite of

Agromyzidae, Drosophilidae (miners)

##### Notes

oilseed rape pest host: unknown

#### Ceraphronidae

AE5540DC-8D14-5F6C-B84F-C8CFA2B20CEF

#### Ceraphronidae sp. 1

A198921E-C6E0-5E34-AB12-7AE2FE3254AC

##### Materials

- **Type status:** Other material. **Occurrence:** individualCount: 2 males, 1 female; behavior: primary parasitoids, larval; occurrenceID: 6977C9C7-9469-5D55-9EB8-7B56EF27769E; **Location:** country: Serbia; locality: Čenej, Srbobran; **Event:** samplingProtocol: Sweep net, Pan traps; eventDate: 24-27.04.2018, 07-10.05.2018, 25.05.2018; habitat: oilseed rape, semi-natural habitat

##### Parasite of

Cecidomyiidae, Hemiptera, Neuroptera, Thysanoptera

##### Notes

oilseed rape pest host: unknown

#### Ceraphronidae sp. 2

03E60AD3-56D4-5E9C-B9A5-5B18239EB521

##### Materials

- **Type status:** Other material. **Occurrence:** individualCount: 1 male; behavior: primary parasitoids, larval; occurrenceID: 3C03AC9C-9FF1-5178-9F37-501881047C03; **Location:** country: Serbia; locality: Đurđin; **Event:** samplingProtocol: Pan traps; eventDate: 22-24.04.2019; habitat: semi-natural habitat

##### Parasite of

Cecidomyiidae, Hemiptera, Neuroptera, Thysanoptera

##### Notes

oilseed rape pest host: unknown

#### Aphanogmus abdominalis

(Thomson, 1858)

8B42031D-C693-5134-AC43-90F3E5E9F721

##### Materials

- **Type status:** Other material. **Occurrence:** individualCount: 9 males, 16 females; behavior: primary parasitoids, larval; occurrenceID: 67963D58-F8DD-5A8E-9A48-9100C67EC5F9; **Location:** country: Serbia; locality: Čenej, Srbobran; **Event:** samplingProtocol: Sweep net, Pan traps; eventDate: 24-27.04.2018, 04-07.05.2018, 07-10.05.2018, 13.06.2018; habitat: oilseed rape, semi-natural habitat

##### Parasite of

*Dasineurabrassicae*

##### Notes

oilseed rape pest host: yes

#### Ceraphron sp. 1

5B48B399-8C8C-5997-B62B-FE6595EFE0BF

##### Materials

- **Type status:** Other material. **Occurrence:** individualCount: 1 male; behavior: primary parasitoids, larval; occurrenceID: D3738836-AF98-56D5-8C8F-4906B24752AD; **Location:** country: Serbia; locality: Srbobran; **Event:** samplingProtocol: Pan traps; eventDate: 04-07.05.2018; habitat: semi-natural habitat

##### Parasite of

Cecidomyiidae, Hemiptera, Neuroptera, Thysanoptera

##### Notes

oilseed rape pest host: unknown

#### Ceraphron sp. 2

3E9AD894-FD7C-583A-9EEB-FA78335FA13F

##### Materials

- **Type status:** Other material. **Occurrence:** individualCount: 3 males; behavior: primary parasitoids, larval; occurrenceID: 4F1DC248-EF72-5FB1-9F45-07BCA5B0BBD8; **Location:** country: Serbia; locality: Čenej, Đurđin; **Event:** samplingProtocol: Pan traps; eventDate: 24-27.04.2018, 22-24.04.2019; habitat: oilseed rape

##### Parasite of

Cecidomyiidae, Hemiptera, Neuroptera, Thysanoptera

##### Notes

oilseed rape pest host: unknown

#### Chalcididae

42B8E552-7290-508D-9C0E-518CDC0AA2AB

#### Brachymeria tibialis -group

Steffan, 1958

E7B63F0C-FD5B-55BF-8CF8-A1EBF87D8E4C

##### Materials

- **Type status:** Other material. **Occurrence:** individualCount: 2 males; behavior: primary/secondary parasitoids, larval/pupal; occurrenceID: BB4DEADB-F72A-5BD6-B3F9-A220BDF54A02; **Location:** country: Serbia; locality: Đurđin, Srbobran; **Event:** samplingProtocol: Sweep net, Pan traps; eventDate: 24-27.04.2018, 24.04.2019; habitat: oilseed rape, semi-natural habitat

##### Parasite of

Lepidoptera, Hymenoptera, Diprionidae, Diptera, Cecidomyiidae

##### Notes

oilseed rape pest host: unknown

#### Diapriidae

560AB927-579F-5FFB-B1EF-55DCA0419D5D

#### Lyteba sp. 1

0D3ECC8D-E8BC-5E03-8C69-8EF9B3753187

##### Materials

- **Type status:** Other material. **Occurrence:** individualCount: 10 males, 6 females; behavior: primary parasitoids, larval/pupal; occurrenceID: 7103B83E-8E05-5365-A4AE-3F10EDABCE1D; **Location:** country: Serbia; locality: Bajmok, Pačir, Srbobran; **Event:** samplingProtocol: Sweep net, Pan traps; eventDate: 04-07.05.2018, 07-10.05.2018, 25.05.2018, 22-24.04.2019; habitat: oilseed rape, semi-natural habitat

##### Parasite of

Diptera, Mycetophilidae, Sciaridae

##### Notes

oilseed rape pest host: unknown

#### Trichopria sp. 1

C8164C44-8CCE-5DB7-B6A5-04C578B431FD

##### Materials

- **Type status:** Other material. **Occurrence:** individualCount: 1 male; behavior: primary parasitoids, larval/pupal; occurrenceID: 08A7A3D2-D256-5D3E-810C-1369C2BE9D0A; **Location:** country: Serbia; locality: Srbobran; **Event:** samplingProtocol: Pan traps; eventDate: 24-27.04.2018; habitat: semi-natural habitat

##### Parasite of

Drosophilidae, Sarcophagidae, Sepsidae, Muscidae, Calliphoridae

##### Notes

oilseed rape pest host: unknown

#### Encyrtidae

C2FA6570-9681-5D2B-8B6C-C57BC71E55C0

#### Encyrtidae sp. 1

8A2CBCA7-EF2A-5C36-A234-311B4079E483

##### Materials

- **Type status:** Other material. **Occurrence:** individualCount: 1 male; behavior: primary/secondary parasitoids, egg/larval; occurrenceID: 665B033C-6A98-58E7-84CD-7A1EAB4A40C9; **Location:** country: Serbia; locality: Čenej; **Event:** samplingProtocol: Pan traps; eventDate: 07-10.05.2018; habitat: oilseed rape

##### Parasite of

Hemiptera, Homoptera, Coccoidea, Acarina

##### Notes

oilseed rape pest host: unknown

#### Encyrtidae sp. 2

C6D3FC59-2E1D-55A0-985E-A45A18D60E94

##### Materials

- **Type status:** Other material. **Occurrence:** individualCount: 1 female; behavior: primary/secondary parasitoids, egg/larval; occurrenceID: B44CF365-920E-5918-B950-D845517334A2; **Location:** country: Serbia; locality: Đurđin; **Event:** samplingProtocol: Pan traps; eventDate: 22-24.04.2019; habitat: semi-natural habitat

##### Parasite of

Hemiptera, Homoptera, Coccoidea, Acarina

##### Notes

oilseed rape pest host: unknown

#### Encyrtidae sp. 3

D2D5ABED-7C7D-5A7A-9EEA-807CE295B9B7

##### Materials

- **Type status:** Other material. **Occurrence:** individualCount: 2 females; behavior: primary/secondary parasitoids, egg/larval; occurrenceID: 329554B8-9214-5768-AC2A-A1C0CA3E1A72; **Location:** country: Serbia; locality: Mišićevo; **Event:** samplingProtocol: Sweep net; eventDate: 04-25-19; habitat: oilseed rape, semi-natural habitat

##### Parasite of

Hemiptera, Homoptera, Coccoidea, Acarina

##### Notes

oilseed rape pest host: unknown

#### Anagyrus sp. 1

450D52AD-5903-5E14-BF96-1248AB749650

##### Materials

- **Type status:** Other material. **Occurrence:** individualCount: 1 male, 2 females; behavior: primary parasitoids, egg; occurrenceID: EFEB1B0D-9452-5C73-960B-CEF3BB2D0609; **Location:** country: Serbia; locality: Pačir; **Event:** samplingProtocol: Pan traps; eventDate: 22-24.04.2019; habitat: semi-natural habitat

##### Parasite of

Hemiptera, Pseudococcidae?

##### Notes

oilseed rape pest host: unknown

#### Copidosoma bakeri

(Howard, 1898)

8F0C61E8-F5DA-5037-9554-A453C596185F

##### Materials

- **Type status:** Other material. **Occurrence:** individualCount: 12 males, 1 female; behavior: primary parasitoids, egg/larval; occurrenceID: A39B9228-A151-5F73-AF4B-1EEB2059E5C4; **Location:** country: Serbia; locality: Čenej, Pačir, Srbobran; **Event:** samplingProtocol: Sweep net, Pan traps; eventDate: 04-07.05.2018, 07-10.05.2018, 22-24.04.2019; habitat: oilseed rape, semi-natural habitat

##### Parasite of

Noctuidae (*Euxoaauxiliaris*)

##### Notes

oilseed rape pest host: unknown

#### Eugahania fumipennis

(Ratzeburg, 1852)

884E2226-8879-5265-9E7E-FE90D1344AC7

##### Materials

- **Type status:** Other material. **Occurrence:** individualCount: 1 male; behavior: primary parasitoids, larval; occurrenceID: EAC5B5D2-1D84-55D3-916E-4B86FBA7F964; **Location:** country: Serbia; locality: Bajmok; **Event:** samplingProtocol: Pan traps; eventDate: 22-24.04.2019; habitat: semi-natural habitat

##### Parasite of

Cicadellidae, *Macropsisvicina*

##### Notes

oilseed rape pest host: unknown

#### Metaphycus flavus

(Ashmead, 1901)

9A7CCDC2-0A45-5FCA-912F-4564EBD50FB7

##### Materials

- **Type status:** Other material. **Occurrence:** individualCount: 3 males; behavior: primary parasitoids, egg; occurrenceID: 762F7BCB-A08B-592F-943A-91081FB581A4; **Location:** country: Serbia; locality: Mišićevo, Srbobran; **Event:** samplingProtocol: Sweep net; eventDate: 07.05.2018, 25.04.2019; habitat: oilseed rape

##### Parasite of

Hemiptera, Coccoidea

##### Notes

oilseed rape pest host: unknown

#### Rhopus sp. 1

7499CE98-E742-57D8-9422-C866C75651ED

##### Materials

- **Type status:** Other material. **Occurrence:** individualCount: 1 male; behavior: primary parasitoids, egg; occurrenceID: D3D8E152-A381-585A-BFB5-C1BC53F9DE00; **Location:** country: Serbia; locality: Đurđin; **Event:** samplingProtocol: Pan traps; eventDate: 22-24.04.2019; habitat: semi-natural habitat

##### Parasite of

Hemiptera, Pseudococcidae

##### Notes

oilseed rape pest host: unknown

#### Eulophidae

532CC2C0-9C50-5832-8852-6B5E336BA9E7

#### Eulophidae sp. 1

0C614397-2612-5EA2-897A-1DBEF04A7E8A

##### Materials

- **Type status:** Other material. **Occurrence:** individualCount: 2 males; behavior: primary/secondary parasitoids, egg/larval/pupal; occurrenceID: F9DCF374-157E-5B65-BD84-865CA67D29F5; **Location:** country: Serbia; locality: Mišićevo, Pačir; **Event:** samplingProtocol: Sweep net; eventDate: 18.04.2019, 10.05.2019; habitat: oilseed rape, semi-natural habitat

##### Parasite of

Holometabolous insects

##### Notes

oilseed rape pest host: unknown

#### Eulophidae sp. 2

61EB04C5-08AF-54D2-9BDA-FFAC23A4B5E9

##### Materials

- **Type status:** Other material. **Occurrence:** individualCount: 1 male, 1 female; behavior: primary/secondary parasitoids, egg/larval/pupal; occurrenceID: A14BDA52-22D1-52AB-8ADD-D4687C566B92; **Location:** country: Serbia; locality: Čenej; **Event:** samplingProtocol: Sweep net, Pan traps; eventDate: 24-27.04.2018, 10.05.2018; habitat: oilseed rape, semi-natural habitat

##### Parasite of

Holometabolous insects

##### Notes

oilseed rape pest host: unknown

#### Eulophidae sp. 3

D538D351-98B3-53D8-A377-C948EE166CF9

##### Materials

- **Type status:** Other material. **Occurrence:** individualCount: 7 males, 3 females; behavior: primary/secondary parasitoids, egg/larval/pupal; occurrenceID: CC0F692C-2F39-5777-B056-9BED5DFA170E; **Location:** country: Serbia; locality: Čenej, Mišićevo; **Event:** samplingProtocol: Sweep net, Pan traps, Aphid colony; eventDate: 07-10.05.2018, 25.05.2018; habitat: oilseed rape, semi-natural habitat

##### Parasite of

Holometabolous insects

##### Notes

oilseed rape pest host: unknown

#### Eulophidae sp. 4

156C6425-821F-58F7-882D-D61E2FB2C441

##### Materials

- **Type status:** Other material. **Occurrence:** individualCount: 2 males; behavior: primary/secondary parasitoids, egg/larval/pupal; occurrenceID: 33B3070D-A66C-5491-9238-C41CFD109A8F; **Location:** country: Serbia; locality: Mišićevo, Srbobran; **Event:** samplingProtocol: Sweep net; eventDate: 07.05.2018, 12.06.2019; habitat: oilseed rape

##### Parasite of

Holometabolous insects

##### Notes

oilseed rape pest host: unknown

#### Eulophidae sp. 5

78048963-88B3-5839-A66A-A005881CC99E

##### Materials

- **Type status:** Other material. **Occurrence:** individualCount: 1 male, 1 female; behavior: primary/secondary parasitoids, egg/larval/pupal; occurrenceID: 5B30C53D-6E6B-52CD-B264-53B03B03E44E; **Location:** country: Serbia; locality: Bajmok; **Event:** samplingProtocol: Sweep net; eventDate: 05-08-19; habitat: oilseed rape

##### Parasite of

Holometabolous insects

##### Notes

oilseed rape pest host: unknown

#### Tetrastichinae sp. 1

DAB1CDB8-169F-502C-B7B2-D248D7FA3348

##### Materials

- **Type status:** Other material. **Occurrence:** individualCount: 15 males, 9 females; behavior: primary/secondary parasitoids, egg/larval/pupal; occurrenceID: 25F73A04-63DB-5BCC-8198-6D334C049E02; **Location:** country: Serbia; locality: Čenej, Srbobran; **Event:** samplingProtocol: Sweep net, Pan traps; eventDate: 24-27.04.2018, 04-07.05.2018, 25.05.2018; habitat: oilseed rape, semi-natural habitat

##### Parasite of

Holometabolous insects, spiders, mites, nematodes

##### Notes

oilseed rape pest host: unknown

#### Tetrastichinae sp. 2

710A6F0F-DB9B-5CC4-9334-9453C751D18D

##### Materials

- **Type status:** Other material. **Occurrence:** individualCount: 4 males; behavior: primary/secondary parasitoids, egg/larval/pupal; occurrenceID: 3B402071-358E-5202-9EE0-2FCB10998A76; **Location:** country: Serbia; locality: Čenej, Srbobran; **Event:** samplingProtocol: Sweep net, Pan traps; eventDate: 04-07.05.2018, 10.05.2018; habitat: oilseed rape

##### Parasite of

Holometabolous insects, spiders, mites, nematodes

##### Notes

oilseed rape pest host: unknown

#### Aprostocetus epicharmus

(Walker, 1839)

CED9B0F4-9AC4-58D0-920C-BF9D2352D71D

##### Materials

- **Type status:** Other material. **Occurrence:** individualCount: 2 males; behavior: primary parasitoids, larval; occurrenceID: DBE900AA-7874-5147-B369-EB8AE571ABD1; **Location:** country: Serbia; locality: Mišićevo; **Event:** samplingProtocol: Aphid colony; eventDate: 06-12-19; habitat: semi-natural habitat

##### Parasite of

*Dasineurabrassicae*

##### Notes

oilseed rape pest host: yes

#### Aprostocetus sp. 1

2D0E62B0-B4C7-5D3D-8C46-39654BB04933

##### Materials

- **Type status:** Other material. **Occurrence:** individualCount: 16 males, 1 female; behavior: primary parasitoids, larval; occurrenceID: 65C1DA0B-F6CE-5521-8C0E-697DB235729C; **Location:** country: Serbia; locality: Bajmok, Čenej, Mišićevo, Pačir, Srbobran; **Event:** samplingProtocol: Sweep net, Aphid colony; eventDate: 07.05.2018, 10.05.2018, 25.05.2018, 25.04.2019, 08.05.2019; habitat: oilseed rape, semi-natural habitat

##### Parasite of

*Dasineurabrassicae*

##### Notes

oilseed rape pest host: unknown, possible

#### Diaulinopsis arenaria

(Erdös, 1951)

D17DA956-3248-50B1-8207-CBFDE33E5E80

##### Materials

- **Type status:** Other material. **Occurrence:** individualCount: 4 males, 3 females; behavior: primary parasitoids, larval; occurrenceID: 31245DC2-09E3-5E20-AD85-28106E0475FC; **Location:** country: Serbia; locality: Čenej, Srbobran; **Event:** samplingProtocol: Sweep net, Pan traps; eventDate: 24-27.04.2018, 04-07.05.2018, 10.05.2018, 25.05.2018; habitat: oilseed rape, semi-natural habitat

##### Parasite of

*Liriomyza* spp.

##### Notes

oilseed rape pest host: unknown

#### Diglyphus aff. isaea

F2E24039-9B89-50BB-A970-A3AC31BFC566

##### Materials

- **Type status:** Other material. **Occurrence:** individualCount: 1 female; behavior: primary parasitoids, larval; occurrenceID: 77C33F6A-EF2C-5439-9474-63CAF51843D9; **Location:** country: Serbia; locality: Srbobran; **Event:** samplingProtocol: Pan traps; eventDate: 04-07.05.2018; habitat: oilseed rape

##### Parasite of

leaf miners

##### Notes

oilseed rape pest host: unknown

#### Elasmus platyedrae

Ferrière, 1935

02A077ED-72E3-5735-A273-A01C1D180F73

##### Materials

- **Type status:** Other material. **Occurrence:** individualCount: 7 males; behavior: primary/secondary parasitoids, larval; occurrenceID: 9EE8B34B-6E58-5C9F-A1CF-CAF99F0BE995; **Location:** country: Serbia; locality: Bajmok, Pačir; **Event:** samplingProtocol: Pan traps; eventDate: 17-19.04.2019, 22-24.04.2019; habitat: semi-natural habitat

##### Parasite of

Gelechiidae

##### Notes

oilseed rape pest host: unknown

#### Eulophus sp. 1

E0A023AF-D20E-5B86-AF8C-85834B63CA4E

##### Materials

- **Type status:** Other material. **Occurrence:** individualCount: 8 males, 11 females; behavior: primary parasitoids, larval; occurrenceID: 67E92477-7F66-5525-8171-B91BF18A0BEC; **Location:** country: Serbia; locality: Bajmok, Čenej, Pačir, Srbobran; **Event:** samplingProtocol: Sweep net; eventDate: 04.05.2018, 07.05.2018, 10.05.2018, 25.05.2018, 25.04.2019, 13.06.2019; habitat: oilseed rape, semi-natural habitat

##### Parasite of

Cabbage Seed Weevil, Lepidoptera

##### Notes

oilseed rape pest host: unknown, possible

#### Necremnus sp. 1

EA783903-AF01-55F7-AD39-FA7817AE7461

##### Materials

- **Type status:** Other material. **Occurrence:** individualCount: 1 female; behavior: primary parasitoids, larval; occurrenceID: 52828C98-25D5-5FAD-9603-BBEF1E42BD1D; **Location:** country: Serbia; locality: Bajmok; **Event:** samplingProtocol: Pan traps; eventDate: 17-19.04.2019; habitat: semi-natural habitat

##### Parasite of

Cabbage Seed Weevil, Lepidoptera

##### Notes

oilseed rape pest host: unknown, possible

#### Omphale clypealis

(Thomson, 1878)

4FA2EF35-A349-5B6C-A1ED-67421432B666

##### Materials

- **Type status:** Other material. **Occurrence:** individualCount: 3 males; behavior: primary parasitoids, larval; occurrenceID: 57F0A662-3BF1-5D85-B023-CCE77D6685A9; **Location:** country: Serbia; locality: Bajmok, Čenej, Pačir; **Event:** samplingProtocol: Sweep net, Pan traps; eventDate: 24-27.04.2018, 24.04.2019, 25.04.2019; habitat: oilseed rape

##### Parasite of

*Dasineurabrassicae*

##### Notes

oilseed rape pest host: yes

#### Pnigalio sp. 1

C3FF4BAB-7F15-5DE8-8217-E33A5B45F76E

##### Materials

- **Type status:** Other material. **Occurrence:** individualCount: 1 male, 2 females; behavior: primary/secondary parasitoids, larval; occurrenceID: A73F7362-9DA0-512C-98E2-BA7EF36E0272; **Location:** country: Serbia; locality: Čenej, Pačir; **Event:** samplingProtocol: Sweep net, Pan traps; eventDate: 24-27.04.2018, 25.04.2019; habitat: oilseed rape

##### Parasite of

leaf miners: Lepidoptera, Diptera, Coleoptera, Hymenoptera

##### Notes

oilseed rape pest host: unknown, possible

#### Tetrastichus sp. 1

55662491-8828-59F9-9B78-31AF4086338F

##### Materials

- **Type status:** Other material. **Occurrence:** individualCount: 9 males, 8 females; behavior: primary parasitoids, larval/pupal; occurrenceID: E1CEB768-7D5D-5081-B997-CC5BD051F615; **Location:** country: Serbia; locality: Čenej, Mišićevo, Srbobran; **Event:** samplingProtocol: Sweep net, Pan traps, Aphid colony; eventDate: 24-27.04.2018, 04-07.05.2018, 07-10.05.2018, 25.05.2018, 25.04.2019; habitat: oilseed rape, semi-natural habitat

##### Parasite of

Buprestidae, Cerambycidae, Chrysomelidae, Curculionidae, Lepidoptera, Diptera, Hymenoptera

##### Notes

oilseed rape pest host: unknown, possible

#### Eurytomidae

C160D1F1-275D-545B-BD3C-D3018F17B622

#### Eurytoma sp. 1

1BC5EF0C-8BAB-5E0B-933D-EF2D55BA8980

##### Materials

- **Type status:** Other material. **Occurrence:** individualCount: 4 males, 7 females; behavior: primary/secondary parasitoids, larval; occurrenceID: B8AA6924-7073-5B04-AFFD-EA1EA8F1323A; **Location:** country: Serbia; locality: Čenej, Mišićevo, Srbobran; **Event:** samplingProtocol: Sweep net, Pan traps, Aphid colony; eventDate: 27.04.2018, 04-07.05.2018, 07-10.05.2018, 25.05.2018, 25.04.2019; habitat: oilseed rape, semi-natural habitat

##### Parasite of

*Systole*, *Bruchophagus*, hyperparasitoid on *Tetramesa*

##### Notes

oilseed rape pest host: unknown, possible

#### Figitidae

A08CD340-38C7-5763-9684-0F688C2833A8

#### Eucolinae sp. 1

D1324F4B-9F98-5B03-BFFA-51826616FC92

##### Materials

- **Type status:** Other material. **Occurrence:** individualCount: 8 males; behavior: primary parasitoids, larval/pupal; occurrenceID: 6E9D43A2-09E0-53E1-9353-764D0E4F36C8; **Location:** country: Serbia; locality: Čenej, Đurđin, Srbobran; **Event:** samplingProtocol: Sweep net, Pan traps; eventDate: 24-27.04.2018, 07-10.05.2018, 22-24.04.2019; habitat: oilseed rape, semi-natural habitat

##### Parasite of

Cyclorraphic dipterous larvae

##### Notes

oilseed rape pest host: unknown

#### Eucolinae sp. 2

3DDF5689-EC71-5532-8001-F579D4530422

##### Materials

- **Type status:** Other material. **Occurrence:** individualCount: 2 males, 3 females; behavior: primary parasitoids, larval/pupal; occurrenceID: D2EAF200-7A66-5FD2-B360-62432C85BE8A; **Location:** country: Serbia; locality: Čenej, Srbobran; **Event:** samplingProtocol: Sweep net, Pan traps; eventDate: 24-27.04.2018, 07-10.05.2018; habitat: oilseed rape, semi-natural habitat

##### Parasite of

Cyclorraphic dipterous larvae

##### Notes

oilseed rape pest host: unknown

#### Alloxysta sp. 1

2E38F9FC-FE65-5154-9362-077D8556A179

##### Materials

- **Type status:** Other material. **Occurrence:** individualCount: 2 males; behavior: secondary parasitoids, larval; occurrenceID: E59C956B-93C4-59D4-99D5-D8A84FE5ACD3; **Location:** country: Serbia; locality: Srbobran; **Event:** samplingProtocol: Aphid colony; eventDate: 04.05.2018, 07.05.2018; habitat: oilseed rape

##### Parasite of

Aphidiinae, Aphelininae, Encyrtidae

##### Notes

oilseed rape pest host: unknown, possible

#### Rhoptomeris sp. 1

29DAE811-7EFA-50B5-BF43-B1EA450AC3AD

##### Materials

- **Type status:** Other material. **Occurrence:** individualCount: 2 males, 1 female; behavior: primary parasitoids, larval/pupal; occurrenceID: A8E2E3BE-ED68-5E53-A541-274CD00A5354; **Location:** country: Serbia; locality: Čenej, Srbobran; **Event:** samplingProtocol: Pan traps; eventDate: 24-27.04.2018; habitat: oilseed rape

##### Parasite of

Chloropidae, Diptera

##### Notes

oilseed rape pest host: unknown

#### Ichneumonidae

777FC486-109B-5DAD-818B-C696B3475240

#### Ichneumonidae sp. 1

99F78A74-6D39-5CA2-BF75-ACDFBFDCC862

##### Materials

- **Type status:** Other material. **Occurrence:** individualCount: 1 female; behavior: primary/secondary parasitoids, egg/larval; occurrenceID: C67F9BF1-DD57-5167-ACC8-733B38E5777D; **Location:** country: Serbia; locality: Srbobran; **Event:** samplingProtocol: Sweep net; eventDate: 05-07-18; habitat: oilseed rape

##### Parasite of

Holometabolous insects

##### Notes

oilseed rape pest host: unknown

#### Phaeogenini sp. 1

5C7B2E73-B110-5D6C-8009-6DA57DA8FA75

##### Materials

- **Type status:** Other material. **Occurrence:** individualCount: 1 female; behavior: primary/secondary parasitoids, larval; occurrenceID: 064DA64F-7F98-5937-982C-A00C74CC4561; **Location:** country: Serbia; locality: Srbobran; **Event:** samplingProtocol: Sweep net; eventDate: 05-25-18; habitat: oilseed rape

##### Parasite of

*Plutellaxylostella*

##### Notes

oilseed rape pest host: yes

#### Phygadeuontinae sp. 1

7BF927D2-E157-5079-8524-F42DDD2B78EE

##### Materials

- **Type status:** Other material. **Occurrence:** individualCount: 2 females; behavior: primary/secondary parasitoids, egg/larval; occurrenceID: B2778AA4-64F4-5F69-8FA4-DAD9C5EA9408; **Location:** country: Serbia; locality: Đurđin; **Event:** samplingProtocol: Pan traps; eventDate: 22-24.04.2019; habitat: semi-natural habitat

##### Parasite of

Holometabolous insects

##### Notes

oilseed rape pest host: unknown

#### Phygadeuontini sp. 1

EA1D2B2F-51D1-52D5-9A43-62D90EAABCFE

##### Materials

- **Type status:** Other material. **Occurrence:** individualCount: 1 female; behavior: primary/secondary parasitoids, larval; occurrenceID: 28B6A6C4-A371-5989-9208-88674A1797FE; **Location:** country: Serbia; locality: Srbobran; **Event:** samplingProtocol: Sweep net; eventDate: 05-10-18; habitat: oilseed rape

##### Parasite of

Symphyta

##### Notes

oilseed rape pest host: unknown

#### Aneuclis incidens

(Thomson, 1889)

23EDEEBC-382B-562D-9565-A558FEFDB9E0

##### Materials

- **Type status:** Other material. **Occurrence:** individualCount: 1 male, 2 females; behavior: primary parasitoids, larval; occurrenceID: 183E1573-8527-5A4C-AD8B-C6D6D4B465BB; **Location:** country: Serbia; locality: Čenej, Pačir, Srbobran; **Event:** samplingProtocol: Sweep net, Pan traps; eventDate: 24-27.04.2018, 10.05.2019; habitat: oilseed rape, semi-natural habitat

##### Parasite of

*Meligethesaeneus*

##### Notes

oilseed rape pest host: yes

#### Aptesis flagitator

(Rossi, 1794)

FD4BB7BE-70D6-58FC-92EE-41274B2B174F

##### Materials

- **Type status:** Other material. **Occurrence:** individualCount: 1 male; behavior: primary parasitoids, larval; occurrenceID: C2101708-61BC-55AA-8D4F-478F8D7DDA85; **Location:** country: Serbia; locality: Bajmok; **Event:** samplingProtocol: Sweep net; eventDate: 05-08-19; habitat: oilseed rape

##### Parasite of

*Agonopterixheracliana*, *Athaliaspinarum*

##### Notes

oilseed rape pest host: yes

#### Bathyplectes curculionis

(Thomson, 1887)

4F1CE16B-D879-5CC4-8042-686F50218AA2

##### Materials

- **Type status:** Other material. **Occurrence:** individualCount: 1 female; behavior: primary parasitoids, pupal; occurrenceID: AC0A6F81-CAE3-5C1C-BD6E-45A10A1B37D1; **Location:** country: Serbia; locality: Srbobran; **Event:** samplingProtocol: Sweep net; eventDate: 04-27-18; habitat: semi-natural habitat

##### Parasite of

*Apionpisi*, *Hypera* spp.

##### Notes

oilseed rape pest host: unknown

#### Collyria coxator

(Villers, 1789)

A9BA8543-3302-53B2-AE02-3C174BBBE02B

##### Materials

- **Type status:** Other material. **Occurrence:** individualCount: 7 males, 14 females; behavior: primary parasitoids, larval; occurrenceID: CC1A9ECA-A7E3-52A2-9004-232735782E0D; **Location:** country: Serbia; locality: Bajmok, Čenej, Đurđin, Pačir, Srbobran; **Event:** samplingProtocol: Sweep net, Pan traps; eventDate: 04-07.05.2018, 10.05.2018, 25.05.2018, 22-24.04.2019, 08.05.2019; habitat: oilseed rape, semi-natural habitat

##### Parasite of

*Cephuscinctus*, *Cephuspygmeus*

##### Notes

oilseed rape pest host: unknown

#### Diadegma insulare

(Cresson, 1865)

910A5972-D254-5F43-9031-F5B14AC28AE5

##### Materials

- **Type status:** Other material. **Occurrence:** individualCount: 1 male; behavior: primary parasitoids, pupal; occurrenceID: CD57A189-7AE6-5C7A-B431-CBA52EAE2F73; **Location:** country: Serbia; locality: Mišićevo; **Event:** samplingProtocol: Sweep net; eventDate: 06-12-19; habitat: oilseed rape

##### Parasite of

*Plutellaxylostella*

##### Notes

oilseed rape pest host: yes

#### Diplazon laetatorius

(Fabricius, 1781)

CE3BC717-5DB5-58C7-9850-022E67B19252

##### Materials

- **Type status:** Other material. **Occurrence:** individualCount: 2 males; behavior: primary parasitoids, pupal; occurrenceID: 7FC941A6-E807-5486-B88F-9347CAAC84C4; **Location:** country: Serbia; locality: Čenej, Mišićevo; **Event:** samplingProtocol: Sweep net, Aphid colony; eventDate: 27.04.2018, 12.06.2019; habitat: oilseed rape, semi-natural habitat

##### Parasite of

Diptera, Syrphidae

##### Notes

oilseed rape pest host: unknown

#### Dusona pugillator

(Linnaeus, 1758)

EBF44B84-4AED-56B2-8551-929EEEE60960

##### Materials

- **Type status:** Other material. **Occurrence:** individualCount: 2 males; behavior: primary parasitoids, larval; occurrenceID: D3D73282-6F08-588D-B352-81F81E9CF17F; **Location:** country: Serbia; locality: Bajmok, Pačir; **Event:** samplingProtocol: Sweep net, Pan traps; eventDate: 22-24.04.2019, 10.05.2019; habitat: semi-natural habitat

##### Parasite of

Lepidoptera

##### Notes

oilseed rape pest host: unknown

#### Diphyus ochromelas

(Gmelin, 1790)

25ABD348-935F-5393-BC08-1115F9438AC2

##### Materials

- **Type status:** Other material. **Occurrence:** individualCount: 1 male; behavior: primary parasitoids, larval; occurrenceID: AD397A1A-B727-5C1F-BC52-DF4C511895AB; **Location:** country: Serbia; locality: Bajmok; **Event:** samplingProtocol: Sweep net; eventDate: 05-08-19; habitat: oilseed rape

##### Parasite of

Lepidoptera

##### Notes

oilseed rape pest host: unknown

#### Mesochorus sp. 1

B29F49CB-7420-54C8-9A34-1C785FF25A2A

##### Materials

- **Type status:** Other material. **Occurrence:** individualCount: 1 male; behavior: secondary parasitoids, larval; occurrenceID: 747E1AF2-EA36-5BE5-B065-72D08031D9F9; **Location:** country: Serbia; locality: Čenej; **Event:** samplingProtocol: Sweep net; eventDate: 05-10-18; habitat: semi-natural habitat

##### Parasite of

*Cotesia* spp.

##### Notes

oilseed rape pest host: unknown, possible

#### Olesicampe sp. 1

66557875-969D-5A59-9D50-8AF7F5202DB1

##### Materials

- **Type status:** Other material. **Occurrence:** individualCount: 1 female; behavior: primary parasitoids, pupal; occurrenceID: 6489C6DD-884E-5CE6-9C8E-D4FA7EF45758; **Location:** country: Serbia; locality: Srbobran; **Event:** samplingProtocol: Pan traps; eventDate: 04-07.05.2018; habitat: oilseed rape

##### Parasite of

Tenthredinidae

##### Notes

oilseed rape pest host: unknown

#### Stibeutes curvispina

(Thomson, 1884)

5724EBBE-575F-5BBA-997F-BCE10209FAFB

##### Materials

- **Type status:** Other material. **Occurrence:** individualCount: 3 males, 2 females; behavior: primary parasitoids, larval; occurrenceID: 12C812FB-44D7-54FE-A5BC-CFEA81EDF1E1; **Location:** country: Serbia; locality: Čenej, Đurđin, Srbobran; **Event:** samplingProtocol: Sweep net, Pan traps; eventDate: 04-07.05.2018, 07-10.05.2018, 22-24.04.2019; habitat: oilseed rape

##### Parasite of

*Ceutorhynchuspallidactylus*

##### Notes

oilseed rape pest host: yes

#### Syrphophilus bizonarius

(Gravenhorst, 1829)

5F8C2C76-A644-5936-9DB3-C6DA3C2D5D4F

##### Materials

- **Type status:** Other material. **Occurrence:** individualCount: 1 female; behavior: primary parasitoids, larval; occurrenceID: C55EB950-248B-5402-B80F-E6B92AD77282; **Location:** country: Serbia; locality: Pačir; **Event:** samplingProtocol: Sweep net; eventDate: 05-10-19; habitat: semi-natural habitat

##### Parasite of

*Atherigonasoccata*, *Deliaradicum*, *Episyrphusbalteatus*, *Eupeodescorollae*, *Eupeodesluniger*, *Emexspinosa*, *Loxostegesticticalis*, *Neocnemodonvitripennis*, *Sphaerophoriascripta*

##### Notes

oilseed rape pest host: unknown

#### Tersilochus heterocerus

(Thomson, 1889)

56FF8753-928A-554F-A418-69B82B37A8E5

##### Materials

- **Type status:** Other material. **Occurrence:** individualCount: 11 males, 7 females; behavior: primary parasitoids, larval; occurrenceID: FD6E7063-B95F-58FC-B7FB-D0FF05686E8E; **Location:** country: Serbia; locality: Čenej, Srbobran; **Event:** samplingProtocol: Sweep net; eventDate: 04.05.2018, 07.05.2018, 10.05.2018, 27.04.2018; habitat: oilseed rape, semi-natural habitat

##### Parasite of

*Meligethesaeneus*

##### Notes

oilseed rape pest host: yes

#### Thrybius praedator

(Rossi, 1792)

6309EFB6-54E8-58D4-AA03-A111D92186BB

##### Materials

- **Type status:** Other material. **Occurrence:** individualCount: 1 female; behavior: primary parasitoids, larval; occurrenceID: E1618D03-CD78-5960-811F-FF74A0DC5B74; **Location:** country: Serbia; locality: Srbobran; **Event:** samplingProtocol: Sweep net; eventDate: 05-04-18; habitat: semi-natural habitat

##### Parasite of

*Achnaraspargani*, *Chilophragmitellus*, *Obereaeuphorbiae*

##### Notes

oilseed rape pest host: unknown

#### Megaspilidae

969903C5-CC72-5D2E-A782-1471AD9025E3

#### Conostigmus rufescens

Kieffer, 1907

9E52FC2D-F8C0-5458-87C3-0BAEE1387106

##### Materials

- **Type status:** Other material. **Occurrence:** individualCount: 1 male; behavior: primary parasitoids, egg/larval; occurrenceID: FEF6BD20-000B-5AB3-A033-AB1C56E92AE1; **Location:** country: Serbia; locality: Srbobran; **Event:** samplingProtocol: Sweep net; eventDate: 05-07-18; habitat: oilseed rape

##### Parasite of

*Dasineurabrassicae*

##### Notes

oilseed rape pest host: yes

#### Lagynodes pallidus

(Boheman 1832)

0AB68793-260B-5E68-BBCE-374FBA6C22C5

##### Materials

- **Type status:** Other material. **Occurrence:** individualCount: 9 males, 6 females; behavior: secondary parasitoids, larval; occurrenceID: 6CEF44D2-BB26-55B3-8C3B-AAF783C929E2; **Location:** country: Serbia; locality: Čenej, Srbobran; **Event:** samplingProtocol: Sweep net, Pan traps; eventDate: 24-27.04.2018, 04-07.05.2018, 07-10.05.2018; habitat: oilseed rape, semi-natural habitat

##### Parasite of

*Cotesia* spp.

##### Notes

oilseed rape pest host: unknown

#### Mymaridae

E375A5D5-05AF-5A37-8338-357E53213C9D

#### Mymaridae sp. 1

F2636BD1-1E15-505E-ADC3-5C1FF3C83527

##### Materials

- **Type status:** Other material. **Occurrence:** individualCount: 1 male; behavior: primary parasitoids, egg; occurrenceID: 84DD4EAA-01BE-5B28-8A93-77EBB1BF35DF; **Location:** country: Serbia; locality: Srbobran; **Event:** samplingProtocol: Pan traps; eventDate: 24-27.04.2018; habitat: semi-natural habitat

##### Parasite of

Auchenorrhynchous, Hemiptera, Coleoptera, Psocoptera

##### Notes

oilseed rape pest host: unknown

#### Anagrus sp. 1

6E59C0C8-8442-505A-89BE-AFA5E101A8D1

##### Materials

- **Type status:** Other material. **Occurrence:** individualCount: 3 males, 1 female; behavior: primary parasitoids, egg; occurrenceID: 0DAC860B-40C2-503B-BBBE-2E2E7A426D30; **Location:** country: Serbia; locality: Bajmok, Đurđin, Mišićevo; **Event:** samplingProtocol: Sweep net, Pan traps; eventDate: 20-22.04.2019, 24.04.2019, 25.04.2019; habitat: oilseed rape, semi-natural habitat

##### Parasite of

Cicadellidae

##### Notes

oilseed rape pest host: unknown

#### Anagrus sp. 2

7062DAF5-189A-515F-A393-73548617C29C

##### Materials

- **Type status:** Other material. **Occurrence:** individualCount: 2 males; behavior: primary parasitoids, egg; occurrenceID: 97C28639-6481-501E-A8A1-08EE18C9F29A; **Location:** country: Serbia; locality: Čenej, Srbobran; **Event:** samplingProtocol: Pan traps; eventDate: 04-07.05.2018; habitat: oilseed rape, semi-natural habitat

##### Parasite of

Cicadellidae

##### Notes

oilseed rape pest host: unknown

#### Anagrus sp. 3

DD803332-9873-5D88-85D7-21AD7FD579B8

##### Materials

- **Type status:** Other material. **Occurrence:** individualCount: 2 males; behavior: primary parasitoids, egg; occurrenceID: 2C0C5CF7-B1AD-502E-9B1F-61E21AE752D3; **Location:** country: Serbia; locality: Mišićevo; **Event:** samplingProtocol: Sweep net; eventDate: 25.04.2019, 12.06.2019; habitat: oilseed rape

##### Parasite of

Cicadellidae

##### Notes

oilseed rape pest host: unknown

#### Anaphes sp. 1

6D2C5578-0942-5F7C-A776-9C43483C2F5C

##### Materials

- **Type status:** Other material. **Occurrence:** individualCount: 27 males, 6 females; behavior: primary parasitoids, egg; occurrenceID: 9047154B-F856-51C1-AB08-FEB894164FCC; **Location:** country: Serbia; locality: Bajmok, Čenej, Đurđin, Mišićevo, Pačir, Srbobran; **Event:** samplingProtocol: Sweep net, Pan traps; eventDate: 24-27.04.2018, 04-07.05.2018, 10.05.2018, 22-24.04.2019, 25.04.2019; habitat: oilseed rape, semi-natural habitat

##### Parasite of

Coleoptera, Curculionidae, Chrysomelidae, Hemiptera, Miridae

##### Notes

oilseed rape pest host: unknown, possible

#### Gonatocerus sp. 1

85DC4C9E-1335-5A6C-A74B-BB8BDB92BACA

##### Materials

- **Type status:** Other material. **Occurrence:** individualCount: 1 male, 1 female; behavior: primary parasitoids, egg; occurrenceID: B9F3DFBB-3D89-5BF3-BA8E-F5A92F59E93C; **Location:** country: Serbia; locality: Bajmok; **Event:** samplingProtocol: Sweep net; eventDate: 05-08-19; habitat: oilseed rape

##### Parasite of

Cicadellidae

##### Notes

oilseed rape pest host: unknown

#### Litus cynipseus

Haliday, 1833

02F9733A-3ED4-5D97-BDDF-AC501CAB0BC9

##### Materials

- **Type status:** Other material. **Occurrence:** individualCount: 2 males; behavior: primary parasitoids, egg; occurrenceID: 5F95AF60-AC55-546E-A606-10F10185B721; **Location:** country: Serbia; locality: Čenej, Srbobran; **Event:** samplingProtocol: Sweep net, Pan traps; eventDate: 04-07.05.2018, 10.05.2018; habitat: oilseed rape, semi-natural habitat

##### Parasite of

Coleoptera, Staphylinidae

##### Notes

oilseed rape pest host: unknown

#### Lymaenon sp. 1

A5D24E73-33DF-5FC9-AFCF-DE3E681C477B

##### Materials

- **Type status:** Other material. **Occurrence:** individualCount: 1 male, 1 female; behavior: primary parasitoids, egg; occurrenceID: 7E2F864C-48F5-5C7F-B559-119DB45F5F9E; **Location:** country: Serbia; locality: Srbobran; **Event:** samplingProtocol: Sweep net; eventDate: 05-04-18; habitat: oilseed rape

##### Parasite of

Cicadellidae, Membracoidea

##### Notes

oilseed rape pest host: unknown

#### Ooctonus sp. 1

1DB5FBA3-A8C7-50C4-AC43-45BAA94CAC4B

##### Materials

- **Type status:** Other material. **Occurrence:** individualCount: 1 male, 2 females; behavior: primary parasitoids, egg; occurrenceID: C1AD470E-FA4D-563F-AAAD-C752EA57D667; **Location:** country: Serbia; locality: Čenej, Srbobran; **Event:** samplingProtocol: Sweep net, Pan traps; eventDate: 04-07.05.2018, 07-10.05.2018; habitat: oilseed rape, semi-natural habitat

##### Parasite of

Cercopoidea, Cicadellidae

##### Notes

oilseed rape pest host: unknown

#### Ooctonus vulgatus

Haliday, 1833

69FAC725-8D44-549B-81D6-05D999DD6F77

##### Materials

- **Type status:** Other material. **Occurrence:** individualCount: 1 male; behavior: primary parasitoids, egg; occurrenceID: 3411EABA-B0DF-5C42-AF8D-86EE173E223D; **Location:** country: Serbia; locality: Čenej; **Event:** samplingProtocol: Pan traps; eventDate: 04-07.05.2018; habitat: oilseed rape

##### Parasite of

*Philaenusleucophthalmus*, *Philaenusspumarius*

##### Notes

oilseed rape pest host: unknown

#### Polynema sp. 1

57B97676-90DF-5093-8081-45E05F0EE168

##### Materials

- **Type status:** Other material. **Occurrence:** individualCount: 1 male; behavior: primary parasitoids, egg; occurrenceID: DF42C77F-441F-58DF-8209-E0D66D088FA6; **Location:** country: Serbia; locality: Čenej; **Event:** samplingProtocol: Pan traps; eventDate: 24-27.04.2018; habitat: oilseed rape

##### Parasite of

Cicadellidae

##### Notes

oilseed rape pest host: unknown

#### Perilampidae

89743F7E-6233-5F64-A49E-07684A9F2CEB

#### Perilampidae sp. 1

72C57D01-6D88-562D-9A9B-93EE5545232C

##### Materials

- **Type status:** Other material. **Occurrence:** individualCount: 1 male; behavior: primary/secondary parasitoids; occurrenceID: 7DAD5AEB-28D5-587B-949F-16730565D0F7; **Location:** country: Serbia; locality: Bajmok; **Event:** samplingProtocol: Sweep net; eventDate: 06-13-19; habitat: oilseed rape

##### Parasite of

Hymenoptera, Diptera, Coleoptera, Lepidoptera, Neuroptera

##### Notes

oilseed rape pest host: unknown

#### Chrysolampus thenae

(Walker, 1848)

02CF2E96-2861-53D6-B35B-AEDAD924EB63

##### Materials

- **Type status:** Other material. **Occurrence:** individualCount: 17 males, 12 females; behavior: primary parasitoids, larval/pupal; occurrenceID: F85D3D08-C1FE-52C7-B833-46B497D6904E; **Location:** country: Serbia; locality: Čenej, Srbobran; **Event:** samplingProtocol: Sweep net; eventDate: 27.04.2018, 04.05.2018, 07.05.2018, 10.05.2018; habitat: oilseed rape, semi-natural habitat

##### Parasite of

*Meligethespedicularis*

##### Notes

oilseed rape pest host: unknown

#### Perilampus aeneus

(Rossius, 1790)

CFFEA244-A636-5FB0-9846-47DF315477E1

##### Materials

- **Type status:** Other material. **Occurrence:** individualCount: 1 female; behavior: primary parasitoids, larval; occurrenceID: BA6E5516-6F02-56B5-95EB-05FCDCE6A515; **Location:** country: Serbia; locality: Bajmok; **Event:** samplingProtocol: Sweep net; eventDate: 06-13-19; habitat: oilseed rape

##### Parasite of

*Athaliarosae*

##### Notes

oilseed rape pest host: yes

#### Pirenidae

CBBD1B46-56F3-5D93-9B2E-BE10D5E2B042

#### Macroglenes sp. 1

E97B45BB-97D3-5DD4-A340-97F1222AB120

##### Materials

- **Type status:** Other material. **Occurrence:** individualCount: 1 female; behavior: primary parasitoids, egg/larval; occurrenceID: 64D88E4B-98DF-505A-8F73-991A3307575B; **Location:** country: Serbia; locality: Čenej; **Event:** samplingProtocol: Pan traps; eventDate: 04-07.05.2018; habitat: oilseed rape

##### Parasite of

Cecidomyiidae

##### Notes

oilseed rape pest host: unknown

#### Platygastridae

5FB6009C-8811-53CE-9E31-AB882DA162D8

#### Euxestonotus error

(Fitch, 1861)

B20446F5-402D-5FAD-BE92-A82BCBFE2616

##### Materials

- **Type status:** Other material. **Occurrence:** individualCount: 2 males; behavior: primary parasitoids, larval; occurrenceID: D2051C6B-CCEF-5BD6-951B-4A4919461A19; **Location:** country: Serbia; locality: Čenej, Mišićevo; **Event:** samplingProtocol: Oilseed pods, Pan traps; eventDate: 04-07.05.2018, 12.06.2019; habitat: oilseed rape

##### Parasite of

*Sitodiplosismosellana*, *Dasineurabrassicae*?

##### Notes

oilseed rape pest host: unknown

#### Inostemma boscii

(Jurine, 1807)

D900FD15-BC87-5A49-B46A-CC4A31C04B52

##### Materials

- **Type status:** Other material. **Occurrence:** individualCount: 3 males; behavior: primary parasitoids, egg/larval; occurrenceID: 679DEE51-0DD2-51E1-BD76-D94776FDB43E; **Location:** country: Serbia; locality: Čenej, Mišićevo; **Event:** samplingProtocol: Sweep net, Pan traps, Aphid colony; eventDate: 07-10.05.2018, 12.06.2019; habitat: oilseed rape, semi-natural habitat

##### Parasite of

*Dasineurabrassicae*

##### Notes

oilseed rape pest host: yes

#### Platygaster sp. 1

206B3814-EDCB-5C63-9730-7CF6208992D3

##### Materials

- **Type status:** Other material. **Occurrence:** individualCount: 1 male; behavior: primary parasitoids, larval; occurrenceID: CE7D884E-5A2C-5411-BEAF-3B9BC5B0E1B2; **Location:** country: Serbia; locality: Mišićevo; **Event:** samplingProtocol: Sweep net; eventDate: 04-25-19; habitat: semi-natural habitat

##### Parasite of

Cecidomyiidae (*Dasineurabrassicae*?)

##### Notes

oilseed rape pest host: unknown, possible

#### Platygaster sp. 2

9959099C-4DDD-543A-A305-BC3C43B1AE57

##### Materials

- **Type status:** Other material. **Occurrence:** individualCount: 5 males, 1 female; behavior: primary parasitoids, larval; occurrenceID: 518A0586-0A1A-59F0-8C02-16CEDB1BED18; **Location:** country: Serbia; locality: Bajmok, Đurđin, Srbobran; **Event:** samplingProtocol: Sweep net, Pan traps; eventDate: 07.05.2018, 17-19.04.2019, 22-24.04.2019; habitat: oilseed rape, semi-natural habitat

##### Parasite of

Cecidomyiidae (*Dasineurabrassicae*?)

##### Notes

oilseed rape pest host: unknown, possible

#### Platygaster subuliformis

Kieffer, 1926

7AD545D2-100E-597C-9D9A-B676A42ED3E3

##### Materials

- **Type status:** Other material. **Occurrence:** individualCount: 1 male; behavior: primary parasitoids, egg/larval; occurrenceID: 4D15DF4D-D4AF-522F-8D92-C9DCC1A223F2; **Location:** country: Serbia; locality: Čenej; **Event:** samplingProtocol: Pan traps; eventDate: 07-10.05.2018; habitat: oilseed rape

##### Parasite of

*Dasineurabrassicae*

##### Notes

oilseed rape pest host: yes

#### Synopeas sp. 1

9AF8645D-366D-5964-BD75-E7835921F0F7

##### Materials

- **Type status:** Other material. **Occurrence:** individualCount: 1 male; behavior: primary parasitoids, egg/larval; occurrenceID: 290E0998-E75F-52A9-9155-ADFAB09FA382; **Location:** country: Serbia; locality: Mišićevo; **Event:** samplingProtocol: Sweep net; eventDate: 04-25-19; habitat: semi-natural habitat

##### Parasite of

Cecidomyiidae (*Dasineurabrassicae*?)

##### Notes

oilseed rape pest host: unknown, possible

#### Telenomus sp. 1

A5109BE9-F490-534D-9F8C-0514E295FDF8

##### Materials

- **Type status:** Other material. **Occurrence:** individualCount: 25 males, 6 females; behavior: primary parasitoids, egg; occurrenceID: 8A98FE66-1FF3-5068-B91F-7CE2629032F3; **Location:** country: Serbia; locality: Bajmok, Čenej, Đurđin, Mišićevo, Srbobran; **Event:** samplingProtocol: Sweep net, Pan traps; eventDate: 24-27.04.2018, 04-07.05.2018, 07-10.05.2018, 22-24.04.2019, 25.04.2019, 12.06.2019, 13.06.2019; habitat: oilseed rape, semi-natural habitat

##### Parasite of

Lepidoptera, Heteroptera, Diptera, Neuroptera

##### Notes

oilseed rape pest host: unknown

#### Telenomus sp. 2

DE64E44C-E8BF-595B-BA10-FC73521C8596

##### Materials

- **Type status:** Other material. **Occurrence:** individualCount: 5 males, 3 females; behavior: primary parasitoids, egg; occurrenceID: 3FF5D53B-3C0F-5687-8C81-91E1203DE58E; **Location:** country: Serbia; locality: Đurđin, Srbobran; **Event:** samplingProtocol: Pan traps; eventDate: 24-27.04.2018, 04-07.05.2018, 07-10.05.2018, 22-24.04.2019; habitat: oilseed rape, semi-natural habitat

##### Parasite of

Lepidoptera, Heteroptera, Diptera, Neuroptera

##### Notes

oilseed rape pest host: unknown

#### Telenomus sp. 3

AC63F149-D78D-562D-A0C7-A68D85D9720F

##### Materials

- **Type status:** Other material. **Occurrence:** individualCount: 17 males, 2 females; behavior: primary parasitoids, egg; occurrenceID: F2466748-82B0-547A-B168-23D648548A6E; **Location:** country: Serbia; locality: Bajmok, Čenej, Đurđin, Mišićevo, Pačir, Srbobran; **Event:** samplingProtocol: Sweep net, Pan traps; eventDate: 24-27.04.2018, 04-07.05.2018, 07-10.05.2018, 22-24.04.2019, 25.04.2019, 13.06.2019; habitat: oilseed rape, semi-natural habitat

##### Parasite of

Lepidoptera, Heteroptera, Diptera, Neuroptera

##### Notes

oilseed rape pest host: unknown

#### Telenomus sp. 4

232ED363-C3CF-5ADE-A9A7-4D331597192B

##### Materials

- **Type status:** Other material. **Occurrence:** individualCount: 15 males, 6 females; behavior: primary parasitoids, egg; occurrenceID: 85A0CC52-0238-5F96-B65D-D7F0887B1630; **Location:** country: Serbia; locality: Bajmok, Čenej, Srbobran; **Event:** samplingProtocol: Sweep net, Pan traps; eventDate: 24-27.04.2018, 04-07.05.2018, 07-10.05.2018, 13.06.2019; habitat: oilseed rape, semi-natural habitat

##### Parasite of

Lepidoptera, Heteroptera, Diptera, Neuroptera

##### Notes

oilseed rape pest host: unknown

#### Pteromalidae

DCF73CD2-81E6-5AF2-A409-63B9280897F3

#### Pteromalidae sp. 1

2BA65EB3-6345-5733-8C78-D5B38E644332

##### Materials

- **Type status:** Other material. **Occurrence:** individualCount: 4 males; behavior: primary/secondary parasitoids, larval/pupal; occurrenceID: 3AFD2F76-5B82-5461-9D51-BB9993A1B4AF; **Location:** country: Serbia; locality: Čenej, Srbobran; **Event:** samplingProtocol: Sweep net, Pan traps; eventDate: 27.04.2018, 04-07.05.2018; habitat: oilseed rape

##### Parasite of

Lepidoptera, Coleoptera, Diptera

##### Notes

oilseed rape pest host: unknown

#### Pteromalidae sp. 2

CCBA2AD4-35FF-59CE-9215-88BB63430F18

##### Materials

- **Type status:** Other material. **Occurrence:** individualCount: 1 male; behavior: primary/secondary parasitoids, larval/pupal; occurrenceID: 9C1CFA2D-48B0-5510-9CE6-46D7A0C71457; **Location:** country: Serbia; locality: Čenej; **Event:** samplingProtocol: Pan traps; eventDate: 24-27.04.2018; habitat: oilseed rape

##### Parasite of

Lepidoptera, Coleoptera, Diptera

##### Notes

oilseed rape pest host: unknown

#### Pteromalidae sp. 3

AE97C196-E294-500D-9857-D2A46699A5E0

##### Materials

- **Type status:** Other material. **Occurrence:** individualCount: 1 male; behavior: primary/secondary parasitoids, larval/pupal; occurrenceID: 6FF49984-DB97-5383-8383-99B291993177; **Location:** country: Serbia; locality: Srbobran; **Event:** samplingProtocol: Pan traps; eventDate: 04-07.05.2018; habitat: semi-natural habitat

##### Parasite of

Lepidoptera, Coleoptera, Diptera

##### Notes

oilseed rape pest host: unknown

#### Pteromalidae sp. 4

78052078-E43E-5CD6-8438-3A3D43B1E0B2

##### Materials

- **Type status:** Other material. **Occurrence:** individualCount: 2 males; behavior: primary/secondary parasitoids, larval/pupal; occurrenceID: 0179DAD3-C720-5A57-AEAC-723A1C9ADB93; **Location:** country: Serbia; locality: Čenej, Srbobran; **Event:** samplingProtocol: Sweep net; eventDate: 04.05.2018, 07.05.2018; habitat: oilseed rape

##### Parasite of

Lepidoptera, Coleoptera, Diptera

##### Notes

oilseed rape pest host: unknown

#### Pteromalidae sp. 5

1E26305B-9E58-54FC-8C37-4525BFDB7832

##### Materials

- **Type status:** Other material. **Occurrence:** individualCount: 8 males; behavior: primary/secondary parasitoids, larval/pupal; occurrenceID: 7B2133A9-66DC-583C-B802-5C239B0A4033; **Location:** country: Serbia; locality: Čenej, Đurđin, Pačir, Srbobran; **Event:** samplingProtocol: Sweep net, Pan traps; eventDate: 04-07.05.2018, 25.04.2019, 08.05.2019; habitat: oilseed rape

##### Parasite of

Lepidoptera, Coleoptera, Diptera

##### Notes

oilseed rape pest host: unknown

#### Pteromalidae sp. 6

E21FD9CE-B5C1-5FD3-99FD-F7E84911AF09

##### Materials

- **Type status:** Other material. **Occurrence:** individualCount: 3 males; behavior: primary/secondary parasitoids, larval/pupal; occurrenceID: F3CE52EF-8D40-5F57-8755-722D2E3E1A4C; **Location:** country: Serbia; locality: Čenej; **Event:** samplingProtocol: Sweep net; eventDate: 27.04.2018, 04.05.2018, 07.05.2018; habitat: oilseed rape

##### Parasite of

Lepidoptera, Coleoptera, Diptera

##### Notes

oilseed rape pest host: unknown

#### Pteromalidae sp. 7

08F703B6-ECCB-5941-BE60-8A932D403EC4

##### Materials

- **Type status:** Other material. **Occurrence:** individualCount: 5 males, 2 females; behavior: primary/secondary parasitoids, larval/pupal; occurrenceID: D1A62987-9E64-53EE-A8CC-F58803407DFD; **Location:** country: Serbia; locality: Čenej, Mišićevo, Srbobran; **Event:** samplingProtocol: Sweep net; eventDate: 27.04.2018, 10.05.2018, 25.05.2018; habitat: oilseed rape, semi-natural habitat

##### Parasite of

Lepidoptera, Coleoptera, Diptera

##### Notes

oilseed rape pest host: unknown

#### Pteromalidae sp. 8

C309E80E-14C7-5765-926C-3B5D006C5B6D

##### Materials

- **Type status:** Other material. **Occurrence:** individualCount: 4 males, 2 females; behavior: primary/secondary parasitoids, larval/pupal; occurrenceID: 7E4CFEEE-8C69-5CBC-B5B5-63E2603EB967; **Location:** country: Serbia; locality: Bajmok, Čenej, Srbobran; **Event:** samplingProtocol: Sweep net; eventDate: 04.05.2018, 10.05.2018, 24.04.2019; habitat: oilseed rape

##### Parasite of

Lepidoptera, Coleoptera, Diptera

##### Notes

oilseed rape pest host: unknown

#### Pteromalidae sp. 9

901DC59D-7165-5236-8587-103B374DAFE5

##### Materials

- **Type status:** Other material. **Occurrence:** individualCount: 1 male, 1 female; behavior: primary/secondary parasitoids, larval/pupal; occurrenceID: 3F827E96-D1E1-5159-AB7A-167D27E787FE; **Location:** country: Serbia; locality: Čenej; **Event:** samplingProtocol: Sweep net; eventDate: 27.04.2018, 10.05.2018; habitat: oilseed rape, semi-natural habitat

##### Parasite of

Lepidoptera, Coleoptera, Diptera

##### Notes

oilseed rape pest host: unknown

#### Pteromalidae sp. 10

B9AB273B-BFB7-5D9D-994C-C0C257EF33B2

##### Materials

- **Type status:** Other material. **Occurrence:** individualCount: 1 male; behavior: primary/secondary parasitoids, larval/pupal; occurrenceID: DE482C0B-356E-597F-9158-22C2E8DA290F; **Location:** country: Serbia; locality: Srbobran; **Event:** samplingProtocol: Aphid colony; eventDate: 05-07-18; habitat: oilseed rape

##### Parasite of

Lepidoptera, Coleoptera, Diptera

##### Notes

oilseed rape pest host: unknown

#### Pteromalidae sp. 11

CB1E0BE0-A69E-5E9C-B1E7-B4856CABC4D0

##### Materials

- **Type status:** Other material. **Occurrence:** individualCount: 1 female; behavior: primary/secondary parasitoids, larval/pupal; occurrenceID: 4FBAA759-55F7-51FB-AA60-7D18FFC729CC; **Location:** country: Serbia; locality: Bajmok; **Event:** samplingProtocol: Pan traps; eventDate: 17-19.04.2019; habitat: semi-natural habitat

##### Parasite of

Lepidoptera, Coleoptera, Diptera

##### Notes

oilseed rape pest host: unknown

#### Dibrachys microgastri

(Bouché, 1834)

2FE72C88-6208-53F4-BCE1-153841352AF7

##### Materials

- **Type status:** Other material. **Occurrence:** individualCount: 1 male; behavior: secondary parasitoids, pupal; occurrenceID: C2BE8652-2DBC-5A8D-9A41-B2A6C789A542; **Location:** country: Serbia; locality: Srbobran; **Event:** samplingProtocol: Pan traps; eventDate: 24-27.04.2018; habitat: oilseed rape

##### Parasite of

*Cotesia* spp.

##### Notes

oilseed rape pest host: unknown

#### Mesopolobus incultus

(Walker, 1834)

5B9FB149-D573-5591-88C5-80C4E5F2E25B

##### Materials

- **Type status:** Other material. **Occurrence:** individualCount: 4 males, 1 female; behavior: primary parasitoids, larval; occurrenceID: 4FB3965B-1340-5F9B-BB35-111CAE2B6B29; **Location:** country: Serbia; locality: Bajmok, Mišićevo, Pačir; **Event:** samplingProtocol: Sweep net; eventDate: 25.04.2019, 12.06.2019, 13.06.2019; habitat: oilseed rape

##### Parasite of

Curculionidae, *Gymnetron* sp., *Gymnetronpascuorum*, *Mecinus* sp., Scolytidae, *Polygraphuspoligraphus*, Agromyzidae, *Phytobiahumeralis*, Cecidomyiidae, *Kaltenbachiolastrobi*

##### Notes

oilseed rape pest host: unknown

#### Mesopolobus morys

(Walker, 1848)

EBBA4A47-9D98-5B4F-B52F-2B2A55704D13

##### Materials

- **Type status:** Other material. **Occurrence:** individualCount: 23 males, 5 females; behavior: primary parasitoids, larval; occurrenceID: 009636D4-DFBA-522C-90B6-8DBA0B11E1EB; **Location:** country: Serbia; locality: Bajmok, Čenej, Đurđin, Mišićevo, Srbobran; **Event:** samplingProtocol: Sweep net, Oilseed pods; eventDate: 27.04.2018, 10.05.2018, 25.05.2018, 12.06.2019, 13.06.2019; habitat: oilseed rape, semi-natural habitat

##### Parasite of

*Ceutorhynchusassimilis*

##### Notes

oilseed rape pest host: yes

#### Mesopolobus sp. 1

1BA8643B-D63F-5E4D-9398-77405E666AA1

##### Materials

- **Type status:** Other material. **Occurrence:** individualCount: 10 males; behavior: primary parasitoids, larval; occurrenceID: 33B2176A-BC4D-5619-822B-22D79B58F442; **Location:** country: Serbia; locality: Čenej; **Event:** samplingProtocol: Pan traps; eventDate: 04-07.05.2018, 07-10.05.2018; habitat: oilseed rape

##### Parasite of

*Ceutorhynchus* spp.

##### Notes

oilseed rape pest host: unknown, possible

#### Mesopolobus sp. 2

B3D5D5D7-2632-5A67-AA56-853908B008E9

##### Materials

- **Type status:** Other material. **Occurrence:** individualCount: 3 males; behavior: primary parasitoids, larval; occurrenceID: 3D0EEB7D-A975-53E6-B51B-B046F124045D; **Location:** country: Serbia; locality: Čenej, Srbobran; **Event:** samplingProtocol: Sweep net, Pan traps; eventDate: 24-27.04.2018, 10.05.2018; habitat: oilseed rape

##### Parasite of

*Ceutorhynchus* spp.

##### Notes

oilseed rape pest host: unknown, possible

#### Mesopolobus sp. 3

29E57BE9-A0A4-5E48-88E0-CB9069BF2CF2

##### Materials

- **Type status:** Other material. **Occurrence:** individualCount: 2 males, 1 female; behavior: primary parasitoids, larval; occurrenceID: E5605028-C316-573A-90BC-AA00987B6E8D; **Location:** country: Serbia; locality: Čenej, Srbobran; **Event:** samplingProtocol: Sweep net, Pan traps; eventDate: 04-07.05.2018, 10.05.2018; habitat: oilseed rape, semi-natural habitat

##### Parasite of

*Ceutorhynchus* spp.

##### Notes

oilseed rape pest host: unknown, possible

#### Pteromalus sp. 1

360D10C5-777C-5089-B40B-8585DB89038A

##### Materials

- **Type status:** Other material. **Occurrence:** individualCount: 2 males, 3 females; behavior: primary parasitoids, pupal; occurrenceID: 963C73AA-A9C4-5BA1-9EA7-1B189335F65C; **Location:** country: Serbia; locality: Čenej, Srbobran; **Event:** samplingProtocol: Sweep net; eventDate: 27.04.2018, 04.05.2018, 25.05.2018; habitat: oilseed rape

##### Parasite of

Lepidoptera, Tenthredinidae

##### Notes

oilseed rape pest host: unknown

#### Pteromalus sp. 2

92438A40-4F66-5470-958C-0C3352496FAF

##### Materials

- **Type status:** Other material. **Occurrence:** individualCount: 1 male; behavior: primary parasitoids, pupal; occurrenceID: 9F2959EB-40AA-5EBB-8492-CEC29BDA2C93; **Location:** country: Serbia; locality: Mišićevo; **Event:** samplingProtocol: Sweep net; eventDate: 04-25-19; habitat: semi-natural habitat

##### Parasite of

Lepidoptera, Tenthredinidae

##### Notes

oilseed rape pest host: unknown

#### Pteromalus sp. 3

C77860B5-AB7F-5D4B-B70C-62C7E093A125

##### Materials

- **Type status:** Other material. **Occurrence:** individualCount: 2 males; behavior: primary parasitoids, pupal; occurrenceID: E8377C37-16CC-5F4F-ADF7-46E4C90C4146; **Location:** country: Serbia; locality: Mišićevo; **Event:** samplingProtocol: Sweep net; eventDate: 25.04.2019, 10.05.2019; habitat: semi-natural habitat

##### Parasite of

Lepidoptera, Tenthredinidae

##### Notes

oilseed rape pest host: unknown

#### Trichomalus lucidus

(Walker, 1835)

D852C286-4E23-5791-B6F4-8ED1BF8E0474

##### Materials

- **Type status:** Other material. **Occurrence:** individualCount: 244 males, 150 females; behavior: primary parasitoids, larval; occurrenceID: 9E42182F-CD4C-58E3-A006-500646839E1B; **Location:** country: Serbia; locality: Bajmok, Čenej, Đurđin, Pačir, Srbobran; **Event:** samplingProtocol: Sweep net, Pan traps, Aphid colony; eventDate: 27.04.2018, 04-07.05.2018, 07-10.05.2018, 25.05.2018, 22-24.04.2019, 08.05.2019, 13.06.2019; habitat: oilseed rape, semi-natural habitat

##### Parasite of

*Ceutorhynchus* spp., *Psylliodeschrysocephala*

##### Notes

oilseed rape pest host: yes

#### Trichomalus sp. 1

67C66601-6C76-570B-820F-822CFB257AA2

##### Materials

- **Type status:** Other material. **Occurrence:** individualCount: 3 males, 1 female; behavior: primary parasitoids, larval; occurrenceID: C0DFE327-FD39-5199-A973-1F370CF44F89; **Location:** country: Serbia; locality: Čenej, Srbobran; **Event:** samplingProtocol: Pan traps; eventDate: 04-07.05.2018, 07-10.05.2018; habitat: oilseed rape, semi-natural habitat

##### Parasite of

*Ceutorhynchus* spp.

##### Notes

oilseed rape pest host: unknown, possible

#### Trichomalus sp. 2

C660C84D-F7B1-5E43-8E18-D754311B5D37

##### Materials

- **Type status:** Other material. **Occurrence:** individualCount: 2 males, 4 females; behavior: primary parasitoids, larval; occurrenceID: 5B64E486-9913-590C-9EA2-3C7FEAF2EAD7; **Location:** country: Serbia; locality: Čenej, Srbobran; **Event:** samplingProtocol: Pan traps; eventDate: 04-07.05.2018, 07-10.05.2018; habitat: oilseed rape

##### Parasite of

*Ceutorhynchus* spp.

##### Notes

oilseed rape pest host: unknown, possible

#### Trichomalus sp. 3

BA4E6B00-D0CC-5515-A9E0-FC6C5C8EE19E

##### Materials

- **Type status:** Other material. **Occurrence:** individualCount: 1 male; behavior: primary parasitoids, larval; occurrenceID: 08BC1E84-4526-5A64-A66F-11A613C156A9; **Location:** country: Serbia; locality: Čenej; **Event:** samplingProtocol: Sweep net; eventDate: 05-10-18; habitat: oilseed rape

##### Parasite of

*Ceutorhynchus* spp.

##### Notes

oilseed rape pest host: unknown, possible

#### Trichomalus sp. 4

E4A430BD-999A-5825-A056-6D276181CF62

##### Materials

- **Type status:** Other material. **Occurrence:** individualCount: 1 male; behavior: primary parasitoids, larval; occurrenceID: 635BACFB-24DC-51D4-B87A-E94BBACEAD18; **Location:** country: Serbia; locality: Srbobran; **Event:** samplingProtocol: Pan traps; eventDate: 04-07.05.2018; habitat: semi-natural habitat

##### Parasite of

*Ceutorhynchus* spp.

##### Notes

oilseed rape pest host: unknown, possible

#### Scelionidae

FA2AE6A2-6E96-5C5E-890C-E97948468FE6

#### Scelionidae sp. 1

417A58D8-59C5-5E45-8BBA-824BDA5465CA

##### Materials

- **Type status:** Other material. **Occurrence:** individualCount: 1 female; behavior: primary parasitoids; occurrenceID: 155A11AF-C04E-5ACF-B427-5945A80564DA; **Location:** country: Serbia; locality: Srbobran; **Event:** samplingProtocol: Sweep net; eventDate: 05-07-18; habitat: oilseed rape

##### Parasite of

Insects, arachnids

##### Notes

oilseed rape pest host: unknown

#### Eumicrosoma sp. 1

D732E8F6-B707-5DFB-BCFE-6EDC0343A62D

##### Materials

- **Type status:** Other material. **Occurrence:** individualCount: 1 male; behavior: primary parasitoids, egg; occurrenceID: 80F2947C-A65A-5166-98C3-25C427411C13; **Location:** country: Serbia; locality: Srbobran; **Event:** samplingProtocol: Sweep net; eventDate: 05-07-18; habitat: oilseed rape

##### Parasite of

Heteroptera, Pentatomidae, Lygaeidae

##### Notes

oilseed rape pest host: unknown

#### Gryon sp. 1

4BC71F2E-ED0E-5050-9569-495A211981CE

##### Materials

- **Type status:** Other material. **Occurrence:** individualCount: 1 male, 2 females; behavior: primary parasitoids, egg; occurrenceID: 8D48E981-9573-5984-A23B-2C7D628D0D7B; **Location:** country: Serbia; locality: Srbobran; **Event:** samplingProtocol: Sweep net, Pan traps; eventDate: 24-27.04.2018, 07.05.2018, 10.05.2018; habitat: oilseed rape

##### Parasite of

Hemiptera, Coreidae

##### Notes

oilseed rape pest host: unknown

#### Trimorus sp. 1

621406B7-2E5A-5871-B151-4090CFC86FBF

##### Materials

- **Type status:** Other material. **Occurrence:** individualCount: 1 male, 1 female; behavior: primary parasitoids, egg; occurrenceID: 02F2A15C-4302-5C1A-908C-8F0612805AEE; **Location:** country: Serbia; locality: Bajmok, Srbobran; **Event:** samplingProtocol: Sweep net; eventDate: 07.05.2018, 24.04.2019; habitat: oilseed rape

##### Parasite of

Carabidae

##### Notes

oilseed rape pest host: unknown

#### Trissolcus basalis

(Wollaston, 1858)

BBE95507-033B-5A8F-A5A7-926084F26853

##### Materials

- **Type status:** Other material. **Occurrence:** individualCount: 14 males, 5 females; behavior: primary parasitoids, egg; occurrenceID: F640CCB8-EB59-5547-8493-53E50E3F9240; **Location:** country: Serbia; locality: Bajmok, Čenej, Srbobran; **Event:** samplingProtocol: Sweep net, Pan traps; eventDate: 24-27.04.2018, 04-07.05.2018, 07-10.05.2018, 25.05.2018, 13.06.2019; habitat: oilseed rape, semi-natural habitat

##### Parasite of

Heteroptera, *Nezaraviridula*

##### Notes

oilseed rape pest host: unknown

#### Spalangiidae

96903F77-5BC2-59FD-BC92-4E5BAC83E1E8

#### Spalangia nigra

Latreille, 1805

4AF77AF1-927A-54F0-BEF4-F85255011B74

##### Materials

- **Type status:** Other material. **Occurrence:** individualCount: 3 males; behavior: primary parasitoids, pupal; occurrenceID: E0BC2BE7-A9F1-5930-B13D-8B4980F0D92D; **Location:** country: Serbia; locality: Čenej, Pačir; **Event:** samplingProtocol: Pan traps; eventDate: 07-10.05.2018, 22-24.04.2019; habitat: oilseed rape, semi-natural habitat

##### Parasite of

Diptera puparia

##### Notes

oilseed rape pest host: unknown

#### Systasidae

590B8276-ABED-5358-A3CE-0EC20415F12E

#### Asaphes vulgaris

Walker, 1834

9E481608-47E1-59AD-AF17-AAB198031E43

##### Materials

- **Type status:** Other material. **Occurrence:** individualCount: 2 males; behavior: secondary parasitoids, larval; occurrenceID: 0354FF52-0A62-5246-8340-A904585A61BD; **Location:** country: Serbia; locality: Čenej, Srbobran; **Event:** samplingProtocol: Sweep net; eventDate: 10.05.2018, 25.05.2018; habitat: oilseed rape

##### Parasite of

aphid parasitoids

##### Notes

oilseed rape pest host: yes

#### Torymidae

8C08303E-0B4F-584A-9A9E-D4D02820D14F

#### Podagrion pachymerum

(Walker, 1833)

62351D74-505B-57ED-AB6D-9F45D2408C74

##### Materials

- **Type status:** Other material. **Occurrence:** individualCount: 1 male; behavior: primary parasitoids, egg; occurrenceID: D3EA9F47-C288-59B0-8F97-02BF3560AC92; **Location:** country: Serbia; locality: Mišićevo; **Event:** samplingProtocol: Sweep net; eventDate: 04-25-19; habitat: semi-natural habitat

##### Parasite of

Mantodea, Mantidae

##### Notes

oilseed rape pest host: unknown

#### Pseudotorymus napi

(Amerling & Kirchner, 1860)

F1AD607D-8833-58B4-92DB-3494E32CF769

##### Materials

- **Type status:** Other material. **Occurrence:** individualCount: 144 males, 74 females; behavior: primary parasitoids, larval; occurrenceID: A46E8930-EDC8-58B9-9BBA-D60067B74506; **Location:** country: Serbia; locality: Čenej, Mišićevo, Srbobran; **Event:** samplingProtocol: Sweep net, Pan traps, Aphid colony, Oilseed pods; eventDate: 24-27.04.2018, 04-07.05.2018, 07-10.05.2018, 25.05.2018, 25.05.2019; habitat: oilseed rape, semi-natural habitat

##### Parasite of

*Dasineurabrassicae*

##### Notes

oilseed rape pest host: yes

#### Torymus sp. 1

450E7843-C598-5B21-96EC-06C963CC7E62

##### Materials

- **Type status:** Other material. **Occurrence:** individualCount: 1 male; behavior: primary parasitoids, larval; occurrenceID: 59EE6B5E-7094-559B-BD13-2305E7E082C7; **Location:** country: Serbia; locality: Pačir; **Event:** samplingProtocol: Sweep net; eventDate: 04-25-19; habitat: oilseed rape

##### Parasite of

Ectoparasitoids of gall-forming insects (Cecidomyiidae, Cynipidae)

##### Notes

oilseed rape pest host: unknown

#### Trichogrammatidae

D1CF8304-F365-530A-8782-70A1C59EC692

#### Trichogrammatidae sp. 1

6B4C452E-7FE2-55CB-9938-525196BE8E04

##### Materials

- **Type status:** Other material. **Occurrence:** individualCount: 1 male; behavior: primary parasitoids, egg; occurrenceID: 844E2307-1DB9-578E-9CE2-2A8A79EE2C32; **Location:** country: Serbia; locality: Bajmok; **Event:** samplingProtocol: Sweep net; eventDate: 04-24-19; habitat: oilseed rape

##### Parasite of

Lepidoptera, Coleoptera, Neuroptera, Diptera, Hymenoptera

##### Notes

oilseed rape pest host: unknown

#### Trichogramma evanescens

Westwood, 1833

4B78B8F0-490E-573B-9C65-6797D4F50EED

##### Materials

- **Type status:** Other material. **Occurrence:** individualCount: 1 male; behavior: primary parasitoids, egg; occurrenceID: 1E041ED8-6884-5503-B24E-7115C06B7382; **Location:** country: Serbia; locality: Srbobran; **Event:** samplingProtocol: Pan traps; eventDate: 24-27.04.2018; habitat: semi-natural habitat

##### Parasite of

Lepidoptera, Chrysomelidae

##### Notes

oilseed rape pest host: unknown

## Analysis

During this two-year study, we found a total of 3135 specimens of primary or secondary parasitoids, of which 2855 were found in oilseed rape fields and 280 in semi-natural habitats. In the 2018 season, we found 2926 individuals, while in 2019, we found only 209 individuals. The temporal dynamics of parasitoid abundance were also different in the two years. The highest abundance was in May in 2018 (2694) and in April in 2019 (107). Most individuals were found when sampling with sweep nets (1715), followed by rearing from aphid colonies (1001), pan traps (404) and collecting oilseed pods (15).

**Figure 2.**
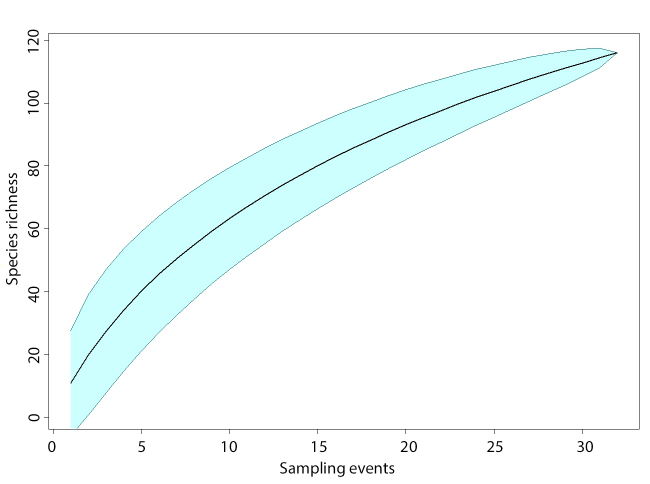
Species accumulation curve for oilseed rape fields. Light blue area represents 95% confidence intervals.

We found 153 different taxa, of which 119 were found in oilseed rape fields and 87 in semi-natural habitats. Not all specimens could be identified to species level, but only to genus or family level. We found a total of 92 different genera, 73 in oilseed rape fields and 59 in semi-natural habitats and 22 families, all of which occurred in oilseed rape and 19 in semi-natural habitats. The species accumulation curve shows that the number of detected species in oilseed rape fields has not reached a plateau (Fig. 2), while extrapolated species richness differed somewhat between different estimators (Chao = 173.2 ± 21.5, Jack1 = 165.4 ± 12.3, Jack2 = 193.2, Bootstrap = 138.1 ± 6.8). The number of singletons and doubletons in oilseed rape fields was 45 and 19, respectively.

The four most abundant families were Braconidae (1880), Pteromalidae (500), Torymidae (220) and Eulophidae (123) with more than 85% of all specimens captured and the four most numerous genera were *Diaeretiella* Starý, 1960 (1243), *Trichomalus* Thomson, 1878 (406), *Aphidius* Nees, 1818 (389) and *Pseudotorymus* Masi, 1921 (218) with more than 70%. The number of different taxa found in each family, sorted by number of taxa is shown in Table 3.

Of all the taxa we found, 119 are described in literature as primary parasitoids and five as hyperparasitoids, with another 29 that can be either primary or secondary parasitoids. Based on the literature examined, in both habitats, we found 31 genera (33 species) described as parasitoids of oilseed rape pests and 54 genera (97 species) parasitising non-pest species. We also identified 10 genera (23 species) as possible parasitoids of oilseed rape pests. These taxa could only be identified to the genus level and belong to genera that have at least one other species of oilseed rape pest parasitoids. In oilseed rape fields, we found 28 genera (29 species) of oilseed rape pest parasitoids, 40 genera (73 species) parasitising non-pest species and eight genera (17 species) of possible oilseed rape pest parasitoids.

Using two criteria for consistency of occurrence, we selected 25 taxa (22 genera) that regularly occur in our oilseed rape fields, of which 13 taxa (11 genera) were not reported as parasitoids of oilseed rape pests, although four taxa are identified as possible parasitoids (Table 4).

Detailed information on locations, collection dates, habitat, sampling methods, host, parasitism type, oilseed rape pest host status and number of specimens for each taxon can be found in Suppl. material 2 and Suppl. material 3 and additional data on host stage and references in Suppl. material 4.

## Discussion

Oilseed rape fields are known to harbour a variety of parasitoids, which are natural enemies of oilseed rape pests (Alford 2003, Williams 2010). However, we have found that the diversity of parasitoids found in our oilseed rape fields goes beyond those associated with crop pests. In fact, only slightly less than 25% of the parasitoid taxa identified in oilseed rape fields are linked to crop pest species. This suggests that oilseed rape fields serve multiple functions for parasitoids, including providing host species that are not pests and floral resources that attract parasitoids. Although the aim of this study was not to conduct a detailed analysis of differences between oilseed rape fields and nearby semi-natural habitats, we observed a noticeably higher abundance and diversity of parasitoids in oilseed rape fields compared to nearby semi-natural habitats. The presence of a diverse parasitoid community in fields, including those not typically associated with oilseed rape pests, may contribute to the natural pest control of other crops and promote biodiversity in the surrounding environment. However, it is worth noting that some parasitoid species might also provide disservice to biological control by parasitising on other beneficial insects, like *Diplazonlaetatorius* that can parasitise hoverfly pupae (Townes 1971), *Asaphesvulgaris* which is hyperparasitoid of several of the aphid parasitoids (Žikić et al. 2012) and *Podagrion* species that specialise on praying mantis eggs (Thompson 1958).

There are several taxa that were collected consistently and in greater numbers in our oilseed rape fields, but are not known to parasitise oilseed rape pest species. *Triaspisthoracica* was the most numerous of them and parasitises species of the genus *Bruchus* (Chrysomelidae), which are important pests in various types of bean crops, such as peas, lentils and vetches (Yu et al. 2011). In 2018, it was found in four fields, but only in one field in large numbers (65 out of a total of 70 individuals). Although this cannot be verified, it is possible that this particular field had a high understorey of some Fabaceae species that provided resources for the *Bruchus* population and consequently for *T.thoracica*. Similarly, *Trissolcusbasalis* is an egg parasitoid of invasive pentatomid species, such as *Nezaraviridula* and *Halyomorphahalys* (Stål, 1855), which are pests in many legumes, fruits and vegetables (Powell and Shepard 1982, Schaefer and Panizzi 2000, Zapponi et al. 2021). The sporadic presence of other plant species in the oilseed rape fields, such as wild grasses, may also possibly explain the presence of *Collyriacoxator*, a specialised parasitoid of the European wheat stem sawfly (*Cephuspygmaeus* (Linnaeus, 1767)), which is a pest of cereal crops, but also feeds on various grass species. Likewise, *Telenomus* species parasitise various Heteroptera, but also Lepidoptera, which can be readily found as non-pests in oilseed rape fields. On the other hand, *Chrysolampusthenae* is known to parasitise *Lamiogethespedicularis* (Gyllenhal, 1808) which are found on Lamiaceae plants (Noyes 2019). It is possible that this parasitoid also attacks other species from the subfamily Meligethinae, such as *B.aeneus* or that host plants were present in the undergrowth. Given the significant presence of these parasitoids not associated with pest species, it would be valuable for future studies to note the previous season's crop as this may help explain the presence of volunteer plants and associated pests and parasitoids.

There are also some parasitoid species that are widely distributed in oilseed rape throughout Europe, but are absent or present only in small numbers in our study. According to Nilsson (2003), the most important pollen beetle parasitoid species are *Phradisinterstitialis* (Thomson, 1889), *P.morionellus* (Holmgren, 1860) and *Tersilochusheterocerus*, with *Diospiluscapito*, *Blacusnigricornis* and *Brachyserphusparvulus* (Nees, 1834) also being quite common. Only *T.heterocerus* was found in our study in noteworthy numbers to be considered as an important biological control agent. Two other species were found in very low numbers, *D.capito* only in semi-natural habitats and *B.nigricornis* in both habitats, while other species were not found. Interestingly, the most abundant pollen beetle parasitoid in our study was *Eubazussigalphoides*, previously reported only from France and Poland (Nilsson 2003, Ulber et al. 2010). Likewise, for the cabbage seed weevil (*C.obstrictus*), only one species (*Mesopolobusmorys*) of the three most common larval ectoparasitoids (*Stenomalinagracilis* (Walker, 1834), *Trichomalusperfectus* (Walker, 1835)) was detected in our study. We also found the two most common parasitoids of the brassica pod midge in Europe, *Platygastersubuliformis* and *Omphaleclypealis*, but both in low abundance, while the dominant species in our study were *Pseudotorymusnapi* and *Aphanogmusabdominalis*. The second most abundant parasitoid in our study was the pteromalid *Trichomaluslucidus*, which parasitises the cabbage stem flea beetle (*P.chrysocephala*) and the cabbage stem weevil (*C.pallidactylus*). This species is one of eight recorded parasitoids of the cabbage stem flea beetle and of several parasitoids of the cabbage stem weevil (Ulber 2003). Other parasitoid species of these two pests were either not observed or observed in very low numbers. Todorov et al. (2022) recently presented a checklist of the pteromalid fauna in Bulgarian oilseed rape fields and recorded 26 taxa, but, interestingly, did not find any *Trichomalus* species. Parasitoids of the cabbage flea beetle (*P.nemorum*) are represented only by *Townesilitusbicolor*, while other known species are absent or could not be determined at the species level, but only at the genus level. The most numerous group of parasitoids we found were aphid parasitoids, although their dominance was partly influenced by sampling methods, as about half of the specimens were from aphid rearing. *Diaeretiellarapae* and *Praonvolucre* are known parasitoids of both cabbage aphid (*B.brassicae*) and peach-potato aphid (*M.persicae*), while *Aphiduservi* and *A.matricariae* parasitise *M.persicae* (Tomanović et al. 2021).

It is unclear why some of the parasitoid species, which are widespread and numerous in various previously studied parts of Europe, do not occur at all or in greater numbers in Serbia. Nilsson (2003), while noting that there are no records from most of the Balkans, states that the parasitoid wasp fauna of this region should largely resemble that of Central Europe and that all Central European species should probably be present here. This is a reasonable expectation since, for example, the three most important pollen beetle parasitoids (*P.morionellus*, *P.interstitialis* and *T.heterocerus*) occur in neighbouring Hungary (all three species) and Bulgaria (*T.heterocerus*) (van Achterberg and Zwakhals 2023). Furthermore, since the Balkans is a hotspot of biodiversity in Europe, the parasitoid wasp fauna of Serbia can be expected to be richer than that of Central Europe (Griffiths et al. 2004). Petrović (2022) compared the parasitoid wasp fauna of the subfamily Aphidiinae from different European countries and found that only the Czech fauna had greater diversity (about 135 species) than the Serbian fauna (121 species), while other countries, such as Germany (109), the United Kingdom (96) and Norway (26), had fewer species, although the Czech fauna may simply be much better studied. To date, only Todorov et al. (2022) have published a partial list of oilseed parasitoids for one Balkan country, Bulgaria and only for the family Pteromalidae. However, only two species (*Mesopolobusincultus* and *M.morys*) are recorded in both Bulgaria and Serbia, with the possible existence of several other taxa of the genera *Mesopolobus* and *Pteromalus* that are difficult to identify to species level.

We obtained the most specimens by two sampling methods, sweep-net sampling (about 1700) and rearing from aphid colonies (about 1000). However, it should be noted that sampling with sweep nets and pan traps yielded more diverse samples (106 and 88 taxa, respectively), although there was no way to directly associate the collected specimens with host pests or plants. On the other hand, rearing from oilseed pods or from aphid colonies provided more targeted samples of either seed pod pests (such as *D.brassicae* and *C.obstrictus*) or aphid species (*B.brassicae* and *M.persicae*). Most of the detected parasitoid families, with meaningfully large number of individuals, tended to be collected with sweep-net sampling, with the exception of Platygastridae and Mymaridae, which tended to occur more in pan traps. Therefore, the choice of sampling method can considerably limit the range of species detected and should be adapted to the research questions. Based on the examination of the species accumulation curve for samples from oilseed rape fields, it is clear that observed species richness obtained through our combined sampling methodology did not reach a plateau and that actual species richness is even higher. This is further corroborated by different species richness estimators which show a range of values, from 138 species as lowest estimate to 193 species as highest. We also found a seemingly large proportion of rare species (singletons and doubletons) in the oilseed rape fields in our study. About 38% were found only with one specimen and about 19% with two individuals. However, Scharff et al. (2003) give an overview of undersampling bias in terrestrial arthropod surveys and argue that, even in the “state of the art” arthropod surveys, about 50–70% of species are known from just one or two individuals.

### Conclusions

Our study of the parasitoid community associated with oilseed rape fields revealed great diversity of parasitoid species. Interestingly, the majority of these parasitoids were not known to parasitise oilseed rape pest species, suggesting the existence of alternative hosts or other ecological interactions within oilseed rape fields. Additional factors should be carefully measured, such as the previous season's crop in each field, the presence of volunteer plants and weed species and associated herbivores, to gain a better insight into the various interactions that appear to exist in oilseed rape fields. We also observed notable differences in the occurrence patterns of different parasitoid species in Serbia and other parts of Europe, indicating the need for further studies of the ecological dynamics and host associations of parasitoid species in oilseed rape fields. Understanding the factors affecting the presence, abundance and distribution of parasitoids will contribute to more effective regional and local pest management strategies.

## Supplementary Material

41AEB522-831F-5759-9C58-47FB3CAFC0ED 10.3897/BDJ.11.e110118.suppl1 Supplementary material 1 Supplements Table 1. List of identification keys used. Data type List of references File: oo_925066.docx https://binary.pensoft.net/file/925066 Milan Plećaš, Vladimir Žikić, Korana Kocić, Jelisaveta Čkrkić, Anđeljko Petrović, Željko Tomanović

D01E7ABE-231B-53BE-904F-A7FD5BFD1B23 10.3897/BDJ.11.e110118.suppl2 Supplementary material 2 Supplement Table 2a. Checklist of parasitoids found in oilseed rape fields in Serbia Data type Checklist with additional data Brief description Detailed information on locations, collection dates, habitat, sampling methods, parasitism type, oilseed rape pest host status and number of specimens for each taxon. File: oo_925070.docx https://binary.pensoft.net/file/925070 Milan Plećaš, Vladimir Žikić, Korana Kocić, Jelisaveta Čkrkić, Anđeljko Petrović, Željko Tomanović

92F16471-9346-59B0-9532-C22168B07EB0 10.3897/BDJ.11.e110118.suppl3 Supplementary material 3 Supplement Table 2b. Checklist of parasitoids found in oilseed rape fields in Serbia in csv format Data type Checklist with additional data Brief description Detailed information on locations, collection dates, habitat, sampling methods, parasitism type, oilseed rape pest host status and number of specimens for each taxon provided in cvs format. File: oo_944793.csv https://binary.pensoft.net/file/944793 Milan Plećaš, Vladimir Žikić, Korana Kocić, Jelisaveta Čkrkić, Anđeljko Petrović, Željko Tomanović

19ABB76D-8023-5142-8B5E-605457B72338 10.3897/BDJ.11.e110118.suppl4 Supplementary material 4 Supplement Table 3. Checklist of parasitoids found in oilseed rape fields in Serbia with additional host information. Data type Checklist with additional data Brief description Detailed information on OSR pest host status, parasitism type, host species and host stage and references. File: oo_925071.docx https://binary.pensoft.net/file/925071 Milan Plećaš, Vladimir Žikić, Korana Kocić, Jelisaveta Čkrkić, Anđeljko Petrović, Željko Tomanović

## Figures and Tables

**Table 1.** List of major and minor oilseed rape pests (Alford 2003, Williams 2010)

	Latin name	Common name	Family
Major oilseed rape pests			
1	*Brassicogethes* Audisio & Cline, 2009	pollen beetles	Nitidulidae
2	*Psylliodeschrysocephala* Linnaeus, 1758	cabbage stem flea beetle	Chrysomelidae
3	*Ceutorhynchusobstrictus* (Marsham, 1802)	cabbage seed weevil	Curculionidae
4	*Ceutorhynchuspallidactylus* (Marsham, 1802)	cabbage stem weevil	Curculionidae
5	*Ceutorhynchusnapi* Gyllenhal, 1837	rape stem weevil	Curculionidae
6	*Dasineurabrassicae* (Winnertz, 1853)	cabbage pod midge	Cecidomyiidae
Minor oilseed rape pests			
1	*Ceutorhynchuspicitarsis* Gyllenhal, 1837	rape winter stem weevil	Curculionidae
2	*Phyllotretanemorum* (Linnaeus, 1758)	cabbage flea beetle	Chrysomelidae
3	*Athaliarosae* (Linnaeus, 1758)	turnip sawfly	Tenthredinidae
4	*Brevicorynebrassicae* (Linnaeus, 1758)	cabbage aphid	Aphididae
5	*Myzuspersicae* (Sulzer, 1776)	peach-potato aphid	Aphididae

**Table 2.** Coordinates of the sampled oilseed rape fields.

	**South Bačka region**					**North Bačka region**			
	**Čenej**			**Srbobran**		**Bajmok**	**Đurđin**	**Mišićevo**	**Pačir**
**Latitude**	45.3241	45.3468	45.3296	45.5216	45.5501	45.9516	45.9631	45.9892	45.8936
**Longitude**	19.8481	19.8615	19.8313	19.7571	19.7371	19.4771	19.5129	19.5126	19.4088

**Table 3.** The number of parasitoid taxa found in each family in two habitats.

**Total**		**Oilseed rape fields**		**Semi-natural habitats**	
**Family**	**No. of taxa**	**Family**	**No. of taxa**	**Family**	**No. of taxa**
Braconidae	29	Braconidae	22	Braconidae	16
Pteromalidae	25	Pteromalidae	20	Eulophidae	11
Ichneumonidae	18	Eulophidae	14	Pteromalidae	11
Eulophidae	17	Ichneumonidae	12	Ichneumonidae	10
Mymaridae	11	Mymaridae	10	Platygastridae	8
Platygastridae	10	Platygastridae	8	Encyrtidae	6
Encyrtidae	8	Scelionidae	5	Mymaridae	6
Ceraphronidae	5	Encyrtidae	4	Ceraphronidae	4
Scelionidae	5	Figitidae	4	Ceidae	2
Figitidae	4	Ceraphronidae	3	Diapriidae	2
Perilampidae	3	Perilampidae	3	Figitidae	2
Torymidae	3	Bethylidae	2	Torymidae	2
Bethylidae	2	Megaspilidae	2	Chalcididae	1
Ceidae	2	Torymidae	2	Eurytomidae	1
Diapriidae	2	Ceidae	1	Megaspilidae	1
Megaspilidae	2	Chalcididae	1	Perilampidae	1
Trichogrammatidae	2	Diapriidae	1	Scelionidae	1
Chalcididae	1	Eurytomidae	1	Spalangiidae	1
Eurytomidae	1	Pirenidae	1	Trichogrammatidae	1
Pirenidae	1	Spalangiidae	1		
Spalangiidae	1	Systasidae	1		
Systasidae	1	Trichogrammatidae	1		

**Table 4.** List of taxa consistently found in oilseed rape fields.

**Taxon**	**Oilseed rape pest host**	**No. of specimens**	**No. of fields**
*Diaeretiellarapae* (McIntosh, 1855)	*Myzuspersicae*, *Brevicorynebrassicae*	1197	9
*Trichomaluslucidus* (Walker, 1835)	*Psylliodeschrysocephala*, *Ceutorhynchuspallidactylus*	392	6
*Aphidiuservi* Haliday, 1834	*Myzuspersicae*, *Brevicorynebrassicae*	270	6
*Pseudotorymusnapi* (Amerling & Kirchner, 1860)	*Dasineurabrassicae*	217	5
*Aphidiusmatricariae* Haliday, 1834	*Myzuspersicae*, *Brevicorynebrassicae*	112	4
*Triaspisthoracica* (Curtis, 1860)	unknown	70	4
*Eubazussigalphoides* (Marshall, 1889)	*Brassicogethesaeneus* (Fabricius, 1775)	52	5
*Mesopolobusmorys* (Walker, 1848)	*Ceutorhynchusobstrictus*	27	6
*Praonvolucre* (Haliday, 1833)	*Myzuspersicae*, *Brevicorynebrassicae*	27	5
*Anaphes* sp. 1	unknown/possible	24	7
*Aphanogmusabdominalis* (Thomson, 1858)	*Dasineurabrassicae*	23	3
*Townesilitusbicolor* (Wesmael, 1835)	*Phyllotretanemorum*	20	3
*Eulophus* sp. 1	unknown/possible	18	6
*Collyriacoxator* (Villers, 1789)	unknown	18	3
*Trissolcusbasalis* (Wollaston, 1858)	*Nezaraviridula* (Linnaeus, 1758)	17	4
*Tersilochusheterocerus* (Thomson, 1889)	*Brassicogethesaeneus*	17	4
*Aprostocetus* sp. 1	unknown/possible	16	7
*Telenomus* sp. 3	unknown	15	7
*Chrysolampusthenae* (Walker, 1848)	unknown	15	3
*Telenomus* sp. 4	unknown	14	5
*Telenomus* sp. 1	unknown	13	5
*Lagynodespallidus* (Boheman, 1832)	unknown	12	4
*Tetrastichus* sp. 1	unknown/possible	12	3
*Spalangiopelta* sp. 1	unknown	11	5
*Apanteles* sp. 1	unknown	10	3
