# Supplementary material for: Diversity of parasitoid wasps (Insecta, Hymenoptera) in oilseed rape fields in Serbia
Source: Biodivers Data J. 2023 Dec 5;11:e110118. doi: 10.3897/BDJ.11.e110118 (PMC10716848; doi:10.3897/BDJ.11.e110118)
Supplement: Supplementary material 1 — Supplements Table 1. List of identification keys used. [file bdj-11-e110118-s001.docx]

Supplements

Table 1. List of identification keys used.

| Alford DV (2003) Biocontrol of oilseed rape pests. Blackwell Science Ltd. 355 pp. <https://doi.org/10.1002/9780470750988> |
| --- |
| Askew RR, Bouček Z (1968) Index of Palaearctic Eulophidae (excl. Tetrastichinae). In: Index of entomophagous insects 1. Delucchi V, Remaudière G (eds). 223 pp. Paris |
| Bouček Z, Rasplus JY (1991) Illustrated Key to West-Palearctic Genera of Pteromalidae (Hymenoptera: Chalcidoidea). INRA: Paris. |
| Broad G, (2011) Identification key to the subfamilies of Ichneumonidae (Hymenoptera). 52 pp. London |
| Burks R, Mitroiu M-D, Fusu L, Heraty JM, Janšta P, Heydon S, Papilloud ND-S, Peters RS, Tselikh EV, Woolley JB, van Noort S, Baur H, Cruaud A, Darling C, Haas M, Hanson P, Krogmann L, Rasplus J-Y (2022) From hell’s heart I stab at thee! A determined approach towards a monophyletic Pteromalidae and reclassification of Chalcidoidea (Hymenoptera). Journal of Hymenoptera Research **94**, 13-88. <https://doi.org/10.3897/jhr.94.94263> |
| Buhl PN (1999) A synopsis of the Platygastridae of Fennoscandia and Denmark. Entomofauna **20**, 17–52. |
| Dessart P (1964) Contribution à l’étude des Hyménoptères Proctotrupoidea (IV). Trois Ceraphronidae parasites de la Cecidomyie du colza: *Dasyneura brassicae* (Winnertz), en France. Bulletin et Annales de la Société Royale d’Entomologie de Belgique **100**, 109–30. |
| Gauld I, Bolton B (1988) The Hymenoptera. British Museum of Natural History and Oxford University Press: London. |
| Goulet H, Huber JT (1993) Hymenoptera of the world: an identification guide to families. Agriculture Canada, Research Branch, Publication 1894/E, 668 pp. |
| Graham MWR de V (1959) Keys to the British genera and species of Elachertinae, Eulophinae, Entedontinae and Euderinae (Hym., Chalcidoidea). Trans. soc. Br. ent., **13**, 169-204. |
| Graham MWR de V (1969) The Pteromalidae of north-western Europe (Hymenoptera: Chalcidoidea). Bulletin of the British Museum (Natural History) Entomology Series, Supplement **16**, 908 pp. |
| Graham MWR de V (1987) A reclassification of the European Tetrastichinae (Hymeoptera: Eulophidae), with a revision of certain genera. Bulletin of the British Museum (Natural History) Entomology Series **55**, 1–392. |
| Graham MWR de V (1987) A reclassification of the European Tetrastichinae (Hymenoptera: Eulophidae), with a revision of certain genera. Bulletin of the British Museum (Natural History), Entomology **55**(1), 1-392. |
| Graham MWR de V, Gijswijt MJ (1998) Revision of the European species of *Torymus* Dalman (Hymenoptera: Torymidae). Zoologische Verhandelingen, **317**(1), 1-202. |
| Horstmann K (1971) Revision der europäischen Tersilochinen (Hymenoptera, Ichneumonidae) Teil I. Veröffentlichungen der Zoologischen Staatsammlung München **15**, 45–138. |
| Huber JT, Thuroczy C (2018) Review of *Anaphes* Haliday (Hymenoptera: Mymaridae) with key to species in Europe and a world catalogue. Zootaxa, **4376**(1), 1-104. |
| Janšta P, Cruaud A, Delvare G, Genson G, Heraty J, Křížková B, Rasplus JY (2018) Torymidae (Hymenoptera, Chalcidoidea) revised: molecular phylogeny, circumscription and reclassification of the family with discussion of its biogeography and evolution of life‐history traits. Cladistics, **34**(6), 627-651. |
| Johnson NF, Musetti L (2004) Catalog of systematic literature of the superfamily Ceraphronoidea (Hymenoptera). American Entomological Institute. |
| Klingenberg A, Ulber B (1994) Untersuchungen zum Auftreten der Tersilochinae (Hym., Ichneumonidae) als Larvalparasitoide einiger Rapsschädlinge im Raum Göttingen 1990 und 1991 und zu deren Schlupfabundanz nach unterschiedlicher Bodenbearbeitung. Journal of Applied Entomology **117**, 287–99. |
| Klopfstein S, Broad G, Urfer K, Vårdal H, Haraldseide H (2022) An interactive key to the European genera of Campopleginae (Hymenoptera, Ichneumonidae) and 20 new species for Sweden. Entomologisk Tidskrift, **143**, 121-156. |
| Masner L (1980) Key to genera of Scelionidae of the Holarctic region, with descriptions of new genera and species (Hymenoptera: Proctotrupoidea). The Memoirs of the Entomological Society of Canada, **112**(S113), 1-54. |
| Murchie AK, Smart LE, Williams IH (1997) Responses of *Dasineura brassicae* Winn. (Diptera: Cecidomyiidae) and its parasitoids *Platygaster subuliformis* Kieffer (Hymenoptera: Platygastridae) and *Omphale clypealis* Thomson (Hymenoptera: Eulophidae) to traps, baited with organic isothiocyanates, in the field. Journal of Chemical Ecology **23**, 917–26. |
| Schwarz M, Shaw MR (1998) Westem Palaearctic Cryptinae (Hymenoptera: Ichneumonidae) in the National Museums of Scotland, with nomenclatura changes, taxonomic notes, rearing records and special reference to the British check list. Part 1. Tribe Cryptini. Entomologist’s Gazette, **49**, 101-127. |
| Speyer W (1925) *Perilitus melanopus* Ruthe (Hym., Braconidae) als Imaginal parasitoid von Ceutorrhyncus quadridens Panz. Zugleich eine kurze Zusammenfassung unserer bisherigen Kenntnisse von Schlupfwespen als Parasiten der Käfer-Imagines. Zeitschrift für Angewandte Entomologie **11**, 132–46. |
| Tobias WJ, Belokobylskii SA, Kotenko AG (1986) Keys to the Fauna of the European Part of the USSR, Volume III, Hymenoptera, Part IV. Nauka: Leningrad. [In Russian] |
| Tomanović Ž, Žikić V, Petrović A (2019) Fauna of parasitoid wasps (Hymenoptera, Braconidae, Aphidiinae) of Serbia. Serbian Academy of Sciences and Arts, Monographs, 262 pp [in Serbian] |
| Tryapitzyn VA (1988). Keys to the Fauna of the European Part of the USSR, Volume III, Hymenoptera, Part II. E. J. Brill: Leiden. |
| Turotsi C (1990) The pteromalid fauna of Bulgaria. I. Pteromalinae (Hymenoptera, Pteromalidae). Acta Zoologica Bulgarica, **40**, 61-66. |
| Ulber B (2017) Identity of parasitoids and their potential for biocontrol of oilseed rape pests in Europe. EPPO Workshop on integrated management of insect pests in oilseed rape, Berlin 20-22th September 2017. |
| van Achterberg C (1997) Revision of the subfamily Euphorinae (excluding the tribe Meteorini Cresson)(Hymenoptera: Braconidae) from China. Zoologische verhandelingen, **313**(1), 1-217. |
| Vidal S, Müller J, Schmidt S (2022) Critical checklist of the Chalcidoidea and Mymarommatoidea (Insecta, Hymenoptera) of Germany. Biodiversity Data Journal, **10**. <https://doi.org/10.3897/BDJ.10.e85582> |
| Zerova MD (1995) Parasitic Hymenoptera – Eurytominae and Eudecatominae of Palaearctics. National Academy of Sciences of Ukraine, Kiev Naukova Dumka, Kiev. [In Russian] |
| Zerova MD, Seryognia LY (1999) Torymid chalcidoid wasps (Hymenoptera, Cahclidoidea, Torymidae) of the tribes Podagrionini and Monodontomerini of the Ukrainian fauna. Vestnik Zoologii: Supplement N **13**, 130 pp. [In Russian] |
