# Supplementary material for: Diversity of parasitoid wasps (Insecta, Hymenoptera) in oilseed rape fields in Serbia
Source: Biodivers Data J. 2023 Dec 5;11:e110118. doi: 10.3897/BDJ.11.e110118 (PMC10716848; doi:10.3897/BDJ.11.e110118)
Supplement: Supplementary material 2 — Supplement Table 2a. Checklist of parasitoids found in oilseed rape fields in Serbia [file bdj-11-e110118-s002.docx]

Supplement Tabel 2. Checklist of parasitoid wasp species. OSR - oilseed rape fields; SNH - semi-natural habitat. OSR pest host: possible - other species from that genus confirmed as OSR pest parasitoids.

| Family | **Taxon** | **Date** | **Locality** | **Habitat** | **Sampling methods** | **Host** | **OSR pest host** | **Parasitism** | **#M** | **#F** |
| --- | --- | --- | --- | --- | --- | --- | --- | --- | --- | --- |
| Bethylidae | *Goniozus*  *claripennis*  (Förster, 1851) | 22-24.04.2019 | Đurđin | OSR | Pan traps | Tortricidae | unknown | Primary | 1 | 0 |
|  | *Plastanoxus*  *westwoodi*  (Kieffer, 1914) | 24-27.04.2018 | Čenej | OSR | Pan traps | Cucujidae | unknown | Primary | 1 | 0 |
| Braconidae | *Apanteles*  sp. 1 | 07.05.2018,  10.05.2018,  25.05.2018,  18.04.2019,  12.06.2019 | Čenej, Mišićevo, Pačir | OSR, SNH | Sweep net | Lepidoptera | unknown | Primary | 8 | 5 |
|  | *Aphidius*  *ervi*  Haliday, 1834 | 24-27.04.2018,  04-07.05.2018,  07-10.05.2018,  25.05.2018,  22-24.04.2019,  23.05.2019 | Bajmok, Čenej, Đurđin, Srbobran | OSR, SNH | Sweep net,  Pan traps,  Aphid colony | *Myzus persicae* | yes | Primary | 215 | 60 |
|  | *Aphidius*  *matricariae*  Haliday, 1834 | 04.05.2018,  07.05.2018,  10.05.2018 | Čenej, Srbobran | OSR | Aphid colony | *Myzus persicae* | yes | Primary | 72 | 40 |
|  | *Aphidius*  sp. 1 | 23.05.2019 | Đurđin | OSR | Aphid colony | *Myzus persicae* | unknown, possible | Primary | 1 | 1 |
|  | *Binodoxys*  *angelicae*  (Haliday, 1833) | 23.05.2019 | Mišićevo | OSR | Aphid colony | *Myzus persicae* | yes | Primary | 1 | 0 |
|  | *Blacus*  *nigricornis*  Haeselbarth, 1973 | 27.04.2018,  07-10.05.2018 | Čenej, Srbobran | OSR, SNH | Sweep net,  Pan traps | *Meligethes aeneus* | yes | Primary | 2 | 1 |
|  | *Bracon*  *picticornis*  (Wesmael, 1838) | 17-19.04.2019 | Bajmok | OSR | Pan traps | *Meligethes aeneus* | yes | Primary | 1 | 0 |
|  | *Bracon*  *variator*  Nees, 1811 | 04-07.05.2018,  10.05.2018 | Čenej | OSR | Sweep net,  Pan traps | *Ceutorhynchus assimilis* | yes | Primary | 2 | 0 |
|  | *Chelonus*  *oculator*  (Fabricius, 1775) | 04-07.05.2018 | Srbobran | SNH | Pan traps | Lepidoptera, *Spodoptera exigua* | unknown | Primary | 1 | 0 |
|  | *Choeras*  *parasitellae*  (Bouché, 1834) | 10.05.2018 | Čenej | OSR | Sweep net | Lepidoptera | unknown | Primary | 0 | 1 |
|  | *Cotesia*  *glomerata*  (Linnaeus, 1758) | 27.04.2018,  04-07.05.2018,  25.05.2018 | Srbobran | OSR | Sweep net,  Pan traps | *Pieris* spp. | yes | Primary | 2 | 1 |
|  | *Cotesia*  *vestalis*  (Haliday, 1834) | 10.05.2019 | Pačir | SNH | Sweep net | *Plutella xylostella* | yes | Primary | 0 | 1 |
|  | *Dacnusa*  sp. 1 | 04-07.05.2018 | Čenej | OSR | Pan traps | *Phytomyza rufipes*? | unknown | Primary | 0 | 1 |
|  | *Diaeretiella*  *rapae*  (McIntosh,1855) | 24-27.04.201804-07.05.2018,  07-10.05.2018,  25.05.2018,  17-19.04.2019,  22-24.04.2019,  08.05.2019,  10.05.2019,  23.05.2019,  12.06.2019,  13.06.2019 | Bajmok, Čenej, Đurđin, Mišićevo, Pačir, Srbobran | OSR, SNH | Sweep net,  Pan traps,  Aphid colony | *Myzus persicae* | yes | Primary | 816 | 427 |
|  | *Diospilus*  *capito*  (Nees, 1834) | 27.04.2018 | Srbobran | SNH | Sweep net | *Meligethes aeneus* | yes | Primary | 0 | 1 |
|  | *Ephedrus*  *persicae*  Froggatt, 1904 | 10.05.2018 | Čenej | OSR | Aphid colony | *Myzus persicae* | yes | Primary | 1 | 0 |
|  | *Eubazus sigalphoides* (Marshall, 1889) | 24-27.04.2018,  04.05.2018,  07.05.2018,  10.05.2018,  25.05.2018,  27.04.2018 | Čenej, Srbobran | OSR | Sweep net,  Pan traps,  Aphid colony | *Meligethes aeneus* | yes | Primary | 44 | 8 |
|  | *Eubazus*  sp. 1 | 27.04.2018,  04.05.2018 | Srbobran | OSR, SNH | Sweep net | Curculionidae,  *Pissodes* spp. | unknown, possible | Primary | 1 | 1 |
|  | *Habrobracon*  *hebetor*  (Say, 1836) | 27.04.2018,  04-07.05.2018,  07-10.05.2018 | Čenej | OSR | Sweep net,  Pan traps | *Plutella xylostella* | yes | Primary | 3 | 0 |
|  | *Lysiphlebus*  *fabarum*  (Marshall, 1896) | 07-10.05.2018,  23.05.2019 | Pačir, Srbobran | SNH | Pan traps,  Aphid colony | *Myzus persicae* | yes | Primary | 7 | 8 |
|  | *Microctonus*  sp. 1 | 27.04.2018 | Čenej | OSR | Sweep net | *Psylliodes chrysocephala* | unknown, possible | Primary | 0 | 1 |
|  | *Microctonus*  sp. 2 | 10.05.2018 | Čenej | SNH | Sweep net | *Psylliodes chrysocephala* | unknown, possible | Primary | 1 | 0 |
|  | *Microplitis*  sp. 1 | 07.05.2018,  10.05.2018,  25.05.2018 | Čenej | OSR, SNH | Sweep net | Lepidoptera | unknown | Primary | 4 | 0 |
|  | *Peristenus*  sp. 1 | 25.04.2019,  10.05.2019 | Mišićevo, Pačir | SNH | Sweep net | Hemiptera,  Miridae | unknown | Primary | 2 | 0 |
|  | *Peristenus*  sp. 2 | 10.05.2019 | Pačir | SNH | Sweep net | Hemiptera,  Miridae | unknown | Primary | 1 | 0 |
|  | *Praon*  *volucre*  (Haliday, 1933) | 27.04.2018,  04-07.05.2018,  10.05.2018 | Čenej, Srbobran | OSR, SNH | Sweep net,  Pan traps,  Aphid colony | *Myzus persicae* | yes | Primary | 17 | 12 |
|  | *Schizoprymnus*  *obscurus*  (Nees, 1816) | 20.04.2018,  10.05.2018,  12.06.2019 | Čenej, Mišićevo | OSR, SNH | Sweep net,  Aphid colony | *Ceutorhynchus* spp. | yes | Primary | 6 | 0 |
|  | *Townesilitus*  *bicolor*  (Wesmael, 1835) | 24-27.04.2018,  07.05.2018,  10.05.2018,  25.05.2018,  10.05.2019 | Čenej, Pačir, Srbobran | OSR, SNH | Sweep net,  Pan traps | *Phyllotreta* spp. | yes | Primary | 29 | 4 |
|  | *Triaspis*  *thoracica*  (Curtis, 1860) | 27.04.2018,  07.05.2018,  10.05.2018,  25.05.2018 | Čenej, Srbobran | OSR | Sweep net | Chrysomelidae,  *Bruchus* spp. | unknown | Primary | 17 | 53 |
| Ceidae | *Cea*  *pulicaris*  Walker, 1837 | 07-10.05.2018 | Srbobran | SNH | Pan traps | Agromyzidae,  *Phytomyza* spp. | unknown | Primary | 1 | 0 |
|  | *Spalangiopelta*  sp. 1 | 27.04.2018,  04.05.2018,  07.05.2018,  25.05.2018,  22-24.04.2019,  25.04.2019,  12.06.2019 | Čenej, Đurđin, Mišićevo, Srbobran | OSR, SNH | Sweep net,  Pan traps | Agromyzidae,  Drosophilidae (miners) | unknown | Primary | 8 | 4 |
| Ceraphronidae | Ceraphronidae  sp. 1 | 24-27.04.2018,  07-10.05.2018,  25.05.2018 | Čenej, Srbobran | OSR, SNH | Sweep net, Pan traps | Cecidomyiidae,  Hemiptera,  Neuroptera,  Thysanoptera | unknown | Primary | 2 | 1 |
|  | Ceraphronidae  sp. 2 | 22-24.04.2019 | Đurđin | SNH | Pan traps | Cecidomyiidae,  Hemiptera,  Neuroptera,  Thysanoptera | unknown | Primary | 1 | 0 |
|  | *Aphanogmus*  *abdominalis*  (Thomson, 1858) | 24-27.04.2018,  04-07.05.2018,  07-10.05.2018,  13.06.2018 | Čenej, Srbobran | OSR, SNH | Sweep net,  Pan traps | *Dasineura brassicae* | yes | Primary | 9 | 16 |
|  | *Ceraphron*  sp. 1 | 04-07.05.2018 | Srbobran | SNH | Pan traps | Cecidomyiidae,  Hemiptera,  Neuroptera,  Thysanoptera | unknown | Primary | 1 | 0 |
|  | *Ceraphron*  sp. 2 | 24-27.04.2018,  22-24.04.2019 | Čenej, Đurđin | OSR | Pan traps | Cecidomyiidae,  Hemiptera,  Neuroptera,  Thysanoptera | unknown | Primary | 3 | 0 |
| Chalcididae | *Brachymeria*  *tibialis-*group  Steffan, 1958 | 24-27.04.2018,  24.04.2019 | Đurđin, Srbobran | OSR, SNH | Sweep net,  Pan traps | Lepidoptera,  Hymenoptera:  Diprionidae,  Diptera:  Cecidomyiidae | unknown | Primary, Secondary | 2 | 0 |
| Diapriidae | *Lyteba*  sp. 1 | 04-07.05.2018,  07-10.05.2018,  25.05.2018,  22-24.04.2019 | Bajmok, Pačir, Srbobran | OSR, SNH | Sweep net,  Pan traps | Diptera,  Mycetophilidae,  Sciaridae | unknown | Primary | 10 | 6 |
|  | *Trichopria*  sp. 1 | 24-27.04.2018 | Srbobran | SNH | Pan traps | Drosophilidae,  Sarcophagidae,  Sepsidae,  Muscidae,  Calliphoridae | unknown | Primary | 1 | 0 |
| Encyrtidae | Encyrtidae  sp. 1 | 07-10.05.2018 | Čenej | OSR | Pan traps | Hemiptera,  Homoptera:  Coccoidea,  Acarina | unknown | Primary, Secondary | 1 | 0 |
|  | Encyrtidae  sp. 2 | 22-24.04.2019 | Đurđin | SNH | Pan traps | Hemiptera  Homoptera:  Coccoidea,  Acarina | unknown | Primary, Secondary | 0 | 1 |
|  | Encyrtidae  sp. 3 | 25.04.2019 | Mišićevo | OSR, SNH | Sweep net | Hemiptera  Homoptera:  Coccoidea,  Acarina | unknown | Primary, Secondary | 0 | 2 |
|  | *Anagyrus*  sp. 1 | 22-24.04.2019 | Pačir | SNH | Pan traps | Hemiptera,  Pseudococcidae? | unknown | Primary | 1 | 2 |
|  | *Copidosoma*  *bakeri*  (Howard, 1898) | 04-07.05.2018,  07-10.05.2018,  22-24.04.2019 | Čenej, Pačir, Srbobran | OSR, SNH | Sweep net,  Pan traps | Noctuidae  (*Euxoa auxiliaris*) | unknown | Primary | 12 | 1 |
|  | *Eugahania*  *fumipennis*  (Ratzeburg, 1852) | 22-24.04.2019 | Bajmok | SNH | Pan traps | Cicadellidae,  *Macropsis vicina* | unknown | Primary | 1 | 0 |
|  | *Metaphycus*  *flavus*  (Ashmead, 1901) | 07.05.2018,  25.04.2019 | Mišićevo, Srbobran | OSR | Sweep net | Hemiptera,  Coccoidea | unknown | Primary | 3 | 0 |
|  | *Rhopus*  sp. 1 | 22-24.04.2019 | Đurđin | SNH | Pan traps | Hemiptera:  Pseudococcidae | unknown | Primary | 1 | 0 |
| Eulophidae | Eulophidae  sp. 1 | 18.04.2019,  10.05.2019 | Mišićevo, Pačir | OSR, SNH | Sweep net | Holometabolous insects | unknown | Primary, Secondary | 2 | 0 |
|  | Eulophidae  sp. 2 | 24-27.04.2018,  10.05.2018 | Čenej | OSR, SNH | Sweep net,  Pan traps | Holometabolous insects | unknown | Primary, Secondary | 1 | 1 |
|  | Eulophidae  sp. 3 | 07-10.05.2018,  25.05.2018 | Čenej, Mišićevo | OSR, SNH | Sweep net,  Pan traps,  Aphid colony | Holometabolous insects | unknown | Primary, Secondary | 7 | 3 |
|  | Eulophidae  sp. 4 | 07.05.2018,  12.06.2019 | Mišićevo, Srbobran | OSR | Sweep net | Holometabolous insects | unknown | Primary, Secondary | 2 | 0 |
|  | Eulophidae  sp. 5 | 08.05.2019 | Bajmok | OSR | Sweep net | Holometabolous insects | unknown | Primary, Secondary | 1 | 1 |
|  | Tetrastichinae  sp. 1 | 24-27.04.2018,  04-07.05.2018,  25.05.2018 | Čenej, Srbobran | OSR, SNH | Sweep net,  Pan traps | Holometabolous insects, spiders,  mites, nematodes | unknown | Primary, Secondary | 15 | 9 |
|  | Tetrastichinae  sp. 2 | 04-07.05.2018,  10.05.2018 | Čenej, Srbobran | OSR | Sweep net,  Pan traps | Holometabolous insects, spiders,  mites, nematodes | unknown | Primary, Secondary | 4 | 0 |
|  | *Aprostocetus*  *epicharmus*  (Walker, 1839) | 12.06.2019 | Mišićevo | SNH | Aphid colony | *Dasineura brassicae* | yes | Primary | 2 | 0 |
|  | *Aprostocetus*  sp. 1 | 07.05.2018,  10.05.2018,  25.05.2018,  25.04.2019,  08.05.2019 | Bajmok, Čenej, Mišićevo, Pačir, Srbobran | OSR, SNH | Sweep net,  Aphid colony | *Dasineura brassicae* | unknown, possible | Primary | 16 | 1 |
|  | *Diaulinopsis*  *arenaria*  (Erdös, 1951) | 24-27.04.2018,  04-07.05.2018,  10.05.2018,  25.05.2018 | Čenej, Srbobran | OSR, SNH | Sweep net,  Pan traps | *Liriomyza* spp. | unknown | Primary | 4 | 3 |
|  | *Diglyphus*  aff. *isaea* | 04-07.05.2018 | Srbobran | OSR | Pan traps | leaf miners | unknown | Primary | 0 | 1 |
|  | *Elasmus*  *platyedrae*  Ferrière, 1935 | 17-19.04.2019,  22-24.04.2019 | Bajmok, Pačir | SNH | Pan traps | Gelechiidae | unknown | Primary, Secondary | 7 | 0 |
|  | *Eulophus*  sp. 1 | 04.05.2018,  07.05.2018,  10.05.2018,  25.05.2018,  25.04.2019,  13.06.2019 | Bajmok, Čenej, Pačir, Srbobran | OSR, SNH | Sweep net | Cabbage Seed Weevil,  Lepidoptera | unknown, possible | Primary | 8 | 11 |
|  | *Necremnus*  sp. 1 | 17-19.04.2019 | Bajmok | SNH | Pan traps | Cabbage Seed Weevil,  Lepidoptera | unknown, possible | Primary | 0 | 1 |
|  | *Omphale*  *clypealis*  (Thomson, 1878) | 24-27.04.2018,  24.04.2019,  25.04.2019 | Bajmok, Čenej, Pačir | OSR | Sweep net,  Pan traps | *Dasineura brassicae* | yes | Primary | 3 | 0 |
|  | *Pnigalio*  sp. 1 | 24-27.04.2018,  25.04.2019 | Čenej, Pačir | OSR | Sweep net,  Pan traps | leaf miners:  Lepidoptera, Diptera,  Coleoptera,  Hymenoptera | unknown, possible | Primary, Secondary | 1 | 2 |
|  | *Tetrastichus*  sp. 1 | 24-27.04.2018,  04-07.05.2018,  07-10.05.2018,  25.05.2018,  25.04.2019 | Čenej, Mišićevo, Srbobran | OSR, SNH | Sweep net,  Pan traps,  Aphid colony | Buprestidae,  Cerambycidae,  Chrysomelidae  Curculionidae,  Lepidoptera,  Diptera,  Hymenoptera | unknown, possible | Primary | 9 | 8 |
| Eurytomidae | *Eurytoma*  sp. 1 | 27.04.2018,  04-07.05.2018,  07-10.05.2018,  25.05.2018,  25.04.2019 | Čenej, Mišićevo, Srbobran | OSR, SNH | Sweep net,  Pan traps,  Aphid colony | *Systole*,  *Bruchophagus*, hyperparasitoid  on *Tetramesa* | unknown, possible | Primary, Secondary | 4 | 7 |
| Figitidae | Eucolinae  sp. 1 | 24-27.04.2018,  07-10.05.2018,  22-24.04.2019 | Čenej, Đurđin, Srbobran | OSR, SNH | Sweep net,  Pan traps | Cyclorraphic dipterous larvae | unknown | Primary | 8 | 0 |
|  | Eucolinae  sp. 2 | 24-27.04.2018,  07-10.05.2018 | Čenej, Srbobran | OSR, SNH | Sweep net,  Pan traps | Cyclorraphic dipterous larvae | unknown | Primary | 2 | 3 |
|  | *Alloxysta*  sp. 1 | 04.05.2018,  07.05.2018 | Srbobran | OSR | Aphid colony | Aphidiinae,  Aphelininae,  Encyrtidae | unknown, possible | Secondary | 2 | 0 |
|  | *Rhoptomeris*  sp. 1 | 24-27.04.2018 | Čenej, Srbobran | OSR | Pan traps | Chloropidae,  Diptera | unknown | Primary | 2 | 1 |
| Ichneumonidae | Ichneumonidae  sp. 1 | 07.05.2018 | Srbobran | OSR | Sweep net | Holometabolous insects | unknown | Primary, Secondary | 0 | 1 |
|  | Phaeogenini  sp. 1 | 25.05.2018 | Srbobran | OSR | Sweep net | *Plutella xylostella* | unknown | Primary, Secondary | 0 | 1 |
|  | Phygadeuontinae  sp. 1 | 22-24.04.2019 | Đurđin | SNH | Pan traps | Holometabolous insects | unknown | Primary, Secondary | 0 | 2 |
|  | Phygadeuontini  sp. 1 | 10.05.2018 | Srbobran | OSR | Sweep net | Symphyta | unknown | Primary, Secondary | 0 | 1 |
|  | *Aneuclis*  *incidens*  (Thomson, 1889) | 24-27.04.2018,  10.05.2019 | Čenej, Pačir, Srbobran | OSR, SNH | Sweep net,  Pan traps | *Meligethes aeneus* | yes | Primary | 1 | 2 |
|  | *Aptesis*  *flagitator*  (Rossi, 1794) | 08.05.2019 | Bajmok | OSR | Sweep net | *Agonopterix heracliana*,  *Athalia spinarum* | yes | Primary | 1 | 0 |
|  | *Bathyplectes*  *curculionis*  (Thomson, 1887) | 27.04.2018 | Srbobran | SNH | Sweep net | *Apion pisi*,  *Hypera* spp. | unknown | Primary | 0 | 1 |
|  | *Collyria*  *coxator*  (Villers, 1789) | 04-07.05.2018,  10.05.2018,  25.05.2018,  22-24.04.2019,  08.05.2019 | Bajmok, Čenej, Đurđin, Pačir, Srbobran | OSR, SNH | Sweep net,  Pan traps | *Cephus cinctus*,  *Cephus pygmeus* | unknown | Primary | 7 | 14 |
|  | *Diadegma*  *insulare*  (Cresson, 1865) | 12.06.2019 | Mišićevo | OSR | Sweep net | *Plutella xylostella* | yes | Primary | 1 | 0 |
|  | *Diplazon*  *laetatorius*  (Fabricius, 1781) | 27.04.2018,  12.06.2019 | Čenej, Mišićevo | OSR, SNH | Sweep net,  Aphid colony | Diptera,  Syrphidae | unknown | Primary | 2 | 0 |
|  | *Dusona*  *pugillator*  (Linnaeus, 1758) | 22-24.04.2019,  10.05.2019 | Bajmok, Pačir | SNH | Sweep net,  Pan traps | Lepidoptera | unknown | Primary | 2 | 0 |
|  | *Diphyus*  *ochromelas*  (Gmelin, 1790) | 08.05.2019 | Bajmok | OSR | Sweep net | Lepidoptera | unknown | Primary | 1 | 0 |
|  | *Mesochorus*  sp. 1 | 10.05.2018 | Čenej | SNH | Sweep net | *Cotesia* spp. | unknown, possible | Secondary | 1 | 0 |
|  | *Olesicampe*  sp. 1 | 04-07.05.2018 | Srbobran | OSR | Pan traps | Tenthredinidae | unknown | Primary | 0 | 1 |
|  | *Stibeutes*  *curvispina*  (Thomson, 1884) | 04-07.05.2018,  07-10.05.2018,  22-24.04.2019 | Čenej, Đurđin, Srbobran | OSR | Sweep net,  Pan traps | *Ceutorhynchus pallidactylus* | yes | Primary | 3 | 2 |
|  | *Syrphophilus*  *bizonarius*  (Gravenhorst, 1829) | 10.05.2019 | Pačir | SNH | Sweep net | *Atherigona soccata,*  *Delia radicum,*  *Episyrphus balteatus,*  *Eupeodes corollae,*  *Eupeodes luniger,*  *Emex spinosa,*  *Loxostege sticticalis,*  *Neocnemodon vitripennis,*  *Sphaerophoria scripta* | unknown | Primary | 0 | 1 |
|  | *Tersilochus*  *heterocerus*  (Thomson, 1889) | 04.05.2018,  07.05.2018,  10.05.2018,  27.04.2018 | Čenej, Srbobran | OSR, SNH | Sweep net | *Meligethes aeneus* | yes | Primary | 11 | 7 |
|  | *Thrybius*  *praedator*  (Rossi, 1792) | 04.05.2018 | Srbobran | SNH | Sweep net | *Achnara spargani,*  *Chilo phragmitellus,*  *Oberea euphorbiae* | unknown | Primary | 0 | 1 |
| Megaspilidae | *Conostigmus*  *rufescens*  Kieffer, 1907 | 07.05.2018 | Srbobran | OSR | Sweep net | *Dasineura brassicae* | yes | Primary | 1 | 0 |
|  | *Lagynodes*  *pallidus*  (Boheman 1832) | 24-27.04.2018,  04-07.05.2018,  07-10.05.2018 | Čenej, Srbobran | OSR, SNH | Sweep net,  Pan traps | *Cotesia* spp. | unknown | Secondary | 9 | 6 |
| Mymaridae | Mymaridae  sp. 1 | 24-27.04.2018 | Srbobran | SNH | Pan traps | Auchenorrhynchous,  Hemiptera,  Coleoptera,  Psocoptera | unknown | Primary | 1 | 0 |
|  | *Anagrus*  sp. 1 | 20-22.04.2019,  24.04.2019,  25.04.2019 | Bajmok, Đurđin, Mišićevo | OSR, SNH | Sweep net,  Pan traps | Cicadellidae | unknown | Primary | 3 | 1 |
|  | *Anagrus*  sp. 2 | 04-07.05.2018 | Čenej, Srbobran | OSR, SNH | Pan traps | Cicadellidae | unknown | Primary | 2 | 0 |
|  | *Anagrus*  sp. 3 | 25.04.2019,  12.06.2019 | Mišićevo | OSR | Sweep net | Cicadellidae | unknown | Primary | 2 | 0 |
|  | *Anaphes*  sp. 1 | 24-27.04.2018,  04-07.05.2018,  10.05.2018,  22-24.04.2019,  25.04.2019 | Bajmok, Čenej, Đurđin, Mišićevo, Pačir, Srbobran | OSR, SNH | Sweep net,  Pan traps | Coleoptera:  Curculionidae,  Chysomelidae,  Hemiptera:  Miridae | unknown, possible | Primary | 27 | 6 |
|  | *Gonatocerus*  sp. 1 | 08.05.2019 | Bajmok | OSR | Sweep net | Cicadellidae | unknown | Primary | 1 | 1 |
|  | *Litus*  *cynipseus*  Haliday, 1833 | 04-07.05.2018,  10.05.2018 | Čenej, Srbobran | OSR, SNH | Sweep net,  Pan traps | Coleoptera,  Staphylinidae | unknown | Primary | 2 | 0 |
|  | *Lymaenon*  sp. 1 | 04.05.2018 | Srbobran | OSR | Sweep net | Cicadellidae,  Membracoidea | unknown | Primary | 1 | 1 |
|  | *Ooctonus*  sp. 1 | 04-07.05.2018,  07-10.05.2018 | Čenej, Srbobran | OSR, SNH | Sweep net,  Pan traps | Cercopoidea,  Cicadellidae | unknown | Primary | 1 | 2 |
|  | *Ooctonus*  *vulgatus*  Haliday, 1833 | 04-07.05.2018 | Čenej | OSR | Pan traps | *Philaenus leucophthalmus*,  *Philaenus spumarius* | unknown | Primary | 1 | 0 |
|  | *Polynema*  sp. 1 | 24-27.04.2018 | Čenej | OSR | Pan traps | Cicadellidae | unknown | Primary | 1 | 0 |
| Perilampidae | Perilampidae  sp. 1 | 13.06.2019 | Bajmok | OSR | Sweep net | Hymenoptera,  Diptera,  Coleoptera,  Lepidoptera,  Neuroptera | unknown | Primary, Secondary | 1 | 0 |
|  | *Chrysolampus*  *thenae*  (Walker, 1848) | 27.04.2018,  04.05.2018,  07.05.2018,  10.05.2018 | Čenej, Srbobran | OSR, SNH | Sweep net | *Meligethes pedicularis* | unknown | Primary | 17 | 12 |
|  | *Perilampus*  *aeneus*  (Rossius, 1790) | 13.06.2019 | Bajmok | OSR | Sweep net | *Athalia rosae* | yes | Primary | 0 | 1 |
| Pirenidae | *Macroglenes*  sp. 1 | 04-07.05.2018 | Čenej | OSR | Pan traps | Cecidomyiidae | unknown | Primary | 0 | 1 |
| Platygastridae | *Euxestonotus*  *error*  (Fitch, 1861) | 04-07.05.2018,  12.06.2019 | Čenej, Mišićevo | OSR | Oilseed pods,  Pan traps | *Sitodiplosis mosellana*,  *Dasineura brassicae*? | unknown | Primary | 2 | 0 |
|  | *Inostemma*  *boscii*  (Jurine, 1807) | 07-10.05.2018,  12.06.2019 | Čenej, Mišićevo | OSR, SNH | Sweep net,  Pan traps,  Aphid colony | *Dasineura brassicae* | yes | Primary | 3 | 0 |
|  | *Platygaster*  sp. 1 | 25.04.2019 | Mišićevo | SNH | Sweep net | Cecidomyiidae  (*Dasineura brassicae*?) | unknown, possible | Primary | 1 | 0 |
|  | *Platygaster*  sp. 2 | 07.05.2018,  17-19.04.2019,  22-24.04.2019 | Bajmok, Đurđin, Srbobran | OSR, SNH | Sweep net,  Pan traps | Cecidomyiidae  (*Dasineura brassicae*?) | unknown, possible | Primary | 5 | 1 |
|  | *Platygaster*  *subuliformis*  Kieffer, 1926 | 07-10.05.2018 | Čenej | OSR | Pan traps | *Dasineura brassicae* | yes | Primary | 1 | 0 |
|  | *Synopeas*  sp. 1 | 25.04.2019 | Mišićevo | SNH | Sweep net | Cecidomyiidae  (*Dasineura brassicae*?) | unknown, possible | Primary | 1 | 0 |
|  | *Telenomus*  sp. 1 | 24-27.04.2018,  04-07.05.2018,  07-10.05.2018,  22-24.04.2019,  25.04.2019,  12.06.2019,  13.06.2019 | Bajmok, Čenej, Đurđin, Mišićevo, Srbobran | OSR, SNH | Sweep net,  Pan traps | Lepidoptera,  Heteroptera,  Diptera,  Neuroptera | unknown | Primary | 25 | 6 |
|  | *Telenomus*  sp. 2 | 24-27.04.2018,  04-07.05.2018,  07-10.05.2018,  22-24.04.2019 | Đurđin, Srbobran | OSR, SNH | Pan traps | Lepidoptera,  Heteroptera,  Diptera,  Neuroptera | unknown | Primary | 5 | 3 |
|  | *Telenomus*  sp. 3 | 24-27.04.2018,  04-07.05.2018,  07-10.05.2018,  22-24.04.2019,  25.04.2019,  13.06.2019 | Bajmok, Čenej, Đurđin, Mišićevo, Pačir, Srbobran | OSR, SNH | Sweep net,  Pan traps | Lepidoptera,  Heteroptera,  Diptera,  Neuroptera | unknown | Primary | 17 | 2 |
|  | *Telenomus*  sp. 4 | 24-27.04.2018,  04-07.05.2018,  07-10.05.2018,  13.06.2019 | Bajmok, Čenej, Srbobran | OSR, SNH | Sweep net,  Pan traps | Lepidoptera,  Heteroptera,  Diptera,  Neuroptera | unknown | Primary | 15 | 6 |
| Pteromalidae | Pteromalidae  sp. 1 | 27.04.2018,  04-07.05.2018 | Čenej, Srbobran | OSR | Sweep net,  Pan traps | Lepidoptera,  Coleoptera,  Diptera | unknown | Primary, Secondary | 4 | 0 |
|  | Pteromalidae  sp. 2 | 24-27.04.2018 | Čenej | OSR | Pan traps | Lepidoptera,  Coleoptera,  Diptera | unknown | Primary, Secondary | 1 | 0 |
|  | Pteromalidae  sp. 3 | 04-07.05.2018 | Srbobran | SNH | Pan traps | Lepidoptera,  Coleoptera,  Diptera | unknown | Primary, Secondary | 1 | 0 |
|  | Pteromalidae  sp. 4 | 04.05.2018,  07.05.2018 | Čenej, Srbobran | OSR | Sweep net | Lepidoptera,  Coleoptera,  Diptera | unknown | Primary, Secondary | 2 | 0 |
|  | Pteromalidae  sp. 5 | 04-07.05.2018,  25.04.2019,  08.05.2019 | Čenej, Đurđin, Pačir, Srbobran | OSR | Sweep net,  Pan traps | Lepidoptera,  Coleoptera,  Diptera | unknown | Primary, Secondary | 8 | 0 |
|  | Pteromalidae  sp. 6 | 27.04.2018,  04.05.2018,  07.05.2018 | Čenej | OSR | Sweep net | Lepidoptera,  Coleoptera,  Diptera | unknown | Primary, Secondary | 3 | 0 |
|  | Pteromalidae  sp. 7 | 27.04.2018,  10.05.2018,  25.05.2018 | Čenej, Mišićevo, Srbobran | OSR, SNH | Sweep net | Lepidoptera,  Coleoptera,  Diptera | unknown | Primary, Secondary | 5 | 2 |
|  | Pteromalidae  sp. 8 | 04.05.2018,  10.05.2018,  24.04.2019 | Bajmok, Čenej, Srbobran | OSR | Sweep net | Lepidoptera,  Coleoptera,  Diptera | unknown | Primary, Secondary | 4 | 2 |
|  | Pteromalidae  sp. 9 | 27.04.2018,  10.05.2018 | Čenej | OSR, SNH | Sweep net | Lepidoptera,  Coleoptera,  Diptera | unknown | Primary, Secondary | 1 | 1 |
|  | Pteromalidae  sp. 10 | 07.05.2018 | Srbobran | OSR | Aphid colony | Lepidoptera,  Coleoptera,  Diptera | unknown | Primary, Secondary | 1 | 0 |
|  | Pteromalidae  sp. 11 | 17-19.04.2019 | Bajmok | SNH | Pan traps | Lepidoptera,  Coleoptera,  Diptera | unknown | Primary, Secondary | 0 | 1 |
|  | *Dibrachys*  *microgastri*  (Bouché, 1834) | 24-27.04.2018 | Srbobran | OSR | Pan traps | *Cotesia* spp. | unknown | Secondary | 1 | 0 |
|  | *Mesopolobus*  *incultus*  (Walker, 1834) | 25.04.2019,  12.06.2019,  13.06.2019 | Bajmok, Mišićevo, Pačir | OSR | Sweep net | Curculionidae:  *Gymnetron* sp.,  *Gymnetron pascuorum*,  *Mecinus* sp.,  Scolytidae:  *Polygraphus* *poligraphus*,  Agromyzidae:  *Phytobia humeralis*,  Cecidomyiidae:  *Kaltenbachiola strobi* | unknown | Primary | 4 | 1 |
|  | *Mesopolobus*  *morys* | 27.04.2018,  10.05.2018,  25.05.2018,  12.06.2019,  13.06.2019 | Bajmok, Čenej, Đurđin, Mišićevo, Srbobran | OSR, SNH | Sweep net,  Oilseed pods | *Ceutorhynchus assimilis* | yes | Primary | 23 | 5 |
|  | *Mesopolobus*  sp. 1 | 04-07.05.2018,  07-10.05.2018 | Čenej | OSR | Pan traps | *Ceutorhynchus* spp. | unknown, possible | Primary | 10 | 0 |
|  | *Mesopolobus*  sp. 2 | 24-27.04.2018,  10.05.2018 | Čenej, Srbobran | OSR | Sweep net,  Pan traps | *Ceutorhynchus* spp. | unknown, possible | Primary | 3 | 0 |
|  | *Mesopolobus*  sp. 3 | 04-07.05.2018,  10.05.2018 | Čenej, Srbobran | OSR, SNH | Sweep net,  Pan traps | *Ceutorhynchus* spp. | unknown, possible | Primary | 2 | 1 |
|  | *Pteromalus*  sp. 1 | 27.04.2018,  04.05.2018,  25.05.2018 | Čenej, Srbobran | OSR | Sweep net | Lepidoptera,  Tenthredinidae | unknown | Primary | 2 | 3 |
|  | *Pteromalus*  sp. 2 | 25.04.2019 | Mišićevo | SNH | Sweep net | Lepidoptera,  Tenthredinidae | unknown | Primary | 1 | 0 |
|  | *Pteromalus*  sp. 3 | 25.04.2019,  10.05.2019 | Mišićevo | SNH | Sweep net | Lepidoptera,  Tenthredinidae | unknown | Primary | 2 | 0 |
|  | *Trichomalus*  *lucidus*  (Walker, 1835) | 27.04.2018,  04-07.05.2018,  07-10.05.2018,  25.05.2018,  22-24.04.2019,  08.05.2019,  13.06.2019 | Bajmok, Čenej, Đurđin, Pačir, Srbobran | OSR, SNH | Sweep net,  Pan traps,  Aphid colony | *Ceutorhynchus* spp.,  *Psylliodes* *chrysocephala* | yes | Primary | 244 | 150 |
|  | *Trichomalus*  sp. 1 | 04-07.05.2018,  07-10.05.2018 | Čenej, Srbobran | OSR, SNH | Pan traps | *Ceutorhynchus* spp. | unknown, possible | Primary | 3 | 1 |
|  | *Trichomalus*  sp. 2 | 04-07.05.2018,  07-10.05.2018 | Čenej, Srbobran | OSR | Pan traps | *Ceutorhynchus* spp. | unknown, possible | Primary | 2 | 4 |
|  | *Trichomalus*  sp. 3 | 10.05.2018 | Čenej | OSR | Sweep net | *Ceutorhynchus* spp. | unknown, possible | Primary | 1 | 0 |
|  | *Trichomalus*  sp. 4 | 04-07.05.2018 | Srbobran | SNH | Pan traps | *Ceutorhynchus* spp. | unknown, possible | Primary | 1 | 0 |
| Scelionidae | Scelionidae  sp. 1 | 07.05.2018 | Srbobran | OSR | Sweep net | Insects,  arachnids | unknown | Primary | 0 | 1 |
|  | *Eumicrosoma*  sp. 1 | 07.05.2018 | Srbobran | OSR | Sweep net | Heteroptera,  Pentatomidae,  Lygaeidae | unknown | Primary | 1 | 0 |
|  | *Gryon*  sp. 1 | 24-27.04.2018,  07.05.2018,  10.05.2018 | Srbobran | OSR | Sweep net,  Pan traps | Hemiptera:  Coreidae | unknown | Primary | 1 | 2 |
|  | *Trimorus*  sp. 1 | 07.05.2018,  24.04.2019 | Bajmok, Srbobran | OSR | Sweep net | Carabidae | unknown | Primary | 1 | 1 |
|  | *Trissolcus*  *basalis*  (Wollaston, 1858) | 24-27.04.2018,  04-07.05.2018,  07-10.05.2018,  25.05.2018,  13.06.2019 | Bajmok, Čenej, Srbobran | OSR, SNH | Sweep net,  Pan traps | Heteroptera,  *Nezara viridula* | unknown | Primary | 14 | 5 |
| Spalangiidae | *Spalangia*  *nigra*  Latreille, 1805 | 07-10.05.2018,  22-24.04.2019 | Čenej, Pačir | OSR, SNH | Pan traps | Diptera puparia | unknown | Primary | 3 | 0 |
| Systasidae | *Asaphes*  *vulgaris*  Walker, 1834 | 10.05.2018,  25.05.2018 | Čenej, Srbobran | OSR | Sweep net | aphid parasitoids | yes | Secondary | 2 | 0 |
| Torymidae | *Podagrion pachymerum*  (Walker, 1833) | 25.04.2019 | Mišićevo | SNH | Sweep net | Mantodea,  Mantidae | unknown | Primary | 1 | 0 |
|  | *Pseudotorymus*  *napi*  (Amerling & Kirchner, 1860) | 24-27.04.2018,  04-07.05.2018,  07-10.05.2018,  25.05.2018,  25.05.2019 | Čenej, Mišićevo, Srbobran | OSR, SNH | Sweep net,  Pan traps,  Aphid colony,  Oilseed pods | *Dasineura brassicae* | yes | Primary | 144 | 74 |
|  | *Torymus*  sp. 1 | 25.04.2019 | Pačir | OSR | Sweep net | Ectoparasitoids of gall forming insects  (Cecidomyiidae,  Cynipidae) | unknown | Primary | 1 | 0 |
| Trichogrammatidae | Trichogrammatidae  sp. 1 | 24.04.2019 | Bajmok | OSR | Sweep net | Lepidoptera,  Coleoptera,  Neuroptera,  Diptera,  Hymenoptera | unknown | Primary | 1 | 0 |
|  | *Trichogramma*  *evanescens*  Westwood, 1833 | 24-27.04.2018 | Srbobran | SNH | Pan traps | Lepidoptera,  Chrysomelidae | unknown | Primary | 1 | 0 |
