# Supplementary material for: Diversity of parasitoid wasps (Insecta, Hymenoptera) in oilseed rape fields in Serbia
Source: Biodivers Data J. 2023 Dec 5;11:e110118. doi: 10.3897/BDJ.11.e110118 (PMC10716848; doi:10.3897/BDJ.11.e110118)
Supplement: Supplementary material 4 — Supplement Table 3. Checklist of parasitoids found in oilseed rape fields in Serbia with additional host information. [file bdj-11-e110118-s004.docx]

Supplement Tabel 3. Checklist of parasitoid wasp species with host refrences. OSR - oilseed rape fields; SNH - semi-natural habitat. OSR pest host: possible - other species from that genus confirmed as OSR pest parasitoids.

| **Familia** | **Taxon** | **Host** | **OSR pest host** | **Parasitism** | **Host stage** | **References** |
| --- | --- | --- | --- | --- | --- | --- |
| Bethylidae | *Goniozus*  *claripennis*  (Förster, 1851) | Tortricidae | unknown | Primary | larval | Bărbuceanu, D., & Andriescu, I. *Goniozus claripennis* (Förster 1851)(Hymenopetra: Bethylidae) As parasitoid of grape leaf-roller *Sparganothis pilleriana* (Den. Et Schiff.)(Lep: Tortricidae) larvae in southern vineyards of Romania. |
|  | *Plastanoxus*  *westwoodi*  (Kieffer, 1914) | Cucujidae | unknown | Primary | larval/pupal | Rahman, M. M., Islam, W., & Ahmed, K. N. (2008). Fertility life tables of *Plastanoxus westwoodi* (Kieffer)(Hymenoptera: Bethylidae) on *Cryptolestes pusillus* (Schon.)(Coleoptera: Cucujidae). Journal of Bio-Science, 16, 25-28. |
| Braconidae | *Apanteles*  sp. 1 | Lepidoptera | unknown | Primary | larval | Yu, D. S., van Achterberg, C. V., & Horstmann, K. (2012). Taxapad 2012, Ichneumonoidea 2011. Database on flash-drive. Ottawa, Ontario, Canada. |
|  | *Aphidius*  *ervi*  Haliday, 1834 | *Myzus persicae* | yes | Primary | larval | Žikić V, Ilić-Milošević M, Stanković S, Petrović A, Petrović-Obradović O, Kavallieratos NG, Starý P, Tomanović Ž (2012) Aphidiinae (Hymenoptera: Braconidae) of Serbia and Montenegro – tritrophic interactions. Acta entomologica serbica, 17(1/2): 83-105 |
|  | *Aphidius*  *matricariae*  Haliday, 1834 | *Myzus persicae* | yes | Primary | larval | Žikić V, Ilić-Milošević M, Stanković S, Petrović A, Petrović-Obradović O, Kavallieratos NG, Starý P, Tomanović Ž (2012) Aphidiinae (Hymenoptera: Braconidae) of Serbia and Montenegro – tritrophic interactions. Acta entomologica serbica, 17(1/2): 83-105 |
|  | *Aphidius*  sp. 1 | *Myzus persicae* | unknown, possible | Primary | larval | Žikić V, Ilić-Milošević M, Stanković S, Petrović A, Petrović-Obradović O, Kavallieratos NG, Starý P, Tomanović Ž (2012) Aphidiinae (Hymenoptera: Braconidae) of Serbia and Montenegro – tritrophic interactions. Acta entomologica serbica, 17(1/2): 83-105 |
|  | *Binodoxys*  *angelicae*  (Haliday, 1833) | *Myzus persicae* | yes | Primary | larval | Žikić V, Ilić-Milošević M, Stanković S, Petrović A, Petrović-Obradović O, Kavallieratos NG, Starý P, Tomanović Ž (2012) Aphidiinae (Hymenoptera: Braconidae) of Serbia and Montenegro – tritrophic interactions. Acta entomologica serbica, 17(1/2): 83-105 |
|  | *Blacus*  *nigricornis*  Haeselbarth, 1973 | *Meligethes aeneus* | yes | Primary | larval | Tobias, V.I. 1986. Euphorinae. Pp. 181-250. In: Medvedev G.S. (ed.) ‘Opredelitel Nasekomych Evrospeiskoi Tsasti SSSR 3, Peredpontdatokrylye 4. Opr. Faune SSSR.’ 145:1-501. Pp. 181-250. [Keys to the insects of the European part of USSR. Hymenoptera. 317-437.] |
|  | *Bracon*  *picticornis*  (Wesmael, 1838) | *Meligethes aeneus* | yes | Primary | larval | Yu, D. S., van Achterberg, C. V., & Horstmann, K. (2012). Taxapad 2012, Ichneumonoidea 2011. Database on flash-drive. Ottawa, Ontario, Canada. |
|  | *Bracon*  *variator*  Nees, 1811 | *Ceutorhynchus assimilis* | yes | Primary | larval | Poole, R.W.; Gentili, P. 1996. Nomina Insecta Nearctica. Volume 1: Coleoptera, Strepsiptera. Entomological Information Services. Rockville. U.S.A. 827 pp. |
|  | *Chelonus*  *oculator*  (Fabricius, 1775) | Lepidoptera, *Spodoptera exigua* | unknown | Primary | egg/larval | Yu, D. S., van Achterberg, C. V., & Horstmann, K. (2012). Taxapad 2012, Ichneumonoidea 2011. Database on flash-drive. Ottawa, Ontario, Canada. |
|  | *Choeras*  *parasitellae*  (Bouché, 1834) | Lepidoptera | unknown | Primary | larval | Papp, J. (2009). Contribution to the braconid fauna of the former Yugoslavia, V. Ten subfamilies (Hymenoptera, Braconidae). Entomofauna, 30(1), 1-36. |
|  | *Cotesia*  *glomerata*  (Linnaeus, 1758) | *Pieris* spp. | yes | Primary | larval | van Nouhuys, S., Shaw, M. R., & Stefanescu, C. (2009). Parasitoids of European butterflies. In Ecology of butterflies of Europe (pp. 130-156). Cambridge University Press. |
|  | *Cotesia*  *vestalis*  (Haliday, 1834) | *Plutella xylostella* | yes | Primary | larval | Girling, R. D., Stewart-Jones, A., Dherbecourt, J., Staley, J. T., Wright, D. J., & Poppy, G. M. (2011). Parasitoids select plants more heavily infested with their caterpillar hosts: a new approach to aid interpretation of plant headspace volatiles. Proceedings of the Royal Society B: Biological Sciences, 278(1718), 2646-2653. |
|  | *Dacnusa*  sp. 1 | *Phytomyza rufipes*? | unknown | Primary | larval | Tobias, V. I., Belokobylskiy, S., & Kotenko, A. (1986). Identification key to insects of the European part of the USSR. Hymenoptera, 4: 509 pp. |
|  | *Diaeretiella*  *rapae*  (McIntosh,1855) | *Myzus persicae* | yes | Primary | larval | Žikić V, Ilić-Milošević M, Stanković S, Petrović A, Petrović-Obradović O, Kavallieratos NG, Starý P, Tomanović Ž (2012) Aphidiinae (Hymenoptera: Braconidae) of Serbia and Montenegro – tritrophic interactions. Acta entomologica serbica, 17(1/2): 83-105 |
|  | *Diospilus*  *capito*  (Nees, 1834) | *Meligethes aeneus* | yes | Primary | larval | Linz, B. 1992. Hymenoteren als Parasitoides des Rapsglanzkäfers (*Meligethes* spp., Nitidulidae) und des Kohlschotenrüsslers (*Ceutorhynchus assimilis*, Curculionidae) auf Raps und Rüben. Mitteilungen der Deutschen Gesellschaft für Allgemeine und Angewandte Entomologie. 8(1-3) (1991): 90-92. |
|  | *Ephedrus*  *persicae*  Froggatt, 1904 | *Myzus persicae* | yes | Primary | larval | Žikić V, Ilić-Milošević M, Stanković S, Petrović A, Petrović-Obradović O, Kavallieratos NG, Starý P, Tomanović Ž (2012) Aphidiinae (Hymenoptera: Braconidae) of Serbia and Montenegro – tritrophic interactions. Acta entomologica serbica, 17(1/2): 83-105 |
|  | *Eubazus sigalphoides* (Marshall, 1889) | *Meligethes aeneus* | yes | Primary | larval | Tobias, V.I. 1986. Helconinae, Brachistinae. pp. 150-180. In: Medvedev G.S. (ed.) 'Opredelitel Nasekomych Evrospeiskoi Tsasti SSSR 3, Peredpontdatokrylye 4. Opr. Faune SSSR.' 145:1-501. pp. 150-180. |
|  | *Eubazus*  sp. 1 | Curculionidae,  *Pissodes* spp. | unknown, possible | Primary | larval | Yu, D. S., van Achterberg, C. V., & Horstmann, K. (2012). Taxapad 2012, Ichneumonoidea 2011. Database on flash-drive. Ottawa, Ontario, Canada. |
|  | *Habrobracon*  *hebetor*  (Say, 1836) | *Plutella xylostella* | yes | Primary | larval | Mukti N. Ghimire, Thomas W. Phillips, Suitability of Different Lepidopteran Host Species for Development of *Bracon hebetor* (Hymenoptera: Braconidae), Environmental Entomology, Volume 39, Issue 2, 1 April 2010, Pages 449–458, https://doi.org/10.1603/EN09213  Werner, F. G. (1982). Common names of insects & related organisms (No. 595.7 W4). |
|  | *Lysiphlebus*  *fabarum*  (Marshall, 1896) | *Myzus persicae* | yes | Primary | larval | Žikić V, Ilić-Milošević M, Stanković S, Petrović A, Petrović-Obradović O, Kavallieratos NG, Starý P, Tomanović Ž (2012) Aphidiinae (Hymenoptera: Braconidae) of Serbia and Montenegro – tritrophic interactions. Acta entomologica serbica, 17(1/2): 83-105 |
|  | *Microctonus*  sp. 1 | *Psylliodes chrysocephala* | unknown, possible | Primary | adult | Jordan, A., Broad, G. R., Stigenberg, J., Hughes, J., Stone, J., Bedford, I., Penfield, S. & Wells, R. (2020). The potential of the solitary parasitoid *Microctonus brassicae* for the biological control of the adult cabbage stem flea beetle, *Psylliodes chrysocephala*. Entomologia experimentalis et applicata, 168, 360-370. |
|  | *Microctonus*  sp. 2 | *Psylliodes chrysocephala* | unknown, possible | Primary | adult | Jordan, A., Broad, G. R., Stigenberg, J., Hughes, J., Stone, J., Bedford, I., Penfield, S. & Wells, R. (2020). The potential of the solitary parasitoid *Microctonus brassicae* for the biological control of the adult cabbage stem flea beetle, *Psylliodes chrysocephala*. Entomologia experimentalis et applicata, 168, 360-370. |
|  | *Microplitis*  sp. 1 | Lepidoptera | unknown | Primary | larval | Fernandez-Triana, J. L. (2010). Eight new species and an annotated checklist of Microgastrinae (Hymenoptera, Braconidae) from Canada and Alaska. ZooKeys, (63), 1. |
|  | *Peristenus*  sp. 1 | Hemiptera,  Miridae | unknown | Primary | adult | Yu, D. S., van Achterberg, C. V., & Horstmann, K. (2012). Taxapad 2012, Ichneumonoidea 2011. Database on flash-drive. Ottawa, Ontario, Canada. |
|  | *Peristenus*  sp. 2 | Hemiptera,  Miridae | unknown | Primary | adult | Yu, D. S., van Achterberg, C. V., & Horstmann, K. (2012). Taxapad 2012, Ichneumonoidea 2011. Database on flash-drive. Ottawa, Ontario, Canada. |
|  | *Praon*  *volucre*  (Haliday, 1933) | *Myzus persicae* | yes | Primary | larval | Žikić V, Ilić-Milošević M, Stanković S, Petrović A, Petrović-Obradović O, Kavallieratos NG, Starý P, Tomanović Ž (2012) Aphidiinae (Hymenoptera: Braconidae) of Serbia and Montenegro – tritrophic interactions. Acta entomologica serbica, 17(1/2): 83-105 |
|  | *Schizoprymnus*  *obscurus*  (Nees, 1816) | *Ceutorhynchus* spp. | yes | Primary | larval | Papp, J. 1998. Contribution to the Braconid fauna of Hungary, XIII. Calyptinae - 2., Helconinae (Hymenoptera, Braconidae). Folia Entomologica Hungarica. 59:163-184. |
|  | *Townesilitus*  *bicolor*  (Wesmael, 1835) | *Phyllotreta* spp. | yes | Primary | adult | Ekbom, B. 1990. Flea beetles (Phyllotreta spp.) in spring oilseed rape in Sweden. Bulletin SROP. 13(4): 57-61. |
|  | *Triaspis*  *thoracica*  (Curtis, 1860) | Chrysomelidae,  *Bruchus* spp. | unknown | Primary | larval | Brajkovic, M.; Krunic, M.; Tomanovic, Z. 1999. Some evolutionary aspects of labial palps in Braconidae (Hymenoptera). Glasnik Prirodnjackog Muzeja I Beogradu Seriya B Bioloske Nauke. 49-50, (1995-1998): 219-228. |
| Ceidae | *Cea*  *pulicaris*  Walker, 1837 | Agromyzidae,  *Phytomyza* spp. | unknown | Primary | larval | Mitroiu, M. D. (2016). Review of world genera of Ceinae, with the description of two new Palaearctic species of *Spalangiopelta* Masi (Hymenoptera, Chalcidoidea, Pteromalidae). European Journal of Taxonomy, (251). |
|  | *Spalangiopelta*  sp. 1 | Agromyzidae,  Drosophilidae (miners) | unknown | Primary | larval | Mitroiu, M. D. (2016). Review of world genera of Ceinae, with the description of two new Palaearctic species of *Spalangiopelta* Masi (Hymenoptera, Chalcidoidea, Pteromalidae). European Journal of Taxonomy, (251). |
| Ceraphronidae | Ceraphronidae  sp. 1 | Cecidomyiidae,  Hemiptera,  Neuroptera,  Thysanoptera | unknown | Primary | larval | Dessart P. 1978. Four new species of African Ceraphronidae (Hymenoptera). Journal of the Entomological Society of South Africa 41: 275–284.  Johnson, N.F. & Musetti, L. 2004. Catalog of systematic literature of the superfamily Ceraphronoidea (Hymenoptera). Contributions of the American Entomological Institute 33: 1-149.  Krzyzynski, M., & Ulrich, W. (2015). Ceraphronidae and Megaspilidae (Hymenoptera: Ceraphronoidea) of Poland: current state of knowledge with corrections to the Polish checklist. Polish Journal of Entomology, 84(3), 191. |
|  | Ceraphronidae  sp. 2 | Cecidomyiidae,  Hemiptera,  Neuroptera,  Thysanoptera | unknown | Primary | larval | Dessart P. 1978. Four new species of African Ceraphronidae (Hymenoptera). Journal of the Entomological Society of South Africa 41: 275–284.  Johnson, N.F. & Musetti, L. 2004. Catalog of systematic literature of the superfamily Ceraphronoidea (Hymenoptera). Contributions of the American Entomological Institute 33: 1-149.  Krzyzynski, M., & Ulrich, W. (2015). Ceraphronidae and Megaspilidae (Hymenoptera: Ceraphronoidea) of Poland: current state of knowledge with corrections to the Polish checklist. Polish Journal of Entomology, 84(3), 191. |
|  | *Aphanogmus*  *abdominalis*  (Thomson, 1858) | *Dasineura brassicae* | yes | Primary | larval | Broad, G., & Livermore, L. (2014). Checklist of British and Irish Hymenoptera-Ceraphronoidea. |
|  | *Ceraphron*  sp. 1 | Cecidomyiidae,  Hemiptera,  Neuroptera,  Thysanoptera | unknown | Primary | larval | Dessart P. 1978. Four new species of African Ceraphronidae (Hymenoptera). Journal of the Entomological Society of South Africa 41: 275–284.  Johnson, N.F. & Musetti, L. 2004. Catalog of systematic literature of the superfamily Ceraphronoidea (Hymenoptera). Contributions of the American Entomological Institute 33: 1-149. |
|  | *Ceraphron*  sp. 2 | Cecidomyiidae,  Hemiptera,  Neuroptera,  Thysanoptera | unknown | Primary | larval | Dessart P. 1978. Four new species of African Ceraphronidae (Hymenoptera). Journal of the Entomological Society of South Africa 41: 275–284.  Johnson, N.F. & Musetti, L. 2004. Catalog of systematic literature of the superfamily Ceraphronoidea (Hymenoptera). Contributions of the American Entomological Institute 33: 1-149. |
| Chalcididae | *Brachymeria*  *tibialis*-group  Steffan, 1958 | Lepidoptera,  Hymenoptera:  Diprionidae,  Diptera:  Cecidomyiidae | unknown | Primary, Secondary | larval/pupal | Noyes, J. S. (2003). Universal chalcidoidea database. http://www. nhm. ac. uk/entomology/chalcidoids/index. html. |
| Diapriidae | *Lyteba*  sp. 1 | Diptera,  Mycetophilidae,  Sciaridae | unknown | Primary | larval/pupal | Macek, J. (1995). Revision of West Palaearctic *Lyteba* (= *Oxylabis* auct.)(Hymenoptera: Proctotrupoidea, Diapriidae). Folia Heyrovskyana, 3(3), 29-39. |
|  | *Trichopria*  sp. 1 | Drosophilidae,  Sarcophagidae,  Sepsidae,  Muscidae,  Calliphoridae | unknown | Primary | larval/pupal | Häussling, B. J., Lienenlüke, J., & Stökl, J. (2021). The preference of *Trichopria drosophilae* for pupae of *Drosophila* *suzukii* is independent of host size. Scientific reports, 11(1), 1-10. |
| Encyrtidae | Encyrtidae  sp. 1 | Hemiptera,  Homoptera:  Coccoidea,  Acarina | unknown | Primary, Secondary | egg/larval | Yanzhou, Z., & Dawei, H. (2004). A review and an illustrated key to genera of Encyrtidae (Hymenoptera: Chalcidoidea) from China. Science Press. |
|  | Encyrtidae  sp. 2 | Hemiptera  Homoptera:  Coccoidea,  Acarina | unknown | Primary, Secondary | egg/larval | Yanzhou, Z., & Dawei, H. (2004). A review and an illustrated key to genera of Encyrtidae (Hymenoptera: Chalcidoidea) from China. Science Press. |
|  | Encyrtidae  sp. 3 | Hemiptera  Homoptera:  Coccoidea,  Acarina | unknown | Primary, Secondary | egg/larval | Yanzhou, Z., & Dawei, H. (2004). A review and an illustrated key to genera of Encyrtidae (Hymenoptera: Chalcidoidea) from China. Science Press. |
|  | *Anagyrus*  sp. 1 | Hemiptera,  Pseudococcidae? | unknown | Primary | egg | Hoffer, A. (1970). First contribution to the knowledge of the Yugoslavian Encyrtidae (Hem., Chalcidoidea). Studia Entomologica Forestalia, 1(10), 151-170. |
|  | *Copidosoma*  *bakeri*  (Howard, 1898) | Noctuidae  (*Euxoa auxiliaris*) | unknown | Primary | egg/larval | Guerrieri, E., & Noyes, J. (2005). Revision of the European species of *Copidosoma Ratzeburg* (Hymenoptera: Encyrtidae), parasitoids of caterpillars (Lepidoptera). Systematic Entomology, 30(1), 97-174. |
|  | *Eugahania*  *fumipennis*  (Ratzeburg, 1852) | Cicadellidae,  *Macropsis vicina* | unknown | Primary | larval | Trjapitzin, V.A. 1989, Parasitic Hymenoptera of the Fam. Encyrtidae of Palaearctics. Opredeliteli po Faune SSSR 158:374 Zoologicheskim Institutom Akademii Nauk SSR, Leningrad |
|  | *Metaphycus*  *flavus*  (Ashmead, 1901) | Hemiptera,  Coccoidea | unknown | Primary | egg | Kapranas, A., & Tena, A. (2015). Encyrtid parasitoids of soft scale insects: Biology, behavior, and their use in biological control. Annual review of entomology, 60, 195-211. |
|  | *Rhopus*  sp. 1 | Hemiptera:  Pseudococcidae | unknown | Primary | egg | Heng-Moss, T., Baxendale, F., & Riordan, T. (2001). Interactions between the parasitoid *Rhopus nigroclavatus* (Ashmead)(Hymenoptera: Encyrtidae) and its mealybug hosts *Tridiscus sporoboli* (Cockerell) and *Trionymus* sp.(Homoptera: Pseudococcidae). Biological Control, 22(3), 201-206. |
| Eulophidae | Eulophidae  sp. 1 | Holometabolous insects | unknown | Primary, Secondary | egg/larval/  pupal | Noyes J.S. (2003) – Universal Chalcidoidea Database, http://www.nhm.ac.uk/entomology/chalcidoids/. |
|  | Eulophidae  sp. 2 | Holometabolous insects | unknown | Primary, Secondary | egg/larval/  pupal | Noyes J.S. (2003) – Universal Chalcidoidea Database, http://www.nhm.ac.uk/entomology/chalcidoids/. |
|  | Eulophidae  sp. 3 | Holometabolous insects | unknown | Primary, Secondary | egg/larval/  pupal | Noyes J.S. (2003) – Universal Chalcidoidea Database, http://www.nhm.ac.uk/entomology/chalcidoids/. |
|  | Eulophidae  sp. 4 | Holometabolous insects | unknown | Primary, Secondary | egg/larval/  pupal | Noyes J.S. (2003) – Universal Chalcidoidea Database, http://www.nhm.ac.uk/entomology/chalcidoids/. |
|  | Eulophidae  sp. 5 | Holometabolous insects | unknown | Primary, Secondary | egg/larval/  pupal | Noyes J.S. (2003) – Universal Chalcidoidea Database, http://www.nhm.ac.uk/entomology/chalcidoids/. |
|  | Tetrastichinae  sp. 1 | Holometabolous insects, spiders,  mites, nematodes | unknown | Primary, Secondary | egg/larval/  pupal | La Salle J. (1994) – North American genera of Tetrastichinae (Hymenoptera: Eulophidae). Journal of Natural History, 28: 109-236. |
|  | Tetrastichinae  sp. 2 | Holometabolous insects, spiders,  mites, nematodes | unknown | Primary, Secondary | egg/larval/  pupal | La Salle J. (1994) – North American genera of Tetrastichinae (Hymenoptera: Eulophidae). Journal of Natural History, 28: 109-236. |
|  | *Aprostocetus*  *epicharmus*  (Walker, 1839) | *Dasineura brassicae* | yes | Primary | larval | Williams, I. H. (2003). Parasitoids of brassica pod midge. Biocontrol of Oilseed Rape Pests, 113. |
|  | *Aprostocetus*  sp. 1 | *Dasineura brassicae* | unknown, possible | Primary | larval | Williams, I. H. (2003). Parasitoids of brassica pod midge. Biocontrol of Oilseed Rape Pests, 113.  Yefremova, Z.; Ebrahimi, E.; Yegorenkova, E. 2007, The subfamilies Eulophinae, Entedoninae and Tetrastichinae in Iran, with description of new species (Hymenoptera, Eulophidae). Entomofauna 28(30):405 |
|  | *Diaulinopsis*  *arenaria*  (Erdös, 1951) | *Liriomyza* spp. | unknown | Primary | larval | Yefremova, Z.; Ebrahimi, E.; Yegorenkova, E. 2007, The subfamilies Eulophinae, Entedoninae and Tetrastichinae in Iran, with description of new species (Hymenoptera, Eulophidae). Entomofauna 28(30):405 |
|  | *Diglyphus*  aff. *isaea* | leaf miners | unknown | Primary | larval | Macek, J. (1995). Revision of West Palaearctic *Lyteba* (= *Oxylabis* auct.)(Hymenoptera: Proctotrupoidea, Diapriidae). Folia Heyrovskyana, 3(3), 29-39. |
|  | *Elasmus*  *platyedrae*  Ferrière, 1935 | Gelechiidae | unknown | Primary, Secondary | larval | Noyes, J. S. (2003). Universal chalcidoidea database. http://www. nhm. ac. uk/entomology/chalcidoids/index. html. |
|  | *Eulophus*  sp. 1 | Cabbage Seed Weevil,  Lepidoptera | unknown, possible | Primary | larval | Williams, I. H. (2003). Parasitoids of brassica pod midge. Biocontrol of Oilseed Rape Pests, 113.  Noyes, J. S. (2003). Universal chalcidoidea database. http://www. nhm. ac. uk/entomology/chalcidoids/index. html. |
|  | *Necremnus*  sp. 1 | Cabbage Seed Weevil,  Lepidoptera | unknown, possible | Primary | larval | Noyes, J. S. (2003). Universal chalcidoidea database. http://www. nhm. ac. uk/entomology/chalcidoids/index. html. |
|  | *Omphale*  *clypealis*  (Thomson, 1878) | *Dasineura brassicae* | yes | Primary | larval | Hansson, C., & Shevtsova, E. (2012). Revision of the European species of *Omphale* Haliday (Hymenoptera, Chalcidoidea, Eulophidae). ZooKeys, (232), 1. |
|  | *Pnigalio*  sp. 1 | leaf miners:  Lepidoptera, Diptera,  Coleoptera,  Hymenoptera | unknown, possible | Primary, Secondary | larval | Yoshimoto 1983; Askew 1984; Bernardo et al. 2006, 2007; Compton and Askew 2007; Grabenweger et al. 2009; Yefremova and Mistchenko 2009; Yegorenkova and Yefremova 2012; Strakhova et al. 2013; Yefremova et al. 2013, 2015; Noyes 2017 |
|  | *Tetrastichus*  sp. 1 | Buprestidae,  Cerambycidae,  Chrysomelidae  Curculionidae,  Lepidoptera,  Diptera,  Hymenoptera | unknown, possible | Primary | larval/pupal | Silva-Torres, C. S., Pontes, I. V., Torres, J. B., & Barros, R. (2010). New records of natural enemies of *Plutella xylostella* (L.)(Lepidoptera: Plutellidae) in Pernambuco, Brazil. Neotropical Entomology, 39(5), 835-838.;  Hansson, C., & Schmidt, S. (2020). A revision of European species of the genus *Tetrastichus* Haliday (Hymenoptera: Eulophidae) using integrative taxonomy. Biodiversity data journal, 8. |
| Eurytomidae | *Eurytoma*  sp. 1 | *Systole*,  *Bruchophagus*, hyperparasitoid  on *Tetramesa* | unknown, possible | Primary, Secondary | larval | Noyes, J. S. (2003). Universal chalcidoidea database. http://www. nhm. ac. uk/entomology/chalcidoids/index. html. |
| Figitidae | Eucolinae  sp. 1 | Cyclorraphic dipterous larvae | unknown | Primary | larval/pupal | Marchiori, C. H. (2017). Parasitoids of Diptera of forensic interest collected in Goiás, Brazil. International Journal of Research in Pharmacy and Biosciences, 4(1), 1-5. |
|  | Eucolinae  sp. 2 | Cyclorraphic dipterous larvae | unknown | Primary | larval/pupal | Marchiori, C. H. (2017). Parasitoids of Diptera of forensic interest collected in Goiás, Brazil. International Journal of Research in Pharmacy and Biosciences, 4(1), 1-5. |
|  | *Alloxysta*  sp. 1 | Aphidiinae,  Aphelininae,  Encyrtidae | unknown, possible | Secondary | larval | Grasswitz, T.R., Resse, B.D. Biology and host selection behaviour of the aphid hyperparasitoid *Alloxysta victrix* in association with the primary parasitoid *Aphidius colemani* and the host aphid *Myzus persicae*. BioControl 43, 261–271 (1998). https://doi.org/10.1023/A:1009987609371 |
|  | *Rhoptomeris*  sp. 1 | Chloropidae,  Diptera | unknown | Primary | larval/pupal | Parasites of Grass Flies (Diptera, Chloropidae)  Nartshuk, E. P. (2006). Parasites of grass flies (Diptera, Chloropidae) from the order Hymenoptera in the Holarctic region. Entomological Review, 86(5), 576-597. |
| Ichneumonidae | Ichneumonidae  sp. 1 | Holometabolous insects | unknown | Primary, Secondary | egg/larval | Yu DSK, van Achterberg C, Horstmann K. (2016) Taxapad 2016, Ichneumonoidea 2015. Database on flash-drive. Nepean, Ontario, Canada. |
|  | Phaeogenini  sp. 1 | *Plutella xylostella* | unknown | Primary, Secondary | larval | Rousse, P., & Simon van Noort, E. D. (2013). Revision of the Afrotropical Phaeogenini (Ichneumonidae, Ichneumoninae), with description of a new genus and twelve new species. ZooKeys, (354), 1. |
|  | Phygadeuontinae  sp. 1 | Holometabolous insects | unknown | Primary, Secondary | egg/larval | Yu DSK, van Achterberg C, Horstmann K. (2016) Taxapad 2016, Ichneumonoidea 2015. Database on flash-drive. Nepean, Ontario, Canada. |
|  | Phygadeuontini  sp. 1 | Symphyta | unknown | Primary, Secondary | larval | Kolarov, J., & Bordera, S. (2007). Fauna and distribution of Bulgarian Phygadeuontini (Hymenoptera: Ichneumonidae: Cryptinae). |
|  | *Aneuclis*  *incidens*  (Thomson, 1889) | *Meligethes aeneus* | yes | Primary | larval | Alford, D. V. (Ed.). (2008). Biocontrol of oilseed rape pests. John Wiley & Sons. |
|  | *Aptesis*  *flagitator*  (Rossi, 1794) | *Agonopterix heracliana*,  *Athalia spinarum* | yes | Primary | larval | Yu, D. S., van Achterberg, C. V., & Horstmann, K. (2012). Taxapad 2012, Ichneumonoidea 2011. Database on flash-drive. Ottawa, Ontario, Canada. |
|  | *Bathyplectes*  *curculionis*  (Thomson, 1887) | *Apion pisi*,  *Hypera* spp. | unknown | Primary | pupal | Yu, D. S., van Achterberg, C. V., & Horstmann, K. (2012). Taxapad 2012, Ichneumonoidea 2011. Database on flash-drive. Ottawa, Ontario, Canada. |
|  | *Collyria*  *coxator*  (Villers, 1789) | *Cephus cinctus*,  *Cephus pygmeus* | unknown | Primary | larval | Wahl, D. B., Shanower, T. G., & Hoelmer, K. A. (2007). A new species of *Collyria Schiødte* (Hymenoptera: Ichneumonidae: Collyriinae), a parasitoid of *Cephus* *fumipennis* (Hymenoptera: Cephidae) in China, and potential biological control agent for *Cephus* *cinctus* in North America. Journal of the Kansas Entomological Society, 80(1), 43-50. |
|  | *Diadegma*  *insulare*  (Cresson, 1865) | *Plutella xylostella* | yes | Primary | pupal | Lasota JA, Kok LT. 1986. *Diadegma insulare* (Hymenoptera: Ichneumonidae) parasitism of the diamondback moth (Lepidoptera: Plutellidae) in southwest Virginia. Journal of Entomological Science 21: 237-242. |
|  | *Diplazon*  *laetatorius*  (Fabricius, 1781) | Diptera,  Syrphidae | unknown | Primary | pupal | Townes, H. (1971). The Genera of Ichneumonidae, Part 4 (Vol. 17). Memoirs of the American Entomological Institute. |
|  | *Dusona*  *pugillator*  (Linnaeus, 1758) | Lepidoptera | unknown | Primary | larval | Yu, D. S., van Achterberg, C. V., & Horstmann, K. (2012). Taxapad 2012, Ichneumonoidea 2011. Database on flash-drive. Ottawa, Ontario, Canada. |
|  | *Diphyus*  *ochromelas*  (Gmelin, 1790) | Lepidoptera | unknown | Primary | larval | Gravenhorst, J. L. C. (1829). Ichneumonologia Europaea. Pars III. Vratislaviae, 1097 pp. |
|  | *Mesochorus*  sp. 1 | *Cotesia* spp. | unknown, possible | Secondary | larval | Riedel, M. (2018). The types of *Mesochorus* Gravenhorst (Hymenoptera, Ichneumonidae, Mesochorinae) described by W. Schwenke and deposited in the Senckenberg Deutsches Entomologisches Institut. Beiträge zur Entomologie= Contributions to Entomology, 68(1), 1-29. Ashfaq, M., Erlandson, M., & Braun, L. (2005). Hyperparasitism by *Mesochorus* spp.(Hymenoptera: Ichneumonidae) in *Peristenus* sp.(Hymenoptera: Braconidae) and development of PCR primers for hyperparasitoid detection. Biological Control, 32(3), 371-377. |
|  | *Olesicampe*  sp. 1 | Tenthredinidae | unknown | Primary | pupal | Eichhorn, O., & Pschorn‐Walcher, H. (1978). Biologie und Parasiten der Ebereschen‐Blattwespe, *Pristiphora geniculata* Htg.(Hym.: Tenthredinidae). Zeitschrift für angewandte Entomologie, 85(1‐4), 154-167. |
|  | *Stibeutes*  *curvispina*  (Thomson, 1884) | *Ceutorhynchus pallidactylus* | yes | Primary | larval | Horstmann, Klaus. 2010. [Revision of the European species of Stibeutes Foerster, 1850 (Hymenoptera, Ichneumonidae, Cryptinae).] Revision der europaeischen Arten von *Stibeutes* Foerster, 1850 (Hymenoptera, Ichneumonidae, Cryptinae). Entomofauna. 31 (15) :229-261. |
|  | *Syrphophilus*  *bizonarius*  (Gravenhorst, 1829) | *Atherigona soccata,*  *Delia radicum,*  *Episyrphus balteatus,*  *Eupeodes corollae,*  *Eupeodes luniger,*  *Emex spinosa,*  *Loxostege sticticalis,*  *Neocnemodon vitripennis,*  *Sphaerophoria scripta* | unknown | Primary | larval | Hasanshahi, G., Abbasipour, H., Jussila, R., Jahan, F., & Dosti, Z. (2013). First record of the genus and species, *Syrphophilus bizonarius* from Iran. Biocontrol in Plant Protection, 1(2), 111-113. |
|  | *Tersilochus*  *heterocerus* | *Meligethes aeneus* | yes | Primary | larval | Ulber, B., Williams, I. H., Klukowski, Z., Luik, A., & Nilsson, C. (2010). Parasitoids of oilseed rape pests in Europe: key species for conservation biocontrol. In Biocontrol-based integrated management of oilseed rape pests (pp. 45-76). Springer, Dordrecht. |
|  | *Thrybius*  *praedator*  (Rossi, 1792) | *Achnara spargani,*  *Chilo phragmitellus,*  *Oberea euphorbiae* | unknown | Primary | larval | Starke, H. (1956). Ichneumonidenfauna der sachsischen Oberlausitz. Nat Lusatica, 3, 17-92. |
| Megaspilidae | *Conostigmus*  *rufescens*  Kieffer, 1907 | *Dasineura brassicae* | yes | Primary | egg/larval | Alford, D. V. (Ed.). (2008). Biocontrol of oilseed rape pests. John Wiley & Sons. Muller, F. J., Baur, H., Gibson, G. A., Mason, P. G., & Kuhlmann, U. (2007). Review of the species of *Trichomalus* (Chalcidoidea: Pteromalidae) associated with *Ceutorhynchus* (Coleoptera: Curculionidae) host species of European origin. The Canadian Entomologist, 139(5), 643-657. |
|  | *Lagynodes*  *pallidus*  (Boheman 1832) | *Cotesia* spp. | unknown | Secondary | larval | Krzyzynski, M., & Ulrich, W. (2015). Ceraphronidae and Megaspilidae (Hymenoptera: Ceraphronoidea) of Poland: current state of knowledge with corrections to the Polish checklist. Polish Journal of Entomology, 84(3), 191. |
| Mymaridae | Mymaridae  sp. 1 | Auchenorrhynchous,  Hemiptera,  Coleoptera,  Psocoptera | unknown | Primary | egg | Noyes J.S. (2003) – Universal Chalcidoidea Database, http://www.nhm.ac.uk/entomology/chalcidoids/. |
|  | *Anagrus*  sp. 1 | Cicadellidae | unknown | Primary | egg | Baquero, E., & Jordana, R. (1999). Species of Anagrus Haliday, 1833 (Hymenoptera, Chalcidoidea, Mymaridae) in Navarra (Spain). Miscel· lània Zoològica, 39-50. |
|  | *Anagrus*  sp. 2 | Cicadellidae | unknown | Primary | egg | Baquero, E., & Jordana, R. (1999). Species of Anagrus Haliday, 1833 (Hymenoptera, Chalcidoidea, Mymaridae) in Navarra (Spain). Miscel· lània Zoològica, 39-50. |
|  | *Anagrus*  sp. 3 | Cicadellidae | unknown | Primary | egg | Baquero, E., & Jordana, R. (1999). Species of Anagrus Haliday, 1833 (Hymenoptera, Chalcidoidea, Mymaridae) in Navarra (Spain). Miscel· lània Zoològica, 39-50. |
|  | *Anaphes*  sp. 1 | Coleoptera:  Curculionidae,  Chysomelidae,  Hemiptera:  Miridae | unknown, possible | Primary | egg | Anderson, R. C., & Paschke, J. D. (1968). The biology and ecology of *Anaphes flavipes* (Hymenoptera: Mymaridae), an exotic egg parasite of the cereal leaf beetle. Annals of the Entomological Society of America, 61(1), 1-5.;  Zhu, Y. C., & Williams III, L. (2002). Detecting the egg parasitoid *Anaphes iole* (Hymenoptera: Mymaridae) in tarnished plant bug (Heteroptera: Miridae) eggs by using a molecular approach. Annals of the Entomological Society of America, 95(3), 359-365. |
|  | *Gonatocerus*  sp. 1 | Cicadellidae | unknown | Primary | egg | Triapitsyn, S. V. (2006). A key to the Mymaridae (Hymenoptera) egg parasitoids of proconiine sharpshooters (Hemiptera: Cicadellidae) in the Nearctic region, with description of two new species of *Gonatocerus*. Zootaxa, 1203(1), 1-38. |
|  | *Litus*  *cynipseus*  Haliday, 1833 | Coleoptera,  Staphylinidae | unknown | Primary | egg | Triapitsyn, S. V., & Berezovskiy, V. V. (2004). Review of the genus Litus Haliday, 1833 in the Holarctic and Oriental regions, with notes on the Palaearctic species of *Arescon* Walker, 1846 (Hymenoptera: Mymaridae). Far Eastern Entomologist, (141), 1-24. |
|  | *Lymaenon*  sp. 1 | Cicadellidae,  Membracoidea | unknown | Primary | egg | Aishan, Z., Triapitsyn, S. V., & Hu, H. Y. (2020). A review of the Chinese species of *Lymaenon* (Hymenoptera: Mymaridae), with description of six new species. Zootaxa, 4834(4), zootaxa-4834. |
|  | *Ooctonus*  sp. 1 | Cercopoidea,  Cicadellidae | unknown | Primary | egg | Huber, J. T. (2012). Revision of *Ooctonus* (Hymenoptera: Mymaridae) in the Nearctic region. The Journal of the Entomological Society of Ontario, 143. |
|  | *Ooctonus*  *vulgatus*  Haliday, 1833 | *Philaenus leucophthalmus*,  *Philaenus spumarius* | unknown | Primary | egg | Triapitsyn, S.V. (2010), Revision of the Palaearctic species and review of the Oriental species of *Ooctonus* (Hymenoptera: Mymaridae), with notes on extralimital taxa. Zootaxa 2381 pp. 1-74 |
|  | *Polynema*  sp. 1 | Cicadellidae | unknown | Primary | egg | Triapitsyn, S. V. (2021, May). Revised taxonomy of the common northern European fairyfly *Polynema* (*Doriclytus*) *atratum* Haliday, 1833 (Hymenoptera: Mymaridae), with fourteen new synonymies. In Annales Zoologici Fennici (Vol. 58, No. 1-3, pp. 87-107). Finnish Zoological and Botanical Publishing Board. |
| Perilampidae | Perilampidae  sp. 1 | Hymenoptera,  Diptera,  Coleoptera,  Lepidoptera,  Neuroptera | unknown | Primary, Secondary |  | Noyes J.S. (2003) – Universal Chalcidoidea Database, http://www.nhm.ac.uk/entomology/chalcidoids/. |
|  | *Chrysolampus*  *thenae*  (Walker, 1848) | *Meligethes pedicularis* | unknown | Primary | larval/pupal | Askew, R.R. 1980. The biology and larval morphology of *Chrysolampus thenae* (Walker) (Hymenoptera, Pteromalidae). Entomologist's Monthly Magazine 115:155-159. |
|  | *Perilampus*  *aeneus*  (Rossius, 1790) | *Athalia rosae* | yes | Primary | larval | Heraty, J.M. & Darling, D.C. 1984. Comparative morphology of the planidial larvae of Eucharitidae and Perilampidae (Hymenoptera: Chalcidoidea). Systematic Entomology 9(3):309-328. |
| Pirenidae | *Macroglenes*  sp. 1 | Cecidomyiidae | unknown | Primary | egg/larval | Mitroiu, M. D. (2010). Revision of the Palearctic species of *Macroglenes* Westwood (Hymenoptera: Pteromalidae). Zootaxa, 2563, 1-34. |
| Platygastridae | *Euxestonotus*  *error*  (Fitch, 1861) | *Sitodiplosis mosellana*,  *Dasineura brassicae*? | unknown | Primary | larval | Echegaray, E. R., Stougaard, R. N., & Bohannon, B. (2016). First record of *Euxestonotus error* (Hymenoptera: Platygastridae) in the Pacific Northwest, United States of America. The Canadian Entomologist, 148(5), 616-618. |
|  | *Inostemma*  *boscii*  (Jurine, 1807) | *Dasineura brassicae* | yes | Primary | egg/larval | Popovici, O. A., & Buhl, P. N. (2011). A short history regarding the taxonomy and systematic researches of Platygastroidea (Hymenoptera). Memoirs of the Scientific Sections of the Romanian Academy, 34(1), 65-104. |
|  | *Platygaster*  sp. 1 | Cecidomyiidae  (*Dasineura brassicae*?) | unknown, possible | Primary | larval | Talamas, E. J., Thompson, J., Cutler, A., Schoenberger, S. F., Cuminale, A., Jung, T., ... & Buffington, M. L. (2017). An online photographic catalog of primary types of Platygastroidea (Hymenoptera) in the National Museum of Natural History, Smithsonian Institution. Journal of Hymenoptera Research, 56, 187. |
|  | *Platygaster*  sp. 2 | Cecidomyiidae  (*Dasineura brassicae*?) | unknown, possible | Primary | larval | Talamas, E. J., Thompson, J., Cutler, A., Schoenberger, S. F., Cuminale, A., Jung, T., ... & Buffington, M. L. (2017). An online photographic catalog of primary types of Platygastroidea (Hymenoptera) in the National Museum of Natural History, Smithsonian Institution. Journal of Hymenoptera Research, 56, 187. |
|  | *Platygaster*  *subuliformis*  Kieffer, 1926 | *Dasineura brassicae* | yes | Primary | egg/larval | Murchie, A. K., Polaszek, A., & Williams, I. H. (1999). *Platygaster subuliformis* (Kieffer)(Hym., Platygastridae) new to Britain, an egg-larval parasitoid of the Brassica pod midge *Dasineura brassicae* Winnertz (Dipt., Cecidomyiidae). Entomologist's Monthly Magazine, 135(1624-7), 217-222. |
|  | *Synopeas*  sp. 1 | Cecidomyiidae  (*Dasineura brassicae*?) | unknown, possible | Primary | egg/larval | Buhl, P. N., & O'Connor, J. P. (2008). 23 species of Platygastrinae (Hymenoptera, Platygastridae) new to Ireland, including *Platygaster hibernica* sp. nov. and *Synopeas* *hibernicum* sp. nov. The Irish Naturalists' Journal, 111-115. |
|  | *Telenomus*  sp. 1 | Lepidoptera,  Heteroptera,  Diptera,  Neuroptera | unknown | Primary | egg | Huggert, L. (1983). On *Telenomus*, mainly European; redescriptions, new taxa, synonymies and combinations (Hymenoptera, Proctotrupoidea: Scelionidae). Insect Systematics & Evolution, 14(2), 145-167.;  Samin, N., Koçak, E., Ghahari, H., & Shojai, M. (2010). A checklist of Iranian *Telenomus* Haliday (Hymenoptera: Platygastroidae: Scelionidae: Telenominae). Linzer biologische Beiträge, 42(2), 1437-1444. |
|  | *Telenomus*  sp. 2 | Lepidoptera,  Heteroptera,  Diptera,  Neuroptera | unknown | Primary | egg | Huggert, L. (1983). On *Telenomus*, mainly European; redescriptions, new taxa, synonymies and combinations (Hymenoptera, Proctotrupoidea: Scelionidae). Insect Systematics & Evolution, 14(2), 145-167.;  Samin, N., Koçak, E., Ghahari, H., & Shojai, M. (2010). A checklist of Iranian *Telenomus* Haliday (Hymenoptera: Platygastroidae: Scelionidae: Telenominae). Linzer biologische Beiträge, 42(2), 1437-1444. |
|  | *Telenomus*  sp. 3 | Lepidoptera,  Heteroptera,  Diptera,  Neuroptera | unknown | Primary |  | Huggert, L. (1983). On *Telenomus*, mainly European; redescriptions, new taxa, synonymies and combinations (Hymenoptera, Proctotrupoidea: Scelionidae). Insect Systematics & Evolution, 14(2), 145-167.;  Samin, N., Koçak, E., Ghahari, H., & Shojai, M. (2010). A checklist of Iranian *Telenomus* Haliday (Hymenoptera: Platygastroidae: Scelionidae: Telenominae). Linzer biologische Beiträge, 42(2), 1437-1444. |
|  | *Telenomus*  sp. 4 | Lepidoptera,  Heteroptera,  Diptera,  Neuroptera | unknown | Primary | egg | Huggert, L. (1983). On *Telenomus*, mainly European; redescriptions, new taxa, synonymies and combinations (Hymenoptera, Proctotrupoidea: Scelionidae). Insect Systematics & Evolution, 14(2), 145-167.;  Samin, N., Koçak, E., Ghahari, H., & Shojai, M. (2010). A checklist of Iranian *Telenomus* Haliday (Hymenoptera: Platygastroidae: Scelionidae: Telenominae). Linzer biologische Beiträge, 42(2), 1437-1444. |
| Pteromalidae | Pteromalidae  sp. 1 | Lepidoptera,  Coleoptera,  Diptera | unknown | Primary, Secondary | larvae/pupae | Noyes J.S. (2003) – Universal Chalcidoidea Database, http://www.nhm.ac.uk/entomology/chalcidoids/. |
|  | Pteromalidae  sp. 2 | Lepidoptera,  Coleoptera,  Diptera | unknown | Primary, Secondary | larvae/pupae | Noyes J.S. (2003) – Universal Chalcidoidea Database, http://www.nhm.ac.uk/entomology/chalcidoids/. |
|  | Pteromalidae  sp. 3 | Lepidoptera,  Coleoptera,  Diptera | unknown | Primary, Secondary | larvae/pupae | Noyes J.S. (2003) – Universal Chalcidoidea Database, http://www.nhm.ac.uk/entomology/chalcidoids/. |
|  | Pteromalidae  sp. 4 | Lepidoptera,  Coleoptera,  Diptera | unknown | Primary, Secondary | larvae/pupae | Noyes J.S. (2003) – Universal Chalcidoidea Database, http://www.nhm.ac.uk/entomology/chalcidoids/. |
|  | Pteromalidae  sp. 5 | Lepidoptera,  Coleoptera,  Diptera | unknown | Primary, Secondary | larvae/pupae | Noyes J.S. (2003) – Universal Chalcidoidea Database, http://www.nhm.ac.uk/entomology/chalcidoids/. |
|  | Pteromalidae  sp. 6 | Lepidoptera,  Coleoptera,  Diptera | unknown | Primary, Secondary | larvae/pupae | Noyes J.S. (2003) – Universal Chalcidoidea Database, http://www.nhm.ac.uk/entomology/chalcidoids/. |
|  | Pteromalidae  sp. 7 | Lepidoptera,  Coleoptera,  Diptera | unknown | Primary, Secondary | larvae/pupae | Noyes J.S. (2003) – Universal Chalcidoidea Database, http://www.nhm.ac.uk/entomology/chalcidoids/. |
|  | Pteromalidae  sp. 8 | Lepidoptera,  Coleoptera,  Diptera | unknown | Primary, Secondary | larvae/pupae | Noyes J.S. (2003) – Universal Chalcidoidea Database, http://www.nhm.ac.uk/entomology/chalcidoids/. |
|  | Pteromalidae  sp. 9 | Lepidoptera,  Coleoptera,  Diptera | unknown | Primary, Secondary | larvae/pupae | Noyes J.S. (2003) – Universal Chalcidoidea Database, http://www.nhm.ac.uk/entomology/chalcidoids/. |
|  | Pteromalidae  sp. 10 | Lepidoptera,  Coleoptera,  Diptera | unknown | Primary, Secondary | larvae/pupae | Noyes J.S. (2003) – Universal Chalcidoidea Database, http://www.nhm.ac.uk/entomology/chalcidoids/. |
|  | Pteromalidae  sp. 11 | Lepidoptera,  Coleoptera,  Diptera | unknown | Primary, Secondary | larvae/pupae | Noyes J.S. (2003) – Universal Chalcidoidea Database, http://www.nhm.ac.uk/entomology/chalcidoids/. |
|  | *Dibrachys*  *microgastri*  (Bouché, 1834) | *Cotesia* spp. | unknown | Secondary | pupal | Peters, R. S., & Baur, H. (2011). A revision of the *Dibrachys* *cavus* species complex (Hymenoptera: Chalcidoidea: Pteromalidae). Zootaxa, 2937(1), 1-30. |
|  | *Mesopolobus*  *incultus*  (Walker, 1834) | Curculionidae:  *Gymnetron* sp.,  *Gymnetron pascuorum*,  *Mecinus* sp.,  Scolytidae:  *Polygraphus* *poligraphus*,  Agromyzidae:  *Phytobia humeralis*,  Cecidomyiidae:  *Kaltenbachiola strobi* | unknown | Primary | larval | Noyes J.S. (2003) – Universal Chalcidoidea Database, http://www.nhm.ac.uk/entomology/chalcidoids/. |
|  | *Mesopolobus*  *morys* | *Ceutorhynchus assimilis* | yes | Primary | larval | Ulber, B., Williams, I. H., Klukowski, Z., Luik, A., & Nilsson, C. (2010). Parasitoids of oilseed rape pests in Europe: key species for conservation biocontrol. In Biocontrol-based integrated management of oilseed rape pests (pp. 45-76). Springer, Dordrecht. |
|  | *Mesopolobus*  sp. 1 | *Ceutorhynchus* spp. | unknown, possible | Primary | larval | Baur, H., Muller, F. J., Gibson, G. A., Mason, P. G., & Kuhlmann, U. (2007). A review of the species of *Mesopolobus* (Chalcidoidea: Pteromalidae) associated with *Ceutorhynchus* (Coleoptera: Curculionidae) host-species of European origin. Bulletin of Entomological Research, 97(4), 387-397. |
|  | *Mesopolobus*  sp. 2 | *Ceutorhynchus* spp. | unknown, possible | Primary | larval | Baur, H., Muller, F. J., Gibson, G. A., Mason, P. G., & Kuhlmann, U. (2007). A review of the species of *Mesopolobus* (Chalcidoidea: Pteromalidae) associated with *Ceutorhynchus* (Coleoptera: Curculionidae) host-species of European origin. Bulletin of Entomological Research, 97(4), 387-397. |
|  | *Mesopolobus*  sp. 3 | *Ceutorhynchus* spp. | unknown, possible | Primary | larval | Baur, H., Muller, F. J., Gibson, G. A., Mason, P. G., & Kuhlmann, U. (2007). A review of the species of *Mesopolobus* (Chalcidoidea: Pteromalidae) associated with *Ceutorhynchus* (Coleoptera: Curculionidae) host-species of European origin. Bulletin of Entomological Research, 97(4), 387-397. |
|  | *Pteromalus*  sp. 1 | Lepidoptera,  Tenthredinidae | unknown | Primary | pupal | Klimmek, F., & Baur, H. (2018). An interactive key to Central European species of the *Pteromalus albipennis* species group and other species of the genus (Hymenoptera: Chalcidoidea: Pteromalidae), with the description of a new species. Biodiversity data journal, (6). |
|  | *Pteromalus*  sp. 2 | Lepidoptera,  Tenthredinidae | unknown | Primary | pupal | Klimmek, F., & Baur, H. (2018). An interactive key to Central European species of the *Pteromalus albipennis* species group and other species of the genus (Hymenoptera: Chalcidoidea: Pteromalidae), with the description of a new species. Biodiversity data journal, (6). |
|  | *Pteromalus*  sp. 3 | Lepidoptera,  Tenthredinidae | unknown | Primary | pupal | Klimmek, F., & Baur, H. (2018). An interactive key to Central European species of the *Pteromalus albipennis* species group and other species of the genus (Hymenoptera: Chalcidoidea: Pteromalidae), with the description of a new species. Biodiversity data journal, (6). |
|  | *Trichomalus*  *lucidus*  (Walker, 1835) | *Ceutorhynchus* spp.,  *Psylliodes* *chrysocephala* | yes | Primary | larval | Alford, D. V. (Ed.). (2008). Biocontrol of oilseed rape pests. John Wiley & Sons. Muller, F. J., Baur, H., Gibson, G. A., Mason, P. G., & Kuhlmann, U. (2007). Review of the species of *Trichomalus* (Chalcidoidea: Pteromalidae) associated with *Ceutorhynchus* (Coleoptera: Curculionidae) host species of European origin. The Canadian Entomologist, 139(5), 643-657. |
|  | *Trichomalus*  sp. 1 | *Ceutorhynchus* spp. | unknown, possible | Primary | larval | Muller, F. J., Baur, H., Gibson, G. A., Mason, P. G., & Kuhlmann, U. (2007). Review of the species of *Trichomalus* (Chalcidoidea: Pteromalidae) associated with *Ceutorhynchus* (Coleoptera: Curculionidae) host species of European origin. The Canadian Entomologist, 139(5), 643-657. |
|  | *Trichomalus*  sp. 2 | *Ceutorhynchus* spp. | unknown, possible | Primary | larval | Muller, F. J., Baur, H., Gibson, G. A., Mason, P. G., & Kuhlmann, U. (2007). Review of the species of *Trichomalus* (Chalcidoidea: Pteromalidae) associated with *Ceutorhynchus* (Coleoptera: Curculionidae) host species of European origin. The Canadian Entomologist, 139(5), 643-657. |
|  | *Trichomalus*  sp. 3 | *Ceutorhynchus* spp. | unknown, possible | Primary | larval | Muller, F. J., Baur, H., Gibson, G. A., Mason, P. G., & Kuhlmann, U. (2007). Review of the species of *Trichomalus* (Chalcidoidea: Pteromalidae) associated with *Ceutorhynchus* (Coleoptera: Curculionidae) host species of European origin. The Canadian Entomologist, 139(5), 643-657. |
|  | *Trichomalus*  sp. 4 | *Ceutorhynchus* spp. | unknown, possible | Primary | larval | Muller, F. J., Baur, H., Gibson, G. A., Mason, P. G., & Kuhlmann, U. (2007). Review of the species of *Trichomalus* (Chalcidoidea: Pteromalidae) associated with *Ceutorhynchus* (Coleoptera: Curculionidae) host species of European origin. The Canadian Entomologist, 139(5), 643-657. |
| Scelionidae | Scelionidae  sp. 1 | Insects,  arachnids | unknown | Primary |  | Orr, D. B. (1988). Scelionid wasps as biological control agents: a review. Florida Entomologist, 506-528. |
|  | *Eumicrosoma*  sp. 1 | Heteroptera,  Pentatomidae,  Lygaeidae | unknown | Primary | egg | Wright, R. J., & Danielson, S. D. (1992). First report of the chinch bug (Heteroptera: Lygaeidae) egg parasitoid *Eumicrosoma beneficum* Gahan (Hymenoptera: Scelionidae) in Nebraska. Journal of the Kansas Entomological Society, 346-348. |
|  | *Gryon*  sp. 1 | Hemiptera:  Coreidae | unknown | Primary | egg | Cornelius, M. L., Vinyard, B. T., Mowery, J. D., & Hu, J. S. (2020). Ovipositional Behavior of the Egg Parasitoid *Gryon* *pennsylvanicum* (Hymenoptera: Scelionidae) on Two Squash Bug Species *Anasa* *tristis* (Hemiptera: Coreidae) and *Anasa* *armigera*: Effects of Parasitoid Density, Nutrition, and Host Egg Chorion on Parasitism Rates. Environmental Entomology, 49(6), 1307-1315. |
|  | *Trimorus*  sp. 1 | Carabidae | unknown | Primary | egg | Veenakumari, K., Rajmohana, K., & Mohanraj, P. (2013). A new species of *Trimorus* (*Neotrimorus*)(Hymenoptera: Platygastridae) from Andaman Islands, India. Journal of Biological control, 27(4), 243-246. |
|  | *Trissolcus*  *basalis* | Heteroptera,  *Nezara viridula* | unknown | Primary | egg | Afonin et al., 2008; O’Connor, J. P., & Notton, D. G. (2013). A review of the Irish scelionids (Hymenoptera: Platygastroidea, Platygastridae) including four species new to Ireland. Bulletin of the Irish Biogeographical Society, 37, 20-44. |
| Spalangiidae | *Spalangia*  *nigra*  Latreille, 1805 | Diptera puparia | unknown | Primary | pupal | Klunker, R. (1994). The occurrence of puparium parasitoids as natural enemies of house flies. Applied Parasitology, 35(1), 36-50. |
| Systasidae | *Asaphes*  *vulgaris*  Walker, 1834 | aphid parasitoids | yes | Secondary | larval | Noyes, J. S. (2003). Universal chalcidoidea database. http://www. nhm. ac. uk/entomology/chalcidoids/index. html. |
| Torymidae | *Podagrion pachymerum*  (Walker, 1833) | Mantodea,  Mantidae | unknown | Primary | egg | Thompson, W.R. 1958, A catalogue of the parasites and predators of insect pests. Section 2. Host parasite catalogue, Part 5. pp.663 Commonwealth Agricultural Bureaux, Commonwealth Institute of Biological Control, Ottawa, Ontario, Canad |
|  | *Pseudotorymus*  *napi*  (Amerling & Kirchner, 1860) | *Dasineura brassicae* | yes | Primary | larval | Czajkowska, M. 1978, Investigations on the brassica pod midge (*Dasyneura brassicae* Winn.). IV. Species composition and importance of parasites. Roczniki Nauk Rolniczych (Seria E, Ochrana Roslin) 8(2):159-175. Grissell, E.E. 1995, Toryminae (Hymenoptera: Chalcidoidea: Torymidae): a redefinition, generic classification and annotated world catalogue of species. Memoirs on Entomology, International 2:259 (Host identification needs confirmation) |
|  | *Torymus*  sp. 1 | Ectoparasitoids of gall forming insects  (Cecidomyiidae,  Cynipidae) | unknown | Primary | larval | de Vere, M. W. R., & Gijswijt, M. J. (1998). Revision of the European species of *Torymus* Dalman (Hymenoptera: Torymidae). Zoologische Verhandelingen, 317(1), 1-202. |
| Trichogrammatidae | Trichogrammatidae  sp. 1 | Lepidoptera,  Coleoptera,  Neuroptera,  Diptera,  Hymenoptera | unknown | Primary | egg | Noyes, J. S. (2003). Universal chalcidoidea database. http://www. nhm. ac. uk/entomology/chalcidoids/index. html. |
|  | *Trichogramma*  *evanescens*  Westwood, 1833 | Lepidoptera,  Chrysomelidae | unknown | Primary | egg | Noyes, J. S. (2003). Universal chalcidoidea database. http://www. nhm. ac. uk/entomology/chalcidoids/index. html. |
